# Characterization, comparison, and optimization of lattice light sheets

**DOI:** 10.1126/sciadv.ade6623

**Published:** 2023-03-31

**Authors:** Gaoxiang Liu, Xiongtao Ruan, Daniel E. Milkie, Frederik Görlitz, Matthew Mueller, Wilmene Hercule, Alison Killilea, Eric Betzig, Srigokul Upadhyayula

**Affiliations:** ^1^ Department of Molecular and Cell Biology, University of California, Berkeley, Berkeley, CA 94720, USA.; ^2^ Howard Hughes Medical Institute, Janelia Research Campus, Ashburn, VA 20417, USA.; ^3^ Department of Physics, Howard Hughes Medical Institute, Helen Wills Neuroscience Institute, University of California, Berkeley, Berkeley, CA 94720, USA.; ^4^ Molecular Biophysics and Integrated Bioimaging Division, Lawrence Berkeley National Laboratory, Berkeley, CA 94720, USA.; ^5^ Chan Zuckerberg Biohub, San Francisco, CA 94158, USA.

## Abstract

Lattice light sheet microscopy excels at the noninvasive imaging of three-dimensional (3D) dynamic processes at high spatiotemporal resolution within cells and developing embryos. Recently, several papers have called into question the performance of lattice light sheets relative to the Gaussian sheets most common in light sheet microscopy. Here, we undertake a theoretical and experimental analysis of various forms of light sheet microscopy, which demonstrates and explains why lattice light sheets provide substantial improvements in resolution and photobleaching reduction. The analysis provides a procedure to select the correct light sheet for a desired experiment and specifies the processing that maximizes the use of all fluorescence generated within the light sheet excitation envelope for optimal resolution while minimizing image artifacts and photodamage. We also introduce a new type of “harmonic balanced” lattice light sheet that improves performance at all spatial frequencies within its 3D resolution limits and maintains this performance over lengthened propagation distances allowing for expanded fields of view.

## Teaser

Comparing conventional and lattice light sheets reveals the advantages of the latter and how to improve their performance further.

## INTRODUCTION

In light sheet microscopy (*1*), a thin plane of light is scanned along a *z* axis perpendicular to its direction of confinement through a specimen, and a stack of 2D (two-dimensional) fluorescence images acquired at successive planes is assembled into a 3D image. This substantially reduces photobleaching and phototoxicity in live specimens compared to traditional 3D widefield and confocal microscopy, which also require acquisition of an image stack, but do so while illuminating the entire thickness of the specimen. Coupled with modern high-speed cameras, light sheet microscopy excels in the rapid imaging of large cleared tissue volumes and developmental processes in whole transparent embryos.

The thickness of the light sheet contributes to the resolution in *z* and determines the degree of rejection of out-of-focus light, whereas the distance over which the thickness remains close to its minimum dictates the effective field of view (FOV). The most common light sheets have a Gaussian cross-sectional profile for which the FOV shrinks quadratically as the thickness is decreased to improve axial resolution and optical sectioning. Thus, these light sheets are most often used for multicellular imaging in the wide FOV limit of anisotropic resolution [e.g., axial resolution ~4× poorer than lateral resolution at an imaging numerical aperture (NA) of 1.0].

In the past decade, a number of beam-shaping methods have been used to craft light sheets capable of achieving higher axial resolution. Notable among these is lattice light sheet microscopy [LLSM; (*2*)], which uses a spatially structured light sheet with axially narrow maxima to achieve substantially higher axial resolution than a Gaussian light sheet of comparable FOV. It, therefore, excels at noninvasive 4D imaging of subcellular dynamics with speed, non-invasiveness, and axial resolution superior to confocal microscopy. LLSM has elucidated a wide range of biological processes, including T cell engagement with target cells (*3*), microtubule dynamics during cell division (*4*), organelle-organelle interactions in living cells (*5*), cellular uptake of extracellular fluid (*6*), macrophage migration and cancer cell extravasation in live zebrafish (*7*), single-molecule transcription factor kinetics in organoids (*8*), and structural heterogeneity within otherwise liquid-like RNA granules (*9*). Commercial versions of LLSM are now available.

Recently, several papers have questioned the ability of square lattices to produce light sheets having practical axial resolution superior to Gaussian light sheets of comparable length (*10*, *11*) and of hexagonal lattices to produce light sheet images having minimal artifacts, due to strong sidelobes and localized troughs in the overall optical transfer function (OTF) (*11*, *12*). Here, we argue that these assertions are consequences of the specific conditions chosen and assumptions made and demonstrate both theoretically and through live cell imaging conditions under which both square and hexagonal LLSs can provide faithful representations of sample structure at a resolution superior to Gaussian or sinc light sheets of comparable length. We also introduce further refinements to LLS design that improve their ability to maintain optimal resolution over long propagation distances.

## RESULTS AND DISCUSSION

### General theoretical considerations

A 3D electric field pattern $E_{\mathrm{exc}}^{\mathrm{fixed}}(x)$ that is weakly confined in its propagation (*y* direction) and strongly confined in the *z* axis of fluorescence detection can be moved in the *x* ⊥ *y*, *z* direction to illuminate a specimen with a sheet of light. In the scalar approximation, $E_{\mathrm{exc}}^{\mathrm{fixed}}(x)$ is given by the coherent superposition of plane waves converging to the focal point ***x*** = 0 of an excitation lens of focal length *F* and numerical aperture NA_pupil_ from every point *x_p_*, *z_p_* within the radius *a* = NA_pupil_*F* of the rear pupil of the lens. Thus, when the lens is excited with an input electric field *E*_pupil_(*x_p_*, *z_p_*)

$$E_{\mathrm{exc}}^{\mathrm{fixed}}(x)=\iint_{pupil}E_{\mathrm{pupil}}(x_{p},z_{p})\exp[ik(x_{p},z_{p})\cdot x]dx_{p}dz_{p}$$ (1a)

where the components of the wave vector ***k*** are related to the position in the pupil by

$$\begin{array}{r} (k_{x},k_{z})=k_{o}\frac{\left(x_{p},z_{p}\right)}{F}=k_{o}{\mathrm{NA}}_{\mathrm{pupil}}\frac{\left(x_{p},z_{p}\right)}{a}\;\mathrm{and}\;k_{o}=2\pi /\lambda_{\mathrm{exc}}=k/n \end{array}$$ (1b)

$$k_{y}=k_{y}(k_{x},k_{z})=\sqrt{k^{2}-k_{x}^{2}-k_{z}^{2}}$$ (1c)

Since *E*_pupil_(*x_p_*, *z_p_*) = 0 for $\sqrt{x_{p}^{2}+z_{p}^{2}}>a$, the integrals can be extended to infinity, and Eq. 1a can be expressed as

$$E_{\mathrm{exc}}^{\mathrm{fixed}}(x)\propto {\mathrm{FT}}_{k_{x}k_{z}}^{-1}\{E_{\mathrm{pupil}}(k_{x},k_{z})\exp(ik_{y}y)\}$$ (1d)

where ${\mathrm{FT}}_{\alpha }^{-1}$ refers to an inverse Fourier transform (FT) over variable α.

The point spread function (PSF) of the stationary intensity pattern in the specimen corresponding to $E_{\mathrm{exc}}^{\mathrm{fixed}}(x)$ is given by

$${\mathrm{PSF}}_{\mathrm{exc}}^{\mathrm{fixed}}(x)=E_{\mathrm{exc}}^{\mathrm{fixed}}(x)\cdot \bar{E_{\mathrm{exc}}^{\mathrm{fixed}}(x)}$$ (2a)

which has a frequency distribution in any *xz* plane along *y* defined by its OTF

$${\mathrm{OTF}}_{\mathrm{exc}}^{\mathrm{fixed}}(k_{x},y,k_{z})={\mathrm{FT}}_{xz}\{{\mathrm{PSF}}_{\mathrm{exc}}^{\mathrm{fixed}}(x)\}$$ (2b)

where ¯ denotes the complex conjugate. Inserting Eq. 2a into Eq. 2b and using the convolution theorem give

$${\mathrm{OTF}}_{\mathrm{exc}}^{\mathrm{fixed}}(k_{x},y,k_{z})={\mathrm{FT}}_{xz}\{E_{\mathrm{exc}}^{\mathrm{fixed}}(x)\}{⊗}_{k_{x}k_{z}}{\mathrm{FT}}_{xz}\{\bar{E_{\mathrm{exc}}^{\mathrm{fixed}}(x)}\}$$ (2c)

Further inserting Eq. 1c then gives

$${\mathrm{OTF}}_{\mathrm{exc}}^{\mathrm{fixed}}(k_{x},y,k_{z})\propto \{E_{\mathrm{pupil}}(k_{x},k_{z})\exp(ik_{y}y)\}{⊗}_{k_{x}k_{z}}\bar{\left\{E_{\mathrm{pupil}}(k_{x},k_{z})\exp(ik_{y}y)\right\}}$$ (3)

At the focal plane (*y* = 0), this reduces to the well-known result that the excitation OTF is the autocorrelation of the pupil electric field.

#### Creating a light sheet from a laterally confined, axially long beam

There are at least four ways in which $E_{\mathrm{exc}}^{\mathrm{fixed}}(x)$ can be moved across the *xy* FOV to acquire each *z* plane of a light sheet image volume: (i) continuously sweeping it across the FOV, (ii) moving it in discrete steps while using confocal slit detection, (iii) moving it in discrete steps and reconstructing an image via incoherent 3D structured illumination microscopy [SIM; (*13*)], and (iv) creating a coherent periodic array of identical copies of $E_{\mathrm{exc}}^{\mathrm{fixed}}(x)$, moving the array in discrete steps over one period, and reconstructing an image via coherent 3D SIM. We derive analytically the optical properties of all four in note S1.

#### Theoretical resolution limits

The theoretical resolution limit of a linear optical microscope is defined by its “support,” the boundary at which OTF_overall_(***k***) drops to zero. This support is a 2D surface in 3D space. The theoretical resolution $R({\hat{e}}_{k})$ in any particular direction ${\hat{e}}_{k}$ is determined by the magnitude *k* of the vector $k=k{\hat{e}}_{k}$ from the origin of OTF_overall_(***k***) to this surface. There are several directions ${\hat{e}}_{k}$ of physical interest for light sheet microscopy (fig. S2). The first of these are ${\hat{e}}_{y_{\mathrm{optical}}}$ and ${\hat{e}}_{z_{\mathrm{optical}}}$ defined by the axes of excitation and detection objectives (DOs), since these dictate the optical properties of the light sheet (fig. S2A). The direction ${\hat{e}}_{x_{\mathrm{optical}}}={\hat{e}}_{y_{\mathrm{optical}}}\times {\hat{e}}_{z_{\mathrm{optical}}}$ perpendicular to these offers resolution beyond the support of OTF_det_(***k***) when operating in the structured illumination modes (fig. S2B). Given the shape of OTF_overall_(***k***), the directions ${\hat{e}}_{z_{\max}}$ and ${\hat{e}}_{yz_{\mathrm{diag}}}$ (fig. S2A) are also of special significance, since $k_{z_{\max}}\cdot {\hat{e}}_{z_{\mathrm{optical}}}={\left(R_{z_{\mathrm{optical}}}\right)}_{\max}$determines the highest *z*_optical_ resolution and ∣***k***_*yz*_diag__∣ determines the highest resolution in any direction. Last, when imaging cultured cells, the resolution in the directions ${\hat{e}}_{x_{\mathrm{specimen}}}$ and ${\hat{e}}_{z_{\mathrm{specimen}}}$ parallel and perpendicular to the sample substrate is of particular interest. Since the objectives in LLSM are tilted (fig. S2C) at an angle α in the *xz*_specimen_ plane (α = 32.45° for the LLSM used here), the projections (*R*_*x*_specimen__)_max_ and (*R*_*z*_specimen__)_max_ defined in fig. S2C determine the highest *x*_specimen_ and *z*_specimen_ resolution, respectively. Expressions for all these resolution metrics are derived in note S1C and tabulated for all light sheets studied here in table S1.

### General experimental considerations

#### Practical resolution limits

The theoretical resolution limits of any linear microscope only set an upper bound: The spatial resolution, in practice, also depends on the noise in the image data, the accuracy to which the experimental OTF_overall_(***k***) is known, and the spatial frequency distribution of the specimen (*14*). The interplay between these factors is discussed in depth in note S3A. To measure the practical limits at all spatial frequencies up to the theoretical support, the FT of specimen must itself contain all frequencies. Specimens with sparse, subdiffractive features satisfy this requirement and are widely used in microscopy for resolution characterization. However, the resolution they report may not be achievable in specimens with dense features at the same signal-to-noise ratio (SNR), because the FT of these is heavily weighted near DC, causing high spatial frequencies in the image to fall below the noise floor. Thus, here, we choose to characterize the practical resolution of all light sheets by the more robust metric of the post-deconvolution FT of the image at SNR ~30 of a specimen dense in both real and Fourier space—specifically, the endoplasmic reticulum (ER) of cultured, living LLC-PK1 pig kidney cells, as its thin tubules and complex sheets form a dense and intricate 3D network, particularly in the perinuclear region. For each light sheet, we also estimate the resolution $R({\hat{e}}_{z_{\mathrm{optical}}})$ in real space from a simulated ground truth test pattern at SNR ~20 having narrow stripes of gradually increasing spacing.

In LLSM, spatial resolution is also entwined with the following: the care required during deconvolution for accurate, artifact-free image reconstruction; the degree to which OTF_overall_(***k***) varies as the light sheet propagates; and the axial extent of PSF_exc_(***x***) and amount of energy in excitation sidelobes. These topics are considered further in note S3 (B, C, and D).

#### Light sheet generation

As in (*2*), all light sheets were experimentally generated here by writing an image of the desired light sheet electric field *E*_exc_(*x*, 0, *z*) at the focal point within the specimen onto a specimen-conjugate phase-only spatial light modulator (SLM), and using a pupil-conjugate annular mask of inner and outer diameters NA_min_ and NA_max_ to filter out undiffracted (“DC”) light as well as unwanted higher diffraction orders. The SLM phase Φ_SLM_(*x*, *z*) is given by the real part of *E*_exc_(*x*, 0, *z*), renormalized to a range of ±π, and cropped to eliminate weak sidelobes far from the central excitation maximum

$$E_{\mathrm{norm}}(x,z)=\frac{R\left[E_{\mathrm{exc}}(x,0,z)\right]}{\max\{|R\left[E_{\mathrm{exc}}(x,0,z)\right]|\}}$$ (4a)

$${\text{Φ}}_{\mathrm{SLM}}(x,z)=\pi E_{\mathrm{norm}}(x,z)\;\mathrm{for}|E_{\mathrm{norm}}(x,z)|>\epsilon$$ (4b)

$${\text{Φ}}_{\mathrm{SLM}}(x,z)=0\;\mathrm{for}|E_{\mathrm{norm}}(x,z)|\leq \epsilon$$ (4c)

Typically, a cropping factor ϵ ≲ 0.10 is sufficient to truncate the pattern to the effective width of the light sheet while retaining the vast majority of nonzero pixels within the effective width to achieve high diffraction efficiency. It will be shown that this cropping produces additional axial side bands to the axial excitation bands in the pupil, beneficially helping to fill troughs in OTF_overall_(***k***).

Although we chose an 8-bit grayscale phase SLM here to have fine control over the diffracted pattern, a binary phase SLM was used in (*2*). Therefore, to generate here multi-Bessel (MB) (see the “Multi-Bessel LLSM” section) and axially confined (AC) (see the “Axially confined LLSM” section) LLSs of the type introduced in (*2*), we used our SLM in a binary mode

$$\begin{array}{rl} {\text{Φ}}_{\mathrm{SLM}}^{\mathrm{MB},\mathrm{AC}}(x,z) & =\left(\frac{\pi }{2}\right)E_{\mathrm{norm}}(x,z)/|E_{\mathrm{norm}}(x,z)|+\frac{\pi }{2}\;\\ & \;\;\;\mathrm{for}\;|E_{\mathrm{norm}}(x,z)|>\epsilon \end{array}$$ (4d)

$${\text{Φ}}_{\mathrm{SLM}}^{\mathrm{MB},\mathrm{AC}}(x,z)=0\;\mathrm{for}\;|E_{\mathrm{norm}}(x,z)|\leq \epsilon$$ (4e)

All other light sheets here were generated using the grayscale patterns of Eqs. 4b and 4c.

### Gaussian beam light sheet microscopy

We first apply the above considerations to Gaussian beam light sheet microscopy, as this was the first and remains the simplest and most common form of light sheet microscopy. It also served as the standard against which LLSs were putatively compared in (*10*–*12*, *15*). Analytical expressions for the pupil field $E_{\mathrm{pupil}}^{\mathrm{Gauss}}(k_{x},k_{z})$ and the swept excitation functions ${\mathrm{PSF}}_{\mathrm{exc}}^{\mathrm{sGauss}}(x,0,z)$ and ${\mathrm{OTF}}_{\mathrm{exc}}^{\mathrm{sGauss}}(k_{x},0,k_{z})$ at the *y* = 0 excitation focal plane are derived in note S4. Applying eq. S3 to these then gives the swept overall equivalents, ${\mathrm{OTF}}_{\mathrm{overall}}^{\mathrm{sGauss}}(k_{x},0,k_{z})$ and ${\mathrm{PSF}}_{\mathrm{overall}}^{\mathrm{sGauss}}(x,0,z)$. For other points *y* ≠ 0 along the propagation axis, these parameters are calculated numerically by evaluating the integral in Eq. 1 for $E_{\mathrm{exc}}^{\mathrm{Gauss}}(x)$ using $E_{\mathrm{pupil}}^{\mathrm{Gauss}}(k_{x},k_{z})$ from eq. S12, and applying this to Eqs. 2 and 3 and eqs. S2 and S3. This includes ${\mathrm{PSF}}_{\mathrm{exc}}^{\mathrm{sGauss}}(y,z)$, which shows the divergence of the Gaussian sheet with increasing distance from the excitation focus.

Note from eq. S15b that the excitation PSF for a swept 2D Gaussian beam is identical to that of a 1D Gaussian light sheet, and hence, their overall PSF and OTF are identical. The peak intensity is far lower for the 1D sheet, which may be important for phototoxicity reduction, but the swept beam has the advantage that it can be synchronized with the rolling shutter of certain cameras to filter out diffuse or scattered light in thick specimens. Given this equivalence, the properties of either light sheet could, in principle, be measured experimentally by writing $E_{\mathrm{exc}}^{\mathrm{sGauss}}(x,0,z)$ associated with eq. S15b to the SLM as described in Eq. 4

$$E_{\mathrm{exc}}^{s1\mathrm{DGauss}}(x,z)=\exp\left[-{\left(\frac{z}{w_{o}}\right)}^{2}\right]$$ (5a)

which then produces a vertical excitation line in the pupil given by

$$E_{\mathrm{pupil}}^{s1\mathrm{DGauss}}(k_{x},k_{z})=\delta (k_{x})\exp\left[-\frac{1}{4}{\left(k_{z}w_{o}\right)}^{2}\right]$$ (5b)

However, the annular mask needed to block the undiffracted DC light at the pupil then also blocks the portion of this line for which ∣*k_z_*∣ < *k_o_*NA_min_. This can be avoided by displacing the excitation laterally a distance $x_{p}^{\mathrm{offset}}>{\mathrm{NA}}_{\min}F$ in the pupil so that it is no longer clipped by the annulus. This yields a light sheet of the desired *z* profile, except propagating at an angle $\arcsin[x_{p}^{\mathrm{offset}}/(nF)]$ with respect to the propagation axis y. To create a light sheet of similar properties except propagating along *y*, an identical excitation line can be placed at $-x_{p}^{\mathrm{offset}}$ in the pupil (fig. S3C). The two lines then create a stationary light sheet in the specimen consisting of a standing wave (SW) in *x* bound in *z* by the desired Gaussian envelope (fig. S3E). Sweeping this pattern during image acquisition creates the desired Gaussian light sheet effectively uniform in *x* (fig. S3G, orange curve). The illumination lines themselves are created by diffraction from the SLM when it displays an image *E*_exc_(*x*, 0, *z*) of the stationary Gaussian bound SW pattern (fig. S3B).

Applying this strategy experimentally, we find good agreement with theory for the pupil intensity (fig. S3, C and D), the stationary excitation (fig. S3E) and swept overall PSFs (fig. S3H) at the focal plane, and the overall OTF at both the focal plane (fig. S3, I and J) and near the half width at half maximum (HWHM) of the light sheet (fig. S3, K and L). Fourier shell correlation (FSC) (note S3B) on a simulated image (fig. S3M, bottom) of a stripe pattern of variable pitch (fig. S3M, top) reveals that even the line pair of greatest separation (881 nm, red arrows) is not well resolved after FSC-indicated optimal 10 iterations of Richardson-Lucy (RL) deconvolution (fig. S3, N and O). On an image volume of live LLC-PK1 cells expressing an ER marker (fig. S3P), FSC indicates an optimum of 35 RL iterations at SNR ∼ 30 (fig. S3Q and movie S1, part 2), at which point the fast FT (FFT) (fig. S3Q, upper right inset) of the deconvolved image volume (movie S1, part 3) indicates the ability to detect nearly all spatial frequencies within the support of ${\mathrm{OTF}}_{\mathrm{overall}}^{\mathrm{sGauss}}(k)$ (fig. S3I).

### Sinc beam light sheet microscopy

In (*10*) and (*11*), the putative Gaussian light sheets used for experimental comparison to LLSs were created by cropping a broadly extended line of illumination along *z* with a slit or annulus to create a line of essentially uniform intensity in the pupil plane

$$E_{\mathrm{pupil}}^{\mathrm{sinc}}(k_{x},k_{z})=E_{o}\;\delta (k_{x})\mathrm{rect}[k_{z}/(2k_{o}{\mathrm{NA}}_{\mathrm{sinc}})]$$ (6a)

Because of the line illumination, the stationary PSF ${\mathrm{PSF}}_{\mathrm{exc}}^{\mathrm{sinc}}(x,0,z)$ and the cross-sectional swept PSF ${\mathrm{PSF}}_{\mathrm{exc}}^{\mathrm{ssinc}}(0,z)$ are identical. By Eqs. 1d and 2a, at the focal plane (*y* = 0), they are given by

$${\mathrm{PSF}}_{\mathrm{exc}}^{\mathrm{sinc}}(x,0,z)={\mathrm{PSF}}_{\mathrm{exc}}^{\mathrm{ssinc}}(0,z)\propto {\mathrm{sinc}}^{2}(k_{o}{\mathrm{NA}}_{\mathrm{sinc}}z)$$ (6b)

Thus, the sheets used for comparison in (*10*) and (*11*) are not Gaussian, but rather exhibit a sinc(*z*) cross section in their electric field at focus. We therefore term these sinc light sheets.

As with the Gaussian light sheet, an SLM-generated sinc sheet requires an annular mask to block undiffracted light, which, when the illumination is centered in the pupil, also blocks the portion of $E_{\mathrm{pupil}}^{\mathrm{sinc}}(k_{x},k_{z})$ for which ∣*k_z_*∣ < *k_o_*NA_min_. Experimentally, the solution is the same: Two equal but oppositely offset vertical beamlets of rect(*z*) profile are used to create a pupil field (fig. S4C)

$$E_{\mathrm{pupil}}^{\mathrm{sinc}\mathrm{SW}}(k_{x},k_{z})=E_{o}\;[\delta (k_{x}-k_{o}{\mathrm{NA}}_{\mathrm{exc}})+\delta (k_{x}+k_{o}{\mathrm{NA}}_{\mathrm{exc}})]\mathrm{rect}[k_{z}/(2k_{o}{\mathrm{NA}}_{\mathrm{sinc}})]$$ (6c)

that creates a stationary SW light sheet in *x* bound in *z* by Eq. 6b (fig. S4E). The corresponding swept sheet then also conforms to the desired sinc^2^(*z*) profile but is otherwise uniform in *x* (fig. S4G, orange curve). Applying eq. S1b to Eq. 6b then gives

$${\mathrm{OTF}}_{\mathrm{exc}}^{\mathrm{ssinc}}(0,k_{z})\propto \mathrm{FT}\{{\mathrm{PSF}}_{\mathrm{exc}}^{\mathrm{sinc}}(x,0,z)\}=\mathrm{tri}[k_{z}/(2k_{o}{\mathrm{NA}}_{\mathrm{sinc}})]$$ (7)

at the focus, where tri(*x*) = 1 − ∣*x*∣ for ∣*x*∣ < 1, 0 otherwise. Equation S3 and Eqs. 6b and 7 then give ${\mathrm{PSF}}_{\mathrm{overall}}^{\mathrm{ssinc}}(x,0,z)$ (fig. S4, H and G, red curve) and ${\mathrm{OTF}}_{\mathrm{overall}}^{\mathrm{ssinc}}(k_{x},0,k_{z})$ (fig. S4, I and J) at *y* = 0. For points *y* ≠ 0 along the propagation axis (e.g., fig. S4, F, K, and L), the above parameters are calculated by evaluating the integral in Eq. 1 for $E_{\mathrm{exc}}^{\mathrm{sinc}}(x)$ using $E_{\mathrm{pupil}}^{\mathrm{sinc}}(k_{x},k_{z})$ from Eq. 6a, and applying this to Eqs. 2 to 4 and eqs. S2 and S3.

Experimental metrics for a sinc light sheet generated in this manner are in good agreement with theory, including ${\mathrm{PSF}}_{\mathrm{exc}}^{\mathrm{sincSW}}(x,0,z)$ (fig. S4E), ${\mathrm{PSF}}_{\mathrm{exc}}^{\mathrm{ssinc}}(x,0,z)$ (fig. S4H), and ${\mathrm{OTF}}_{\mathrm{overall}}^{\mathrm{ssinc}}(k)$ at both the focal plane (fig. S4, I and J) and near the HWHM of the light sheet (fig. S4, K and L). FSC on the simulated stripe pattern (fig. S4M) reveals a minimum resolvable stripe separation of 881 nm (fig. S4, N and O, rightmost panels, and movie S2, part 1). On ER-labeled live LLC-PK1 cells (fig. S4P), an optimum of 50 iterations is found (fig. S4Q and movie S2, part 2), which leads to a uniform post-deconvolved FFT within the support of ${\mathrm{OTF}}_{\mathrm{overall}}^{\mathrm{ssinc}}(k)$ (fig. S4I).

The Gaussian and sinc light sheets of figs. S3 and S4 share a comparable propagation length (figs. S3F and S4F). However, they differ in other respects, because the Gaussian excitation profile in the pupil overweights low *k_z_* and underweights high *k_z_* compared to the flat pupil profile of the sinc light sheet. As a result, at the focal plane, OTF_overall_(***k***) is stronger within its *k_z_* support (purple arrows, figs. S4J versus S3J) in the sinc case, yielding improved resolvability of the stripe pattern (figs. S4, N and O, versus S3, N and O) and a stronger recovery of sample spatial frequencies in the ${\hat{e}}_{z}$ direction (yellow arrows, upper right inset, fig. S4Q versus fig. S3Q). Furthermore, the sinc light sheet diverges less rapidly within its propagation range so that its OTF_overall_(*k_z_*) is ∼10× stronger near the support at ∣*y*∣~*y*_HWHM_ than in the Gaussian case (purple arrows, fig. S4L versus fig. S3L). Thus, sinc light sheets are generally preferred to Gaussian ones. We compare both to LLSs below.

### Bessel beam light sheet microscopy

LLSM arose out of earlier work using a swept Bessel beam to create a light sheet much thinner and longer than a conventional Gaussian one (*16*–*20*). An infinitesimally thin ring of illumination at the pupil plane of an objective creates a theoretically ideal Bessel beam that is infinitely long, with a narrow central peak surrounded by an infinite series of concentric sidelobes. To create an axially long but radially confined beam better suited to light sheet microscopy, a ring of finite width from NA_min_ to NA_max_ is used to create a constant annular electric field

$$E_{\mathrm{pupil}}^{\mathrm{BB}}(k_{x},k_{z})=E_{o}\mathrm{rect}\left(\frac{{\mathrm{NA}}_{\rho }-{\mathrm{NA}}_{\mathrm{mid}}}{{\mathrm{NA}}_{\mathrm{range}}}\right)=E_{o}\mathrm{rect}\left(\frac{k_{\rho }-k_{\rho }^{\mathrm{mid}}}{k_{\rho }^{\mathrm{range}}}\right)$$ (8a)

where $k_{\rho }=\sqrt{k_{x}^{2}+k_{z}^{2}}={\mathrm{NA}}_{\rho }k_{o}$, $k_{\rho }^{\max}={\mathrm{NA}}_{\max}k_{o},k_{\rho }^{\min}={\mathrm{NA}}_{\min}k_{o}$

$$\begin{array}{r} k_{\rho }^{\mathrm{mid}}=\frac{k_{\rho }^{\max}+k_{\rho }^{\min}}{2}={\mathrm{NA}}_{\mathrm{mid}}k_{o},\\ k_{\rho }^{\mathrm{range}}=k_{\rho }^{\max}-k_{\rho }^{\min}={\mathrm{NA}}_{\mathrm{range}}k_{o} \end{array}$$ (8b)

By Eq. 1a, the integral representation of the Bessel function *J*_0_(*x*), and the identity ∫*x^ν^J*_ν−1_(*x*)*dx* = *x^ν^J_ν_*(*x*), the electric field at the focal plane is then

$$E_{\mathrm{exc}}^{\mathrm{BB}}(\rho ,0)=E_{o}\left[\frac{J_{1}(k_{\rho }^{\max}\rho )}{k_{\rho }^{\max}\rho }-\frac{J_{1}(k_{\rho }^{\min}\rho )}{k_{\rho }^{\min}\rho }\right]$$ (8c)

All four ways in which a confined beam can be moved to create a light sheet have been applied to Bessel beams. The confocal mode (note S1B) (*20*, *21*) efficiently removes sidelobe emission from the detected signal and extends the theoretical support along ${\hat{e}}_{x_{\mathrm{optical}}}$ to the sum of the excitation and detection supports (eq. S10g). However, its practical resolution is constrained by the weakness of ${\mathrm{OTF}}_{\mathrm{overall}}^{\mathrm{conf}}(k)$ in the region of extended resolution. Similarly, the incoherent structured light sheet mode (note S1C) (*22*, *23*) is compromised by the weakness of the nonzero incoherent harmonics. Furthermore, the serial beam stepping common to both these modes slows acquisition and requires power high enough to lead to premature phototoxicity. Thus, we focus here on the swept (note S1A) (*16*–*18*) and coherent MB (note S1D) (*22*, *24*) modes.

#### Swept Bessel beam light sheet microscopy

The annular pupil field of Eq. 8a and fig. S5B for a radially bound Bessel-like beam results in a stationary ${\mathrm{PSF}}_{\mathrm{exc}}^{\mathrm{BB}}(x,0,z)$ (fig. S5C) at the focus given by ${|E_{\mathrm{exc}}^{\mathrm{BB}}(\rho ,0)|}^{2}$ from Eq. 8c. The corresponding ${\mathrm{OTF}}_{\mathrm{exc}}^{\mathrm{BB}}(k)$ (fig. S5D) of the Bessel beam is nonzero throughout its *k_z_* support and has a secondary maximum there. By eq. S1b, so does the axial swept ${\mathrm{OTF}}_{\mathrm{exc}}^{\mathrm{sBB}}(y,k_{z})={\mathrm{OTF}}_{\mathrm{exc}}^{\mathrm{BB}}(0,y,k_{z})$ (fig. S5F). Consequently, by eq. S3b, the swept ${\mathrm{OTF}}_{\mathrm{overall}}^{\mathrm{sBB}}(k_{x},y,k_{z})$ (fig. S5, I and J) is far stronger near its *k_z_* support than is either ${\mathrm{OTF}}_{\mathrm{overall}}^{\mathrm{sGauss}}(k_{x},y,k_{z})$ or ${\mathrm{OTF}}_{\mathrm{overall}}^{\mathrm{ssinc}}(k_{x},y,k_{z})$.

#### Coherent MB light sheet microscopy

To overcome the speed limitations associated with a single stepped or swept beam, a diffractive optical element was used in (*23*) to create a linear array of *N* = 7 parallel bound Bessel beams, each of which then needed to step over only 1/*N*th of the desired FOV. In another application, the peak power was reduced sevenfold by keeping the volume acquisition speed and FOV the same as for a single beam, while the excitation intensity was reduced sevenfold and the camera integration time per plane increased by the same amount. Unexpectedly, despite the same integrated exposure, it was found that this multibeam, low-power mode was considerably less phototoxic to live cells, while it simultaneously preserved the specimen fluorescence for more recorded image volumes over the same FOV.

In these experiments, to ensure that the bound Bessel beams did not coherently interfere with one another, their mutual separation was chosen to be larger than the envelope of their respective sidelobes. However, given the observed benefits of improved speed and/or reduced phototoxicity and bleaching, there was an incentive to investigate massively parallel 1D MB beam arrays in the limit of even smaller period T where the beams do coherently interfere

$$E_{\mathrm{exc}}^{\mathrm{cMB}}(x)=\sum_{j=-\infty }^{\infty }E_{\mathrm{exc}}^{\mathrm{BB}}(\rho_{j},y)$$ (9a)

where $\rho_{j}=\sqrt{{\left(x-jT\right)}^{2}+z^{2}}$. This is the limit of note S1D. Hence, by Eq. 8a, the pupil field $E_{\mathrm{pupil}}^{\mathrm{cBB}}(k_{x},k_{z})$ giving rise to $E_{\mathrm{exc}}^{\mathrm{cMB}}(x,z)$ is

$$E_{\mathrm{pupil}}^{\mathrm{cMB}}(k_{x},k_{z})=E_{o}\sum_{m=-M}^{M}\mathrm{rect}\left(\frac{\sqrt{{\left(2\pi m/T\right)}^{2}+k_{z}^{2}}-k_{\rho }^{\mathrm{mid}}}{k_{\rho }^{\mathrm{range}}}\right)\equiv \sum_{m=-M}^{M}E_{\mathrm{band}}(m)$$ (9b)

where *M* is the largest integer for which *M* < NA_exc_T/λ_exc_ and $k_{\rho }^{\mathrm{mid}}$ and $k_{\rho }^{\mathrm{range}}$ are defined in Eq. 8b. In other words, the pupil field for a coherent periodic array of Bessel beams is given by series of 2*M* + 1 lines of period *k_x_* = 2π/T and uniform amplitude along the *z_p_* axis, cropped by the annulus that defines the single bound Bessel beam.

To create a coherent MB pattern in (*2*), the desired field in Eq. 9a was applied to Eqs. 4a, 4d, and 4e) to write a binary phase pattern on an SLM (fig. S6A). After passing through a transform lens and an annular mask, diffraction from this pattern produces a pupil field having the form of Eq. 9b, except with bands of variable rather than uniform intensity (fig. S6B). The relative intensities of these bands are controlled through the cropping factor ϵ of Eqs. 4d and 4e, with smaller cropping factors producing pupil bands $E_{\mathrm{band}}^{\mathrm{SLM}}(m)$ that are stronger at higher *k_z_*, at the expense of a broader excitation envelope in *z*. By Eqs. 1d and 2, this field produces a stationary ${\mathrm{PSF}}_{\mathrm{exc}}^{\mathrm{cMB}}(x,0,z)$ (fig. S6C) and a corresponding ${\mathrm{OTF}}_{\mathrm{exc}}^{\mathrm{cMB}}(k_{x},0,k_{z})$ (fig. S6D) at the focal plane given by

$${\mathrm{OTF}}_{\mathrm{exc}}^{\mathrm{cMB}}(k_{x},0,k_{z})\propto \sum_{m=-M}^{M}\sum_{m^{\prime }=-M}^{M}E_{\mathrm{band}}^{\mathrm{SLM}}(m){⊗}_{k_{x}k_{z}}\bar{E_{\mathrm{band}}^{\mathrm{SLM}}(m^{\prime })}$$ (10a)

Because ${\mathrm{PSF}}_{\mathrm{exc}}^{\mathrm{cMB}}(x)$ extends across the entire FOV, fluorescent molecules across the image radiate simultaneously, greatly reducing the acquisition time and peak power needed to image a complete image plane. Because ${\mathrm{PSF}}_{\mathrm{exc}}^{\mathrm{cMB}}(x)$ is periodic, it can be used in either the swept (note S1A) or coherent structured illumination modes (note S1D). For the latter, acquisition of 4*M* + 1 raw images with ${\mathrm{PSF}}_{\mathrm{exc}}^{\mathrm{cMB}}(x)$ phase stepped in increments of Δ*x* = T/(4*M* + 1) produces a reconstructed image with resolution extended along ${\hat{e}}_{x_{\mathrm{optical}}}^{\mathrm{SI}}$ (eq. S10h and fig. S2B), as seen at the focal plane in ${\mathrm{OTF}}_{\mathrm{overall}}^{\mathrm{cSIMB}}(k_{x},0,k_{z})$ (fig. S6, K and L). For the swept mode, Eq. 10a and eq. S1b give (fig. S4F)

$${\mathrm{OTF}}_{\mathrm{exc}}^{\mathrm{scMB}}(0,k_{z})={\mathrm{OTF}}_{\mathrm{exc}}^{\mathrm{cMB}}(0,0,k_{z})\propto \sum_{m=-M}^{M}E_{\mathrm{band}}^{\mathrm{SLM}}(m){⊗}_{k_{x}k_{z}}\bar{E_{\mathrm{band}}^{\mathrm{SLM}}(m)}$$ (10b)

Using Eq. 10b and ${\mathrm{PSF}}_{\mathrm{exc}}^{\mathrm{scMB}}(0,z)={\mathrm{FT}}_{k_{z}}^{-1}\{{\mathrm{OTF}}_{\mathrm{exc}}^{\mathrm{scMB}}(0,k_{z})\}$, the convolution theorem gives

$${\mathrm{PSF}}_{\mathrm{exc}}^{\mathrm{scMB}}(0,z)=\sum_{m=-M}^{M}{\mathrm{FT}}_{k_{z}}^{-1}\{E_{\mathrm{band}}^{\mathrm{SLM}}(m)\}\cdot {\mathrm{FT}}_{k_{z}}^{-1}\{\bar{E_{\mathrm{band}}^{\mathrm{SLM}}(m)}\}=\sum_{m=-M}^{M}{\mathrm{PSF}}_{\mathrm{band}}^{\mathrm{SLM}}(m)$$ (11)

Equation 11 represents the field synthesis theorem (*15*): The swept sheet excitation PSF (fig. S6H, green curve) is the incoherent sum of the excitation PSFs formed by each of the individual bands of fixed *k_x_* in the pupil. Equation 9 and eqs. S3 and S8b then give ${\mathrm{PSF}}_{\mathrm{overall}}^{\mathrm{scMB}}(x,0,z)$ (fig. S6, G and H, red curve) and ${\mathrm{OTF}}_{\mathrm{overall}}^{\mathrm{scMB}}(k_{x},0,k_{z})$ (fig. S6, I and J).

### Lattice light sheet microscopy

In the course of exploring the effect of the period T on the properties of coherent MB light sheets [movie S18 of (*2*)], it was found that there exist specific periods where the excitation maxima of the light sheet exhibit the symmetry of a 2D optical lattice [figure S27 of (*2*)]. An ideal 2D optical lattice forms a periodic pattern across *xz* space and propagates without change in *y*. These lattices are defined by the symmetry operations (e.g., rotation, translation, and reflection) that map the lattice onto itself. Each 2D lattice is composed of a minimum of *N* = 3 mutually interfering plane waves. Maximally symmetric composite lattices (*2*, *25*, *26*) with *N* > 3 plane waves are formed by adding additional plane waves whose wave vectors are found by applying all valid symmetry operations to the wave vectors of the initial plane wave set. These lattices provide the most tightly confined intensity maxima with the greatest contrast relative to the surrounding background for a given symmetry. They are therefore particularly well suited to serve as the starting point for either swept or coherent structured illumination light sheet microscopy where the ideal lattice is bound in *z* by replacing its discrete illumination points in the rear pupil with stripes of finite extent in *z* [movie S1 of (*2*)].

#### Considerations in choosing a lattice of a given symmetry

The field of any ideal 2D optical lattice composed of *N* plane waves can be expressed as

$$E_{\mathrm{exc}}^{\mathrm{lattice}}(x,t)=\exp[i(k_{y}y+\omega t)]\sum_{n=1}^{N}E_{n}\exp[i{\left(k_{x}\right)}_{n}x+i{\left(k_{z}\right)}_{n}z]$$ (12a)

where *k_y_* = *k*cosθ = *k*NA_exc_/*n*. These produce a longitudinally invariant excitation PSF in the specimen given by

$${\mathrm{PSF}}_{\mathrm{exc}}^{\mathrm{lattice}}(x)={\mathrm{PSF}}_{\mathrm{exc}}^{\mathrm{lattice}}(x,z)=E_{\mathrm{exc}}^{\mathrm{lattice}}(x,t)\cdot \bar{E_{\mathrm{exc}}^{\mathrm{lattice}}(x,t)}=\sum_{n=1}^{N}{|E_{n}|}^{2}+\sum_{n=1}^{N}\sum_{n^{\prime }\neq n}^{N}E_{n}\cdot \bar{E_{n^{\prime }}}\exp\{i[{\left(k_{x}\right)}_{n}-{\left(k_{x}\right)}_{n^{\prime }}]x+i[{\left(k_{z}\right)}_{n}-{\left(k_{z}\right)}_{n^{\prime }}]z\}$$ (12b)

The DC portion of the corresponding ${\mathrm{OTF}}_{\mathrm{exc}}^{\mathrm{lattice}}(k_{x},k_{z})$ is encompassed by the first sum in Eq. 12b, whereas each term in the double sum corresponds to a nonzero spatial frequency ***k***_***m***_ **−** ***k***_***m***′_. As *N* increases, the DC term becomes increasingly dominant over the nonzero frequencies that are responsible for resolution extension in ${\mathrm{OTF}}_{\mathrm{overall}}^{\mathrm{lattice}}(k)$ beyond the widefield OTF. Thus, to maximize the relative strength of these higher spatial frequencies and enable robust recovery of sample information in the presence of noise out to the extended axial support that they produce, one should start with a lattice having the fewest number of plane waves necessary to cover ${\mathrm{OTF}}_{\mathrm{exc}}^{\mathrm{lattice}}(k_{x},k_{z})$ within the entirety of the desired *k_x_k_z_* support without introducing undesirable consequences, such as artifacts in image reconstruction or premature photobleaching/photodamage from excessive out-of-focus excitation.

##### 1D axial SW

**Fig. 1.**
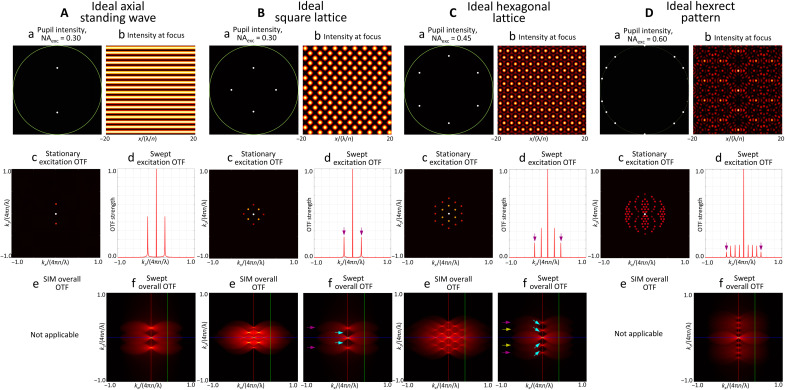
Characteristics of four ideal optical lattices of different symmetries useful for LLSM. (**A**) An ideal axial standing wave, (**B**) an ideal square optical lattice, (**C**) an ideal hexagonal lattice, and (**D**) a spatially structured aperiodic pattern formed by the coherent interference of a hexagonal lattice with two rectangular lattices of different periods. Each successive lattice, as seen in the cross section at the specimen [subpanel b in (A) to (D)], is created by adding illumination points in the objective rear pupil (subpanel a) to the preceding one at positions on the ring of constant NA_exc_ in the pupil. These points are chosen to create new discrete spatial frequencies in the swept sheet axial excitation transfer function halfway between existing ones (subpanel d). These frequencies generate additional copies of the detection transfer function OTF_det_ in the overall transfer function exactly positioned to fill the gaps (light blue arrows, subpanel f) in the preceding one. However, as the number of pupil illumination points increases, the axially shifted copies of OTF_det_ in that give rise to extended resolution weaken (e.g., purple arrows, subpanels d and f), making recovery of the sample information they encode more difficult. Thus, they should be added only as needed when the desired NA_exc_ increases to the point that the OTF gaps become difficult to fill via RL deconvolution. Alternatively, the lattice pattern can be laterally stepped rather than swept, and the resulting set of raw images was processed by structured illumination (*13*), creating a transfer function (subpanel e) of laterally extended resolution and fully filled OTF gaps, at the cost of slower imaging speed and faster photobleaching.

The smallest plane wave set that provides the greatest resolution extension in *z* for a given NA_exc_ is created by a pupil field consisting of a pair of points at ±*k_o_*NA_exc_ on the $k_{z}^{\mathrm{pupil}}$ axis (Fig. 1Aa). This creates an axial (*z* axis) SW ${\mathrm{PSF}}_{\mathrm{exc}}^{z\mathrm{SW}}(z)$ in the specimen (Fig. 1Ab) having a swept excitation OTF (Fig. 1Ad) consisting of harmonics at $k_{z}^{\mathrm{OTF}}=\pm 2k_{o}{\mathrm{NA}}_{\mathrm{exc}}$ half as strong as the DC peak they straddle. Expressions for the OTFs associated with this pattern are derived in note S5A.

The axial SW light sheet is identical to a coherent MB light sheet (see the “Coherent MB light sheet microscopy” section) of period T smaller than the diffraction limit (T < λ_exc_/NA_exc_). In this limit, only the two polar stripes in the *m* = 0 band of $E_{\mathrm{pupil}}^{\mathrm{cMB}}(k_{x},k_{z})$ in eq. S8b remain.

##### 2D maximally symmetric square lattice

For all lattices, the DC region of ${\mathrm{OTF}}_{\mathrm{overall}}^{\mathrm{lattice}}(k)$ is automatically covered by the first sum in Eq. 12b and only gets stronger relative to the regions beyond OTF_det_(***k***) as more plane waves are added. Thus, usually, it is unnecessary and even counterproductive to craft light sheets having pupil excitation near the *k_z_* = 0 equatorial line. A useful exception is light sheets based on a maximally symmetric square lattice (Fig. 1Bb), which has a pupil field consisting of pairs of points at ±*k_o_*NA_exc_ on both the $k_{x}^{\mathrm{pupil}}$ and $k_{z}^{\mathrm{pupil}}$ axes (Fig. 1Ba).

In the coherent SIM mode, the two additional illumination points on the $k_{x}^{\mathrm{pupil}}$ axis extend the support of ${\mathrm{OTF}}_{\mathrm{overall}}^{\mathrm{cSISq}}(k)$ (Fig. 1Be) by the same amount in $k_{x}^{\mathrm{OTF}}$ as do the two points on the $k_{z}^{\mathrm{pupil}}$ axis common to both the square and axial SW lattices. Expressions for the OTFs associated with this pattern are derived in note S5B. In particular, ${\mathrm{OTF}}_{\mathrm{exc}}^{\mathrm{Sq}}(k_{x},k_{z})$ (eq. S17B) has four cross terms at $\delta (k_{z}^{\mathrm{OTF}}\pm k_{o}{\mathrm{NA}}_{\mathrm{exc}})\;\delta (k_{x}^{\mathrm{OTF}}\pm k_{o}{\mathrm{NA}}_{\mathrm{exc}})/2$ (Fig. 1, B and C) that, in the SIM mode, fill in (Fig. 1Be) the gaps (light blue arrows in Fig. 1Bf) seen in ${\mathrm{OTF}}_{\mathrm{overall}}^{\mathrm{sSq}}(k)$ of the swept mode. In addition, as all illumination points in the pupil are extended as lines in $k_{z}^{\mathrm{pupil}}$ to produce an LLS, the two equatorial points can be extended the furthest while still remaining within the annulus that dictates the light sheet propagation length. This improves the light sheet confinement and reduces the size and depth of the troughs/gaps ${\mathrm{OTF}}_{\mathrm{overall}}^{\mathrm{sSq}}(k)$. However, these advantages come at the cost of a further twofold diminishment of the strength of the $k_{z}^{\mathrm{OTF}}=\pm 2k_{o}{\mathrm{NA}}_{\mathrm{exc}}$ shifted copies of OTF_det_(***k***) in ${\mathrm{OTF}}_{\mathrm{overall}}^{\mathrm{sSq}}(k)$ and ${\mathrm{OTF}}_{\mathrm{overall}}^{\mathrm{cSISq}}(k)$ relative to those in the axial SW.

A square light sheet derived from the lattice described here is identical to a coherent MB light sheet of period T = λ_exc_/NA_exc_. This leaves only the two polar stripes in the *m* = 0 band and the two equatorial stripes of the *m* = ± 1 bands of $E_{\mathrm{pupil}}^{\mathrm{cMB}}(k_{x},k_{z})$ in eq. S8b.

##### 2D maximally symmetric hexagonal lattice

For either the axial SW or the swept mode of the maximally symmetric square lattice, as NA_exc_ is increased to increase the axial support (*R*_*z*_optical__)_max_ of eq. S10c, the gap between the DC copy and the $k_{z}^{\mathrm{OTF}}=\pm 2k_{o}{\mathrm{NA}}_{\mathrm{exc}}$ shifted copies of OTF_det_(***k***) in OTF_overall_(***k***) increases, until eventually OTF_overall_(***k***) becomes discontinuous. This occurs when the shift is larger than the maximum width of the “bowtie” region of OTF_det_(***k***) or, using eq. S10c, when

$${\mathrm{NA}}_{\mathrm{exc}}>\frac{\lambda_{\mathrm{exc}}}{\lambda_{\det}}(n-\sqrt{n^{2}-{\mathrm{NA}}_{\det}^{2}})$$ (13)

The consequences of gaps or even complete discontinuities in OTF_overall_(***k***) will be explored below. However, one solution for the axial SW or swept square lattice is to add illumination points in the rear pupil to create additional shifted copies of OTF_det_(***k***) at the exact centers $k_{z}^{\mathrm{OTF}}=\pm k_{o}{\mathrm{NA}}_{\mathrm{exc}}$ of their gaps. This results (eq. S19a) in a pupil pattern consisting of six illumination points equally spaced azimuthally on a ring of radius $k_{\rho }^{\mathrm{pupil}}=k_{o}{\mathrm{NA}}_{\mathrm{exc}}$ (Fig. 1Ca). These are the exact conditions that produce an ideal maximally symmetric lattice of hexagonal symmetry (Fig. 1Cb).

Expressions for the OTFs associated with this pattern are derived in note S5A. In the swept mode, the $k_{z}^{\mathrm{OTF}}=\pm 2k_{o}{\mathrm{NA}}_{\mathrm{exc}}$ peaks in ${\mathrm{OTF}}_{\mathrm{exc}}^{\mathrm{sHex}}(k_{z})$ that provide the highest axial resolution in ${\mathrm{OTF}}_{\mathrm{overall}}^{\mathrm{sHex}}(k)$and the gap-filling peaks at $k_{z}^{\mathrm{OTF}}=\pm 2k_{o}{\mathrm{NA}}_{\mathrm{exc}}$ are one-sixth and one-third the strength of the DC peak, respectively (Fig. 1Cd). However, the 19 peaks in ${\mathrm{OTF}}_{\mathrm{exc}}^{\mathrm{Hex}}(k)$ (Fig. 1Cc) yield a gap-free ${\mathrm{OTF}}_{\mathrm{overall}}^{\mathrm{cSIHex}}(k)$ (Fig. 1Ce) in the SIM mode that is reasonably uniform throughout its support.

A hexagonal light sheet derived from the lattice described here is identical to a coherent MB light sheet of period $T=(2/\sqrt{3})\lambda_{\mathrm{exc}}/{\mathrm{NA}}_{\mathrm{exc}}$. This leaves only the two polar stripes in the *m* = 0 band and two pairs of stripes each from the *m* = ± 1 bands of $E_{\mathrm{pupil}}^{\mathrm{cMB}}(k_{x},k_{z})$ in eq. S8b.

##### 2D hexagonal-rectangular aperiodic pattern

As NA_exc_ increases further, the four small gaps between the DC, $k_{z}^{\mathrm{OTF}}=\pm k_{o}{\mathrm{NA}}_{\mathrm{exc}}$, and $k_{z}^{\mathrm{OTF}}=\pm 2k_{o}{\mathrm{NA}}_{\mathrm{exc}}$ shifted copies of OTF_det_(***k***) in ${\mathrm{OTF}}_{\mathrm{overall}}^{\mathrm{sHex}}(k)$ for the hexagonal lattice become larger. Following the same procedure as above, these gaps can be filled by adding eight more illumination points on the ring of *k*_ρ_ = *k_o_*NA_exc_ in the pupil at $k_{z}^{\mathrm{pupil}}=\pm k_{o}{\mathrm{NA}}_{\mathrm{exc}}/4$ and $k_{z}^{\mathrm{pupil}}=\pm 3k_{o}{\mathrm{NA}}_{\mathrm{exc}}/4$ (Fig. 1Da and note S5D). This produces a complex, aperiodic interference pattern at the specimen focal plane (Fig. 1Db) consisting of 91 discrete spatial frequencies in ${\mathrm{OTF}}_{\mathrm{exc}}^{\mathrm{HexRect}}(k)$ (Fig. 1Dc), which, by its aperiodic nature, cannot be applied to coherent SIM. However, by Eq. 11, if the pattern is swept far enough, then the resulting ${\mathrm{OTF}}_{\mathrm{exc}}^{\mathrm{sHexRect}}(k_{z})$ (Fig. 1Dd) is the incoherent sum of the swept excitation OTFs of the hexagonal lattice above and two rectangular lattices of periods (4/$\sqrt{15})\lambda_{\mathrm{exc}}/{\mathrm{NA}}_{\mathrm{exc}}$ and (4/$\sqrt{7})\lambda_{\mathrm{exc}}/{\mathrm{NA}}_{\mathrm{exc}}$ corresponding to the illumination points at $k_{z}^{\mathrm{pupil}}=\pm k_{o}{\mathrm{NA}}_{\mathrm{exc}}/4$ and $k_{z}^{\mathrm{pupil}}=\pm 3k_{o}{\mathrm{NA}}_{\mathrm{exc}}/4$, respectively. Thus, we describe this as a hexagonal-rectangular (hexrect) aperiodic pattern. ${\mathrm{OTF}}_{\mathrm{overall}}^{\mathrm{sHexRect}}(k)$ (Fig. 1Df) consists of nine copies of OTF_det_(***k***) equally spaced in *k_z_*, which further minimizes the space occupied by gaps. However, because 14 wave vectors are needed to produce the pattern, the DC copy is 14× stronger than the ± 2*k_o_*NA_exc_ copies that give the greatest resolution extension in *z*.

Because the hexrect pattern is aperiodic, it is not related to a coherent MB light sheet. However, from the trends in Fig. 1, it is clear that as more illumination points are added to the pupil, the swept overall OTF becomes increasingly continuous but increasingly also dominated by the DC portion. In particular, the hexrect pattern, with 16 illumination points, approaches the characteristics of a single swept Bessel beam (fig. S5). In addition, as more illumination points are added, the maxima of the resulting coherent pattern become further spaced, requiring higher peak power to image at a given speed. Thus, as NA_exc_ is increased to increase the axial support, the lattice requiring the fewest number of illumination points (i.e., wave vectors) to achieve the desired propagation length while still enabling faithful post-deconvolution image reconstruction should be selected.

##### Multi-Bessel LLSM

Since the axial SW, square, and hexagonal infinite lattices above are examples of the coherent MB light sheets of the “Coherent MB light sheet microscopy” section in the limit where the annulus width approaches zero, they can be used to produce confined light sheets of the same symmetry by replacing each of their pupil illumination points with a stripe of uniform illumination centered on $k_{\rho }^{\mathrm{mid}}=k_{o}{\mathrm{NA}}_{\mathrm{exc}}$ cropped by a finite width annulus of radii $k_{\rho }^{\max}=k_{o}{\mathrm{NA}}_{\max},k_{\rho }^{\min}=k_{o}{\mathrm{NA}}_{\min}$ (Eqs. 8a and 8b). This then recapitulates the MB pupil field $E_{\mathrm{pupil}}^{\mathrm{cMB}}(k_{x},k_{z})$ of Eq. 9b, where the period T is given by

$$T<\lambda_{\mathrm{exc}}/{\mathrm{NA}}_{\mathrm{exc}}\;(\mathrm{axial}\;\mathrm{SW})$$ (14a)

$$T=\lambda_{\mathrm{exc}}/{\mathrm{NA}}_{\mathrm{exc}}\;(\mathrm{square}\;\mathrm{lattice})$$ (14b)

$$T=\left(\frac{2}{\sqrt{3}}\right)\lambda_{\mathrm{exc}}/{\mathrm{NA}}_{\mathrm{exc}}\;(\mathrm{hexagonal}\;\mathrm{lattice})$$ (14c)

A key advantage of MB LLSs lies in the uniformity of their ${\mathrm{OTF}}_{\mathrm{overall}}^{\mathrm{MB}}(k)$ over the desired propagation range ∣*y*∣ ≲ *y*_HWHM_. The individual propagation length (*y*_FWHM_)*_b_* of any beamlet *b* of the *B* beamlets comprising an LLS is given by

$${\left(y_{\mathrm{FWHM}}\right)}_{b}\approx \frac{\pi }{\left\{[{\left(k_{\rho }^{\min}\right)}_{b}-{\left(k_{\rho }^{\max}\right)}_{b}]\cdot {\hat{e}}_{y}\right\}}=\frac{\lambda_{\mathrm{exc}}/n}{2\left\{\sqrt{1-{\left[{\left({\mathrm{NA}}_{\min}\right)}_{b}/n\right]}^{2}}-\sqrt{1-{\left[{\left({\mathrm{NA}}_{\max}\right)}_{b}/n\right]}^{2}}\right\}}$$ (15)

**Fig. 2.**
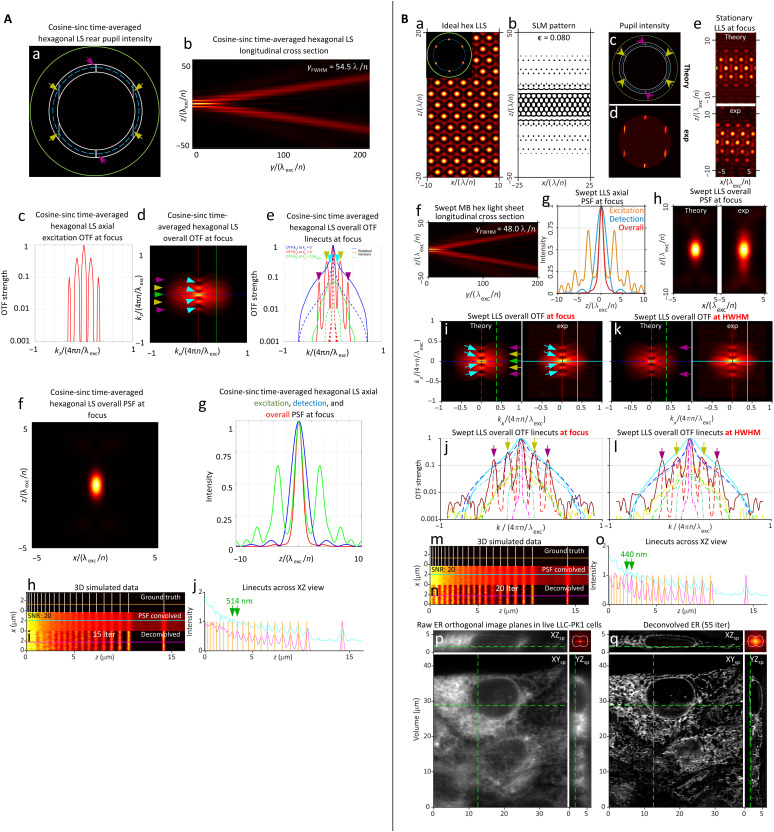
Comparison of methods to generate a swept hexagonal LLS. (**A**) Theoretical characteristics of a cosine-sinc light sheet generated by field synthesis (*15*), designed to mimic an LLS of hexagonal symmetry. NA_exc_ = 0.40, NA_annulus _= 0.435/0.365, *y*_FWHM _= 54.5λ_exc_/*n*. (**B**) Theoretical and experimentally measured characteristics of a swept MB LLS having hexagonal symmetry. NA_exc_= 0.43, NA_annulus_= 0.47/0.40, cropping factor ϵ = 0.080, and *y*_FWHM _= 48.0 λ_exc_/*n*. The MB LLS produces stronger peaks and shallower troughs in ${\mathrm{OTF}}_{\mathrm{overall}}^{\mathrm{swept}}$ (subpanels i to l) compared to subpanels d and e of (A), leading to a higher modulation depth and smaller resolvable spacing [subpanels j of (A) and o of (B)] in simulated images of a line pattern of variable spacing [subpanels h of (A) and m of (B)]. Three dot-dash curves in subpanels j and l of (B) (and the same subpanels for all other light sheet figures) give for reference the strength of OTF_det_ along the *k_x_* (light blue) and *k_z_* (pink) axes, as well as the line *k_x_* = 2πNA_det_/λ_det_, where the widefield microscope has its highest resolution in *z*.

For beamlets that do not cross the equatorial pupil line *z_p_* = 0 (fig. S8A), (NA_min_)*_b_* and (NA_max_)*_b_* are estimated by the NA of the points in the beamlet closest and furthest from the equatorial line at which the intensity drops below a chosen threshold. For beamlets that do cross *z_p_* = 0 (fig. S8B), (NA_min_)*_b_* is given by the NA at the point where the beamlet crosses the line. Since the bands of any coherent MB light sheet span the entirety of the annulus in *k_z_*, all beamlets with (*k_x_*)*_b_* = 2π ∣*m*∣/T < *k_o_*(NA_min_)_annulus_ have identical values of (NA_max_)*_b_* = (NA_max_)_annulus_ and (NA_min_)*_b_* = (NA_min_)_annulus_ (e.g., the polar beamlets in fig. S8C) and hence, by Eq. 15, the same propagation length. This includes, for example, all six beamlets comprising an MB hexagonal LLS (Fig. 2Bc) and explains how the ±*k_o_*NA_exc_ and ±2*k_o_*NA_exc_ shifted copies of OTF_det_(***k***) in ${\mathrm{OTF}}_{\mathrm{overall}}^{\mathrm{sMBHex}}(k)$ maintain their relative amplitudes from *y* = 0 to *y* = *y*_HWHM_ (Fig. 2, Bi and Bk, and gold and purple arrows, Fig. 2, Bj and Bl). On the other hand, MB beamlets with (*k_x_*)*_b_* > *k_o_*(NA_min_)_annulus_ have longer propagation lengths. This includes the two equatorial beamlets of the square lattice, which only match (*y*_FWHM_)*_b_* of the polar beamlets when they are tangent to (NA_min_)_annulus_ (fig. S8C).

We consider two different means that have been used to produce MB light sheets experimentally. In the first, by Eq. 1d, the electric field $E_{\mathrm{exc}}^{\mathrm{cMB}}(x,0,z)$ at the focal point within the sample is determined from the inverse FT of $E_{\mathrm{pupil}}^{\mathrm{cMB}}(k_{x},k_{z})$ as given by Eqs. 9b and 14. The normalized real part of $E_{\mathrm{exc}}^{\mathrm{cMB}}(x,0,z)$ is then applied to a sample-conjugate SLM according to Eq. 4. The light diffracted by this pattern is passed through a pupil conjugate annular mask and then focused by an excitation objective (EO) to create the light sheet within the sample. In (*2*) and the examples here, the SLM is used in a binary mode, so Eqs. 4d and 4e apply.

The second approach applies the result of the field synthesis theorem in Eq. 11 that ${\mathrm{PSF}}_{\mathrm{exc}}^{\mathrm{scMB}}(0,z)$ of a swept MB light sheet is the incoherent sum of the excitation PSFs formed by each of the individual bands of fixed *k_x_* in the pupil. Thus, in (*15*), time-averaged versions of swept LLSs were generated by discretely and serially stepping a line of illumination oriented in the *k_z_* direction to the 2*M* + 1 positions of these bands (fig. S7). In this case (note S6), the PSFs of the individual bands in Eq. 11 are of the form (eq. S22)

$${\mathrm{PSF}}_{\mathrm{band}}(m,z)\propto \cos^{2}[{\left(k_{z}^{\mathrm{mid}}\right)}_{m}z]{\mathrm{sinc}}^{2}[{\left(k_{z}^{\mathrm{range}}\right)}_{m}z/2]\;\mathrm{where}$$ (16a)

$${\left(k_{z}^{\mathrm{mid}}\right)}_{m}=[{\left(k_{z}^{+}\right)}_{m}+{\left(k_{z}^{-}\right)}_{m}]/2,{\left(k_{z}^{\mathrm{range}}\right)}_{m}={\left(k_{z}^{+}\right)}_{m}-{\left(k_{z}^{-}\right)}_{m}$$ (16b)

We therefore term patterns created by this method cosine-sinc (CS) light sheets. Both the cos^2^ and sinc^2^ terms contribute to the axial resolution. In the limit ${\left(k_{z}^{\mathrm{range}}\right)}_{m}\to 0$ (i.e., NA_max_ − NA_min_ → 0), the sinc^2^ function binds PSF_band_(*m*, *z*) increasingly weakly so that the cos^2^ term dominates. This strengthens the axial ${\mathrm{OTF}}_{\mathrm{exc}}^{\mathrm{CS}}(k_{z})$ near the $\pm 2{\left(k_{z}^{\mathrm{mid}}\right)}_{m}$ shifted copies of OTF_det_(***k***) at the expense of stronger sidelobes in ${\mathrm{PSF}}_{\mathrm{exc}}^{\mathrm{CS}}(z)$.

Although the two approaches can produce similar results in the swept mode (Fig. 2 and fig. S9), only the SLM approach produces a light sheet structured in *x* that can be used in the coherent SIM mode to fill all gaps in ${\mathrm{OTF}}_{\mathrm{overall}}^{\mathrm{MB}}(k)$ (e.g., Fig. 1, Be and Ce) and extend the *k_x_* support to the limit of Eq. S10h. In addition, SLM-generated light sheets have more degrees of freedom in their production that permit independent adjustment of the energy distribution along each beamlet. This can be used, for example, to increase the relative intensity of the polar beamlets (purple arrows, Fig. 2Bc and fig. S9Bc versus Fig. 2Aa and fig. S9Aa, respectively) and thereby strengthen ${\mathrm{OTF}}_{\mathrm{overall}}^{\mathrm{MB}}(k)$ near its *k_z_* support (e.g., purple arrows, Fig. 2, Bi and Bj, and fig. S9, Bi and Bj, versus Fig. 2, Ad and Ae, and fig. S9, Ae and Af). Advantages specific to other types of LLS confinement are discussed in the “Accuracy of image reconstruction” and “Excitation envelope and photobleaching” sections.

###### MB square LLSM

**Fig. 3.**
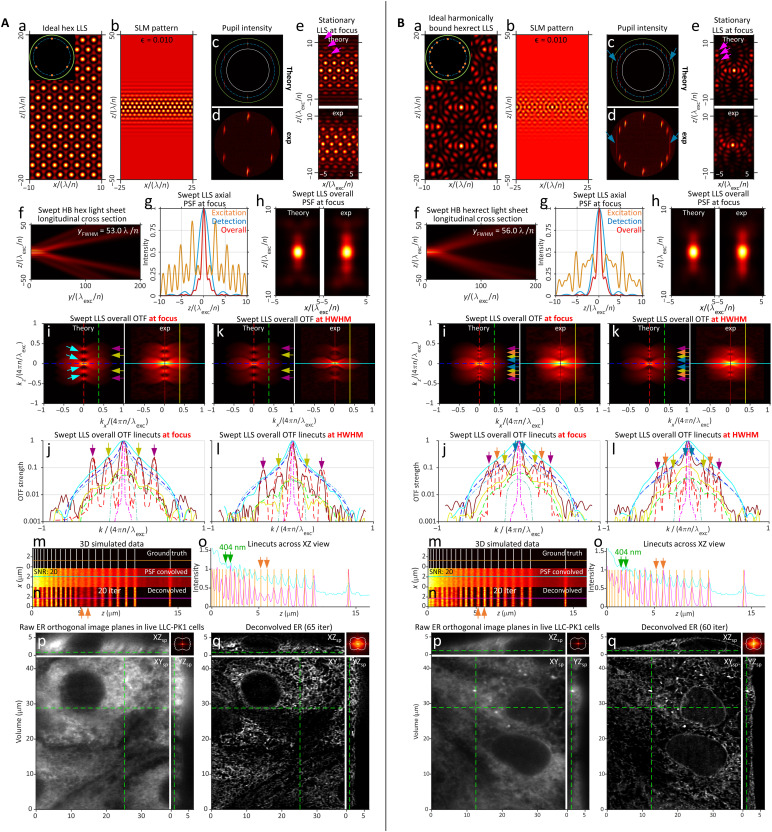
Theoretically and experimentally measured characteristics of two harmonic balanced LLSs. (**A**) Hexagonal LLS of NA_exc _= 0.50, σ_NA _= 0.075, ϵ = 0.010, NA_annulus _= 0.60/0.40, and *y*_FWHM _= 53.0 λ_exc_/*n*. (**B**) Hexagonal-rectangular LLS of NA_exc _= 0.50, σ_NA _= 0.15, ϵ = 0.010, NA_annulus _= 0.60/0.40, and *y*_FWHM _= 56.0 λ_exc_/*n*. Note the uniform strength of the axially shifted copies of OTF_det_ in ${\mathrm{OTF}}_{\mathrm{overall}}^{\mathrm{swept}}$ for both LLSs [subpanels i to l in (A) and (B)] and how this uniformity is maintained to the *y*_HWHM_ edges of their propagation ranges.

An MB LLS has pupil beamlets of extended length in *k_z_* that create bands in ${\mathrm{OTF}}_{\mathrm{exc}}^{\mathrm{scMB}}(k_{z})$, which, when convolved with OTF_det_(***k***), create an ${\mathrm{OTF}}_{\mathrm{overall}}^{\mathrm{scMB}}(k)$ where the discrete excitation-shifted copies of OTF_det_(***k***) are each smeared across a finite *k_z_* range. The beneficial result is a narrowing of the gaps in ${\mathrm{OTF}}_{\mathrm{overall}}^{\mathrm{scMB}}(k)$ (e.g., light blue arrows, fig. S9, Ad and Bi, versus Fig. 1Cf). The equatorial beamlets of the MB square LLS, being particularly long (green arrows, fig. S9, Aa and Bc), nearly completely fill the gaps in ${\mathrm{OTF}}_{\mathrm{overall}}^{\mathrm{scMB}}(k)$ (fig. S9, Ae and Bi) in the case of light sheets of length *y*_FWHM_~50 λ_exc_/*n* and NA_exc_ up to ∼0.30. As a corollary, most of the excitation energy is confined to the central peak (fig. S9Ah, green curve, and fig. S9Bg, orange curve), thereby minimizing out-of-focus background for applications such as single-molecule localization in thickly fluorescent specimens [(*27*); figure 3 of (*2*)].

Using an SLM to apply this strategy experimentally, we find good agreement with theory for the pupil intensity (fig. S9, Bc and Bd), the stationary excitation (fig. S9Be) and swept overall PSFs (fig. S9Bh) at the focal plane, as well as the overall OTF at both the focal plane (fig. S9, Bi and Bj) and near the HWHM of the light sheet (fig. S9, Bk and Bl). FSC on a simulated image (fig. S9Bm, bottom) of the stripe test pattern indicates that an optimal *z* resolution of 661 nm (green arrows, fig. S9Bo) is achieved after 10 RL iterations (movie S3, part 1). The corresponding CS simulation (green arrows, fig. S9Ak) with the same annulus achieves the same limit. In live experiments on LLC-PK1 cells (fig. S9Bp), the SLM-generated light sheet reaches an optimal result at 60 RL iterations for SNR ∼ 20 according to FSC, at which point the FT (fig. S9Bq, upper right inset) of the deconvolved image volume (movie S3, part 2) indicates that nearly all spatial frequencies within the support of ${\mathrm{OTF}}_{\mathrm{overall}}^{\mathrm{sMBSq}}(k)$ are detectable (fig. S9Bi).

As the ratio of NA_exc_ to *y*_FWHM_ increases, the troughs in ${\mathrm{OTF}}_{\mathrm{overall}}^{\mathrm{sSq}}(k)$ widen and deepen, and the OTF weakens near its *k_z_* support. For NA_exc_>0.30 and *y*_FWHM_>50λ_exc_/*n*, a hexagonal lattice then becomes a better choice. Conversely, however, an MB square LLS can remain effective at NA_exc_>0.30 in applications where a shorter light sheet can suffice. This includes small specimens such as bacteria, *Dictyostelium discoideum*, or the peripheral regions of cultured cells. For example, an SLM-generated MB square LLS with NA_exc_=0.41 and an annulus NA_max_/NA_min_= 0.60/0.40 (fig. S10, C and D) has a *y*_FWHM_ only 16λ_exc_/*n* long (fig. S10F) but also a strong and gap-free ${\mathrm{OTF}}_{\mathrm{overall}}^{\mathrm{sMBSq}}(k)$ out to its *k_z_* support (fig. S10, I and J). This yields a well-confined ${\mathrm{PSF}}_{\mathrm{overall}}^{\mathrm{sMBSq}}(x)$ (fig. S10H) with the energy in ${\mathrm{PSF}}_{\mathrm{exc}}^{\mathrm{sMBSq}}(z)$ largely confined to the central peak (fig. S10G, orange curve). A simulated image of the stripe test pattern with this light sheet (fig. S10M) at SNR = 20 reveals (movie S4, part 1) a minimum resolvable stripe separation after 10 RL iterations of 404 nm (green arrows, fig. S10O), close to the limit of 407 nm from eq. S10b with (NA_exc_)_max_= 0.60. To cover the same *y*_FWHM_~50λ_exc_/*n* FOV as most other light sheets studied here, we imaged live LLC-PK1 cells across four tiles perpendicular to the specimen substrate (fig. S10P). After tile stitching and 85 iterations of RL deconvolution as indicated by FSC, the resulting image volume (fig. S10Q and movie S4, part 3) recovers specimen spatial frequencies (fig. S10Q, inset) within most of the theoretical support region of fig. S10I.

###### MB hexagonal LLSM

While the ±2*k_o_*NA_exc_ harmonics of the swept ideal square lattice are stronger than those of the ideal hexagonal lattice (purple arrows, Fig. 1, Bd and Bf versus Cd and Cf), the inverse is often true for the corresponding MB LLSs (purple arrows, Fig. 2, Bi and Bj, versus fig. S9, Bi and Bj), because the long equatorial pupil beamlets of the square LLS overweight the DC region of ${\mathrm{OTF}}_{\mathrm{overall}}^{\mathrm{scMB}}(k)$. However, for hexagonal MB LLSs, the shorter flanking beamlets shrink the OTF gaps without unnecessary overweighting of the DC region. For example, both simulated CS (Fig. 2Af) and experimental SLM-generated (Fig. 2Bh) and MB hexagonal LLS at NA_exc_ = 0.40 and 0.43 respectively exhibit more tightly confined swept overall PSFs than the corresponding square lattices at NA_exc_ = 0.30. Notably, the small OTF gaps that remain in both cases (light blue arrows, Fig. 2, Ad and Bi) are partially filled in the experimental OTF with the SLM. After RL deconvolution (movie S5, part 1), the SLM and CS lattices are capable in simulations of resolving all line pairs in the stripe test pattern down to 514 and 440 nm, respectively (green arrows, Fig. 2, Ak and Bo). Furthermore, an RL deconvolved image volume of live LLC-PK1 cells shows biologically realistic ER structure with no obvious artifacts (Fig. 2Bq and movie S5, part 3), and the FFT of this volume shows the recovery of spatial frequencies throughout most of the support region, notably including those associated with the OTF gaps (Fig. 2Bq, upper right inset).

##### Axially confined LLSM

Rather than creating LLSs from coherent MB light sheets at the specific periods T of Eq. 14 corresponding to lattices of specific symmetries, one can start from an ideal lattice of the desired symmetry (Fig. 1) and modify its discrete points of pupil illumination in ways that confine the lattice while simultaneously optimizing other desired properties. By doing so, one is not wedded to pupil beamlets whose lengths are dictated solely by the annulus.

One such optimization is to require that the light sheet be AC in a specific way. This is a natural constraint when out-of-focus background and/or photobleaching/phototoxicity is a concern. Since every ideal 2D lattice is composed of a finite set of plane waves

$$E_{\mathrm{lattice}}^{\mathrm{ideal}}(x)=\sum_{m=1}^{M}E_{m}\exp[ik_{m}\cdot x]=E_{\mathrm{sample}}(x)$$ (17a)

an AC LLS is defined by

$$E_{\mathrm{sample}}^{\mathrm{ACLLS}}(x)=B(z)\sum_{m=1}^{M}E_{m}\exp[ik_{m}\cdot x]$$ (17b)

where the bounding function *B*(*z*) → 0 as *z* → ∞. Since *E*_pupil_(*x_p_*, *z_p_*) = FT{*E*_sample_(*x*, 0, *z*)}, this gives

$$E_{\mathrm{pupil}}^{\mathrm{ACLLS}}(k_{x},k_{z})=\sum_{m=1}^{M}E_{m}\delta [k_{x}-{\left(k_{x}\right)}_{m}]\tilde{B}[k_{z}-{\left(k_{z}\right)}_{m}]$$ (18a)

where $\tilde{B}(k_{z})\equiv \mathrm{FT}\{B(z)\}$. In other words, in an AC LLS, the discrete points of illumination in the pupil plane (figs. S11A to S13A, insets) are replaced by stripes parallel to the *k_z_* axis centered at these points, all of which are bound equally (figs. S11C to S13C).

A common bounding function, used in (*2*) and here, unless otherwise specified, is a Gaussian: $B(z)=\exp(-z^{2}/\sigma_{z}^{2})$, where σ*_z_* is the 1/*e*^2^ axial width of ${\mathrm{PSF}}_{\mathrm{exc}}^{\mathrm{fixed}}(x)$ and ${\mathrm{PSF}}_{\mathrm{exc}}^{\mathrm{swept}}(x)$, in which case $\tilde{B}(k_{z})$ or, equivalently, $\tilde{B}(z_{p})$ is also Gaussian:

$$\tilde{B}(z_{p})=\exp[-z_{p}^{2}/{\left(\sigma_{\mathrm{NA}}\cdot {\mathrm{NA}}_{\mathrm{exc}}F\right)}^{2}]$$ (18b)

σ_NA_ then describes, in terms of effective NA, the 1/*e*^2^ width of the intensity in the rear pupil of the 1D Gaussian beamlets that replace the discrete points of illumination of the ideal lattice. Experimentally, we generated these light sheets by calculating the desired LLS electric field *E*_exc_(*x*, 0, *z*) at the specimen focal plane from the inverse FT (Eq. 1d) of $E_{\mathrm{pupil}}^{\mathrm{ACLLS}}(k_{x},k_{z})$ from Eq. 18 and then using Eq. 4 to determine the binary phase Φ_SLM_(*x*, *z*) at the SLM needed to produce the desired AC LLS. The results and comparisons to theoretical simulations are shown for light sheets of SW, square, and hexagonal symmetry in figs. S11 to S13, respectively, and described in note S7.

AC LLSs have two advantages over their MB counterparts. First, because the pupil beamlets of all AC LLSs have the same length and intensity (e.g., purple and gold arrows, fig. S13C), their *k_z_* shifted copies of OTF_det_(***k***) in ${\mathrm{OTF}}_{\mathrm{overall}}^{\mathrm{sMBSq}}(k)$ are stronger than in the MB case (e.g., purple arrows, fig. S13I versus fig. 2Bi), where the beamlet length decreases with increasing *k_z_*. Second, because the confinement of AC LLSs is determined by *B*(*z*) as encoded in Φ_SLM_(*x*, *z*), the annulus can be adjusted independent of the desired propagation length (unlike in the MB case) to filter out undiffracted light and either admit or reject higher diffraction orders from the SLM. For example, the cropping factor ϵ (Eqs. 4d and 4e) and the bounding envelope *B*(*z*) (Eq. 18b) work together to produce a sharply bound version of the desired AC hexagonal lattice at the binary SLM (fig. S13B). This creates a rect(*z*) bounding function to the diffracted field *E*_SLM_(*x*, *z*) = *E_o_* exp[−Φ_SLM_(*x*, *z*)] and, since *E*_pupil_(*k_x_*, *k_z_*) ∝ FT*_xz_*[*E*_SLM_(*x*, *z*)], a sinc(*k_z_*) bounding function to each beamlet in the rear pupil (pink and light blue arrows, fig. S13C). These advantageously fill the gaps (pink and light blue arrows, fig. S13, I and J) in ${\mathrm{OTF}}_{\mathrm{overall}}^{\mathrm{sACHex}}(k)$ between the five shifted copies of OTF_det_(***k***) seen in the MB case (Fig. 2, Bi and Bj) without substantially affecting the overall propagation length *y*_FWHM_ (fig. S13F).

One notable disadvantage of AC LLSs is that because their pupil beamlets all have the same length, and (NA_max_)*_b_* − (NA_min_)*_b_* for a beamlet of a given length increases with increasing distance from the pupil equatorial line (fig. S8), (*y*_FWHM_)*_b_* for these beamlets also decreases (Eq. 15). Thus, for example, the propagation length of the equatorial beamlets of the AC square LLS in fig. S12 is >8× longer than its polar ones (fig. S14B), whereas these lengths are the same to within ~20% (fig. S14A) for the MB square LLS. Similarly, the difference between the propagation lengths of the flanking and polar beamlets of the AC hexagonal LLS of fig. S13 is ~2× greater (fig. S14D) than that seen for the corresponding beamlets of the MB hexagonal LLS of Fig. 2B. Consequently, while the strength of the ±*k_o_*NA_exc_ and ±2*k_o_*NA_exc_ shifted copies of OTF_det_(***k***) in ${\mathrm{OTF}}_{\mathrm{overall}}^{\mathrm{sMBSq}}(k)$ (gold and purple arrows, respectively, Fig. 2, Bi and Bj) for the MB hexagonal lattice is little changed from the focus to *y*_FWHM_ (Fig. 2, Bi to Bl), the same copies in its AC counterpart decrease in strength by ~3 and 10×, respectively, over the same distance (fig. S13, I to L), making the recovery of information close to the theoretical axial support of eq. S10c over the entire propagation length extremely difficult.

##### Harmonic balanced LLSM

Given the trade-offs between the MB and AC approaches to producing LLSs, it is natural to consider whether their advantages can be combined in a hybrid approach. Such an optimized LLS would have an overall OTF both uniform and strong everywhere within its 3D support and maintain this strength and uniformity over its designed propagation range. It would also have sidelobes confined enough that the fluorescence they generate can be converted to useful signal to minimize unnecessary photobleaching. We can come closer to this ideal by combining the ideas above as follows:

1) Choose the symmetry (see the “Considerations in choosing a lattice of a given symmetry” section and Fig. 1), NA_exc_, and propagation length *y*_FWHM_ of the desired LLS. The symmetry determines the wave vectors ***k****_b_* of the underlying ideal 2D lattice. We exclude the square lattice since, although it is well suited for applications requiring minimal sidelobe excitation, such as single-molecule detection, its overall OTF is unnecessarily weighted toward DC by its equatorial beamlets. For most other applications, the axial SW or hexagonal lattice is a better choice.

2) Model the pupil electric field *E_b_*(*k_x_*, *k_z_*) of each beamlet of the desired LLS as a 1D Gaussian centered ***k****_b_*NA_exc_/*k* having a 1/*e* half width of (σ_NA_)*_b_* and peak amplitude (*E_o_*)_b_:

$$E_{b}(k_{x},k_{z})={\left(E_{o}\right)}_{b}\delta [k_{x}-(k_{b}\cdot {\hat{e}}_{x_{\mathrm{optical}}}){NA}_{\mathrm{exc}}]\times \exp\left\{-{\left[k_{z}-(k_{b}\cdot {\hat{e}}_{z_{\mathrm{optical}}}){\mathrm{NA}}_{\mathrm{exc}}\right]}^{2}/{\left(\sigma_{\mathrm{NA}}\right)}_{b}^{2}\right\}$$ (19)

Other confinement functions such as sinc(α*_b_k_z_*), where α*_b_* is an independently adjustable confinement factor for each beamlet, are also possible. Because a sinc function decays more slowly than a Gaussian for the same full width at half maximum (FWHM), it can better fill OTF gaps, but at the cost of lower OTF uniformity over the propagation length.

3) Find the relative value of (σ_NA_)*_b_* for each beamlet, which gives it the same propagation length (*y*_FWHM_)*_b_* = *y*_FWHM_ as every other beamlet. By Eq. 15, (*y*_FWHM_)*_b_* of any beamlet is proportional to the NA range

$$\text{Δ}{\mathrm{NA}}_{b}={\mathrm{NA}}_{b}^{+}-{\mathrm{NA}}_{b}^{-}$$ (20a)

it covers in the pupil. For our 1D Gaussian beamlets, we estimate ΔNA*_b_* from the NA at the 1/*e* points $k_{b}{\mathrm{NA}}_{\mathrm{exc}}\pm {\left(\sigma_{\mathrm{NA}}\right)}_{b}{\hat{e}}_{z_{\mathrm{optical}}}$ of *E_b_*(*k_x_*, *k_z_*), akin to the points $k_{z}^{\pm }$ in fig. S7

$${\mathrm{NA}}_{b}^{\pm }\approx \sqrt{{\mathrm{NA}}_{\mathrm{exc}}^{2}\pm 2{\mathrm{NA}}_{\mathrm{exc}}|k_{b}\cdot {\hat{e}}_{z_{\mathrm{optical}}}|{\left(\sigma_{\mathrm{NA}}\right)}_{b}/k_{o}+{\left(\sigma_{\mathrm{NA}}\right)}_{b}^{2}}$$ (20b)

This assumes that the beamlet does not cross the equatorial *k_z_* = 0 line, which is true for all beamlets of all lattices in Fig. 1 except the square one. Since $|k_{b}\cdot {\hat{e}}_{z_{\mathrm{optical}}}|{\left(\sigma_{\mathrm{NA}}\right)}_{b}/k_{o}<$ 1 and usually (σ_NA_)*_b_*/NA_exc_≪ 1, to lowest order in (σ_NA_)*_b_*/NA_exc_, we find

$${\left(y_{\mathrm{FWHM}}\right)}_{b}\propto \text{Δ}{\mathrm{NA}}_{b}\approx 2|k_{b}\cdot {\hat{e}}_{z_{\mathrm{optical}}}|{\left(\sigma_{\mathrm{NA}}\right)}_{b}/k_{o}$$ (20c)

If we choose one of the beamlets as the reference, then by Eq. 20c, (σ_NA_)*_b_* of the other beamlets is

$${\left(\sigma_{\mathrm{NA}}\right)}_{b}\approx \frac{\text{Δ}{\mathrm{NA}}_{\mathrm{ref}}}{\text{Δ}{\mathrm{NA}}_{b}}{\left(\sigma_{\mathrm{NA}}\right)}_{\mathrm{ref}}$$ (20d)

4) Find (σ_NA_)_ref_ in terms of the desired *y*_FWHM_ of the entire light sheet and therefore, by Eq. 20d, (σ_NA_)*_b_* for all other beamlets. To do so, we choose a polar beamlet, for which

$$|k_{\mathrm{ref}}\cdot {\hat{e}}_{z_{\mathrm{optical}}}|=k_{o}$$ (21)

as the reference. By Eq. 15, *y*_FWHM_ and (σ_NA_)_ref_ are then related by

$$y_{\mathrm{FWHM}}\approx \frac{\lambda_{\mathrm{exc}}/n}{2\left(\sqrt{1-{\left\{[{\mathrm{NA}}_{\mathrm{exc}}-{\left(\sigma_{\mathrm{NA}}\right)}_{\mathrm{ref}}]/n\right\}}^{2}}-\sqrt{1-{\left\{[{\mathrm{NA}}_{\mathrm{exc}}+{\left(\sigma_{\mathrm{NA}}\right)}_{\mathrm{ref}}]/n\right\}}^{2}}\right)}$$ (22a)

Expanding to lowest order in (σ_NA_)_ref_/NA_exc_, this yields

$${\left(\sigma_{\mathrm{NA}}\right)}_{\mathrm{ref}}\approx \frac{\sqrt{n^{2}-{\mathrm{NA}}_{\mathrm{exc}}^{2}}}{4{\mathrm{NA}}_{\mathrm{exc}}}\frac{\lambda_{\mathrm{exc}}}{y_{\mathrm{FWHM}}}$$ (22b)

5) Individually adjust the electric field amplitudes (*E_o_*)*_b_* of the beamlets at the pupil so that the amplitudes (*E*_focus_)*_b _*of the Gaussian beams they produce at the focal point in the specimen are identical. For example,

$${\left(E_{\mathrm{focus}}\right)}_{b}={\left(E_{\mathrm{focus}}\right)}_{\mathrm{ref}}$$ (23a)

By doing so, all nonzero harmonics of the swept excitation OTF are identical, leading to *k_z_* shifted copies of OTF_det_(***k***) of equal strength, and thus a more uniform OTF_overall_(***k***) throughout the support region. To do so, we note that, by energy conservation

$${\left(E_{\mathrm{focus}}\right)}_{b}\propto \frac{{\left(E_{o}\right)}_{b}}{{\left(\sigma_{\mathrm{focus}}\right)}_{b}}$$ (23b)

However, for every Gaussian beam

$${\left(\sigma_{\mathrm{focus}}\right)}_{b}\propto \frac{1}{{\left(\sigma_{\mathrm{NA}}\right)}_{b}}$$ (23c)

Combining Eqs. 20d and 23a to 23c then gives the desired relationship between the beamlet amplitudes in the pupil

$${\left(E_{o}\right)}_{b}=\frac{\text{Δ}{\mathrm{NA}}_{b}}{\text{Δ}{\mathrm{NA}}_{\mathrm{ref}}}{\left(E_{o}\right)}_{\mathrm{ref}}$$ (23d)

6) Use the pupil pattern *E*_pupil_(*k_x_*, *k_z_*) = ∑ *E_b_*(*k_x_*, *k_z_*) described by Eq. 19 to determine *E*(*x*, 0, *z*) according to Eq. 1d and then find the SLM grayscale pattern Φ_SLM_(*x*, *z*) needed to generate the LLS from Eqs. 4a to 4c.

7) Since (σ_NA_)*_b_* and (σ_NA_)_ref_ in Eqs. 20d and 22b are estimates, adjust (σ_NA_)_ref_ empirically and all other (σ_NA_)*_b_* according to Eq. 20d to fine-tune *y*_FWHM_ to the desired length.

Because this procedure is designed to produce LLSs of equal harmonic strength that maintain their equality throughout their propagation range, we term them harmonic balanced (HB) LLSs. The examples shown for hexagonal and hexrect lattices of NA_exc_ = 0.50 and *y*_FWHM_~ 50λ_exc_/*n* in Fig. 3 show that these goals are largely achieved in practice, with all harmonics maintaining comparable relative amplitudes throughout the propagation range (colored arrows, Fig. 3, Ai to Al and Bi to Bl, and movies S6 and S7). Likewise, the individual harmonic bands of both lattices are all close to the desired length (fig. S15), with the exception of the ±*k_o_*NA_exc_/2 band of the hexrect LLS, where the two beamlets in each band merge into a pair of longer DC bands (blue arrows, Fig. 3, Bc and Bd). This may be because the assumption (σ_NA_)*_b_*/NA_exc_≪ 1 used to derive Eq. 23d is not valid in this case. If desired, (σ_NA_)*_b_* for these beamlets could be empirically adjusted to achieve the desired *y*_FWHM_, but even as is, the effect on the overall OTF is not substantial.

Both HB light sheets resolve line pairs in the simulated stripe test pattern down to 404 nm after 20 RL iterations (green arrows, Fig. 3, Ao and Bo, and movies S6 and S7, part 1), consistent with their mutual NA_exc_ = 0.50. However, the modulation depth across the pattern is deeper and more uniform in the hexrect case (orange arrows, Fig. 3, An and Ao and Bn and Bo), perhaps due to the deeper OTF troughs of a hexagonal lattice at this NA (light blue arrows, Fig. 3, Ai and Aj), although these could, in principle, be partially filled in as demonstrated in AC case (pink and light blue arrows, fig. S13, I and J) by using a higher cropping factor ϵ to create higher diffraction orders flanking the beamlets in the pupil (pink and light blue arrows, fig. S13C). Nevertheless, even as is, live imaging of LLC-PK1 cells reveals 3D ER structure with no obvious artifacts in both the hexagonal and hexrect cases after 65 and 60 RL iterations, respectively, as indicated by FSC (Fig. 3, Aq and Bq, and movies S6 and S7, part 3), and FFTs of deconvolved image volumes show recovery of spatial frequencies throughout most of the support region in both cases (Fig. 3, Aq and Bq, upper right inset).

### Comparisons between light sheets

To better compare the strengths and weaknesses of the light sheets discussed above, we summarize their various properties across their entire propagation length. Given the dependence of the axial resolution, axial confinement of the excitation, and uniformity of the OTF on the propagation length, all comparisons are for light sheets of length *y*_FWHM_~ 50 λ_exc_/*n* (consistent for imaging across whole cultured cells) unless otherwise specified.

#### Overall swept OTF

As argued in (*2*), ${\mathrm{OTF}}_{\mathrm{overall}}^{\mathrm{swept}}(k)$ gives the most comprehensive and quantitative measure of the ability of a microscope to accurately measure the spatial frequencies in a specimen in the presence of noise. To characterize its variation along the axis of propagation *y*, we calculated ${\mathrm{OTF}}_{\mathrm{overall}}^{\mathrm{swept}}(k_{x},y,k_{z})$ at intervals of Δ*y* = 3λ_exc_/*n* (movie S8) from the focal plane (*y* = 0) to ~1.5*y*_HWHM_ (*y* = 39λ_exc_/*n*) and plotted linecuts through ${\mathrm{OTF}}_{\mathrm{overall}}^{\mathrm{swept}}(k_{x},y,k_{z})$ (movie S9) along *k_x_* = 0 (red), $k_{x}=2\pi \lambda_{\mathrm{exc}}/n=k_{x}^{\max}/2$ (green), and *k_z_* = 0 (blue).

Focusing first on the Gaussian light sheet (fig. S3, upper left, and movie S8), although it has the narrowest divergence of all light sheets for distances *y* past the common *y*_HWHM_ of all light sheets considered above (fig. S16), it diverges the fastest within the propagation range ∣*y*∣ ≲ *y*_HWHM_ that is most relevant to light sheet microscopy. The modest *z*-resolution extension and filling of the missing cone of OTF_det_(***k***) it provides at the focal plane are mostly lost by *y* = 24λ_exc_/*n* ≈ *y*_HWHM_ (movies S8 and S9). In contrast, the sinc beam (fig. S4, upper middle, and movies S8 and S9) offers slightly superior *z* resolution at the focal plane for the same propagation range and yet better retains that resolution as it propagates, as evidenced by a ∼10× stronger overall OTF near the *k_z_* support at *y* = 24λ_exc_/*n*. However, the beams in (*10*) and (*11*) that were compared to LLSs were sinc in nature, not Gaussian, as they were created with a uniform, sharply bound stripe of illumination in the pupil, according to Eq. 6a. Thus, any conclusions regarding Gaussian versus LLSs in these works are invalid.

The evolution of ${\mathrm{OTF}}_{\mathrm{overall}}^{\mathrm{swept}}(k_{x},y,k_{z})$ with increasing *y* for the MB and AC square LLSs (upper right and middle left, movies S8 and S9) demonstrate the trade-offs of these two confinement strategies. By Eqs. 10b and 11, ${\mathrm{PSF}}_{\mathrm{exc}}^{\mathrm{swept}}(y,z)$ and ${\mathrm{OTF}}_{\mathrm{overall}}^{\mathrm{swept}}(k_{x},y,k_{z})$ are each the incoherent sum of ${\left[{\mathrm{PSF}}_{\mathrm{exc}}^{\mathrm{swept}}(y,z)\right]}_{m}$ and ${\left[{\mathrm{OTF}}_{\mathrm{overall}}^{\mathrm{swept}}(k_{x},y,k_{z})\right]}_{m}$ formed by each pupil band individually. The two equatorial bands of the MB square LLS, being much longer than the AC ones (fig. S9, Bc and Bd, versus fig. S12, C and D), create a pair of contributing light sheets ${\left[{\mathrm{PSF}}_{\mathrm{exc}}^{\mathrm{swept}}(y,z)\right]}_{m}$ more intense and much more confined in both *y* and *z* (magenta and aqua arrows, respectively, fig. S14, A versus B). However, this intense focus is heavily weighted toward *k_z_* values lower than those of ${\left[{\mathrm{PSF}}_{\mathrm{exc}}^{\mathrm{swept}}(y,z)\right]}_{m}$ contributed by the polar band. Thus, near the focal plane, ${\mathrm{OTF}}_{\mathrm{overall}}^{\mathrm{swept}}(k_{x},y,k_{z})$ is weaker near the *k_z_* support for the MB square LLS than for the AC one. On the other hand, the long equatorial bands in the MB case have a range (NA_min_)*_b_* to (NA_max_)*_b_* similar to that of the polar band and hence, by Eq. 15, similar propagation lengths for their corresponding individual light sheets. This leads to a more uniform ${\mathrm{OTF}}_{\mathrm{overall}}^{\mathrm{swept}}(k_{x},y,k_{z})$ over the propagation range than in the AC LLS, where ${\left[{\mathrm{PSF}}_{\mathrm{exc}}^{\mathrm{swept}}(y,z)\right]}_{m}$ associated with the polar band decays far more rapidly with increasing *y* than that associated with the equatorial bands (magenta arrows, fig. S14B).

Because the axial SW light sheet, whether produced by the MB or AC method, has only a single pupil band, its ${\mathrm{OTF}}_{\mathrm{overall}}^{\mathrm{swept}}(k_{x},y,k_{z})$ is not subject to these trade-offs, and it remains strong throughout its support throughout its propagation range (center, movie S8). As a result, it is the preferred light sheet type in cases where its OTF gaps are not too large to preclude accurate image restoration (e.g., Eq. 13 and figs. S11Q versus S17Q) and the sidelobes of ${\mathrm{PSF}}_{\mathrm{exc}}^{\mathrm{swept}}(x)$ do not lead to excessive photobleaching (see the “Harmonic balanced LLSM” section).

For higher NA_exc_, the additional *k_z_* = ± *k_o_*NA_exc_ harmonics contributed by the flanking pupil bands of the hexagonal LLS help fill these gaps (Fig. 2, Bc and Bi). In the MB case, all three bands generate contributing terms ${\left[{\mathrm{PSF}}_{\mathrm{exc}}^{\mathrm{swept}}(y,z)\right]}_{m}$ to the overall light sheet that have similar propagation lengths (fig. S14C), so the ±*k_o_*NA_exc_ and ±2*k_o_*NA_exc_ shifted copies of OTF_det_(***k***) in ${\mathrm{OTF}}_{\mathrm{overall}}^{\mathrm{swept}}(k_{x},y,k_{z})$ maintain their relative strengths throughout the propagation range (lower left, movie S8). In contrast, the ±2*k_o_*NA_exc_ shifted copies in the AC hexagonal LLS decay rapidly in strength as ∣*y*∣ → *y*_HWHM_ (center right, movies S8 and S9) due to the shorter propagation length of ${\left[{\mathrm{PSF}}_{\mathrm{exc}}^{\mathrm{swept}}(y,z)\right]}_{m}$ for the polar band (fig. S14D). On the other hand, the higher *k_z_* diffraction orders in the pupil bands of the AC hexagonal LLS (pink and light blue arrows, fig. S13C) better fill the OTF troughs seen in the MB hexagonal case.

As expected, the HB hexagonal and HB hexrect lattices combine the best of the MB and AC approaches: By enforcing a similar (NA_max_)*_b_* − (NA_min_)*_b_* for every pupil beamlet (Fig. 3, Ac and Bc), the corresponding beamlets in the specimen have comparable propagation length (fig. S15), and hence, the relative strengths of the shifted copies of OTF_det_(***k***) in ${\mathrm{OTF}}_{\mathrm{overall}}^{\mathrm{swept}}(k_{x},y,k_{z})$ remain nearly constant throughout this range (bottom center and bottom right, movies S8 and S9). In addition, by adjusting the relative intensities of the pupil beamlets to produce beamlets of similar intensity at the focal point, these shifted copies create ${\mathrm{OTF}}_{\mathrm{overall}}^{\mathrm{swept}}(k_{x},y,k_{z})$ of more uniform strength (Fig. 3, Ai to Al and Bi to Bl, and movies S8 and S9). This facilitates the recovery of information near the axial limit of the support (Fig. 3, Aq and Bq, inset), even at the higher NA_max_ afforded by the stronger OTF [0.58 and 0.60 in Fig. 3 (A and B) versus 0.47 for the MB and AC LLSs in figs. 2B and S13].

#### Spatial resolution

**Fig. 4.**
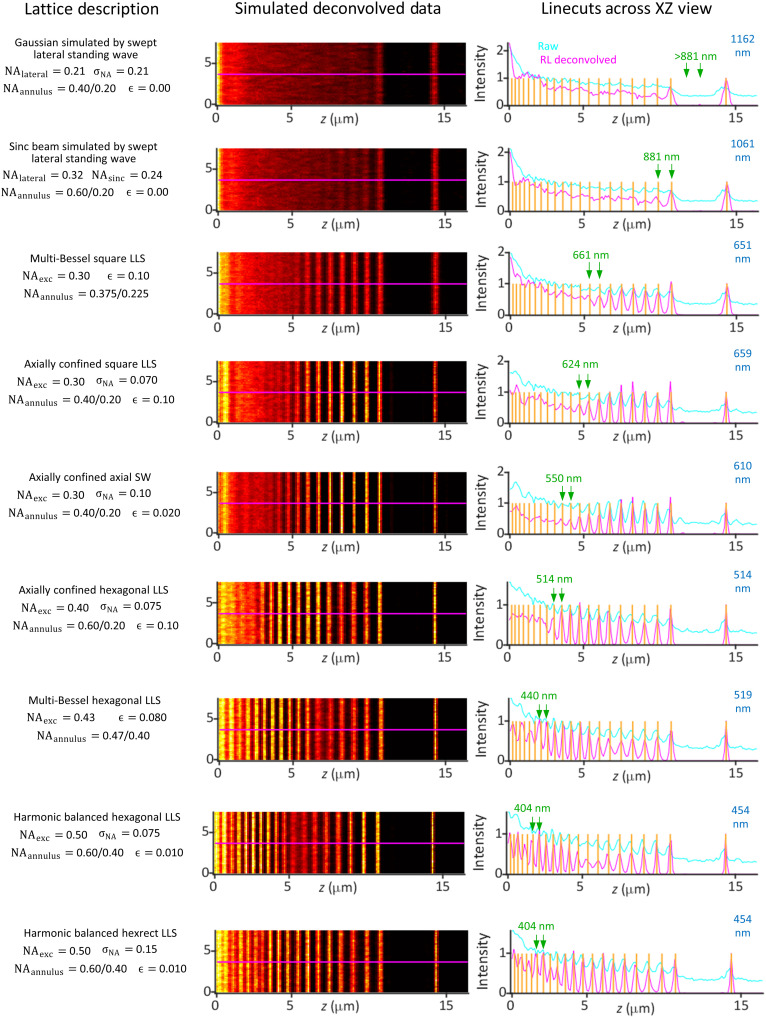
Comparative simulated images after RL deconvolution from a line pattern of variable spacing for nine light sheets, all of length *y*_FWHM_*~*50 λ_exc_/*n*. Green arrows in linecuts at right show the smallest resolvable line pair in each case, and the theoretical limit for linear deconvolution is given in blue at right. Note the progression to higher resolution and improved modulation depth from Gaussian (top) to harmonic balanced lattice (bottom) light sheets.

**Fig. 5.**
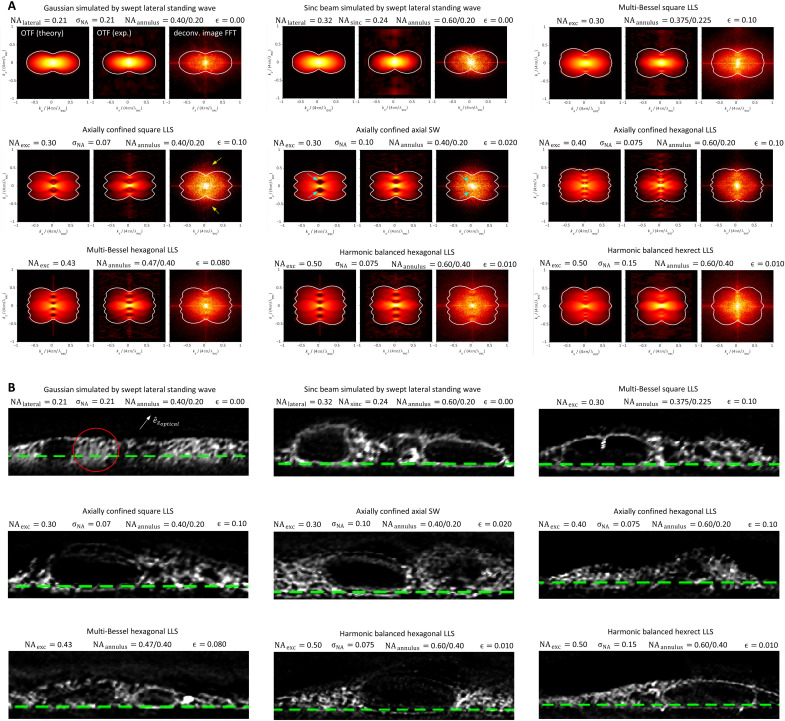
Real and frequency space comparisons of the experimentally attained resolution for the nine light sheets in Fig. 4. (**A**) Theoretical and experimental ${\mathrm{OTF}}_{\mathrm{overall}}^{\mathrm{swept}}$ (left and center in each subpanel) and measured spatial frequency distribution in the *xz*_specimen_ plane (right in each subpanel) of RL-deconvolved image volumes of the ER in live LLC-PK1 cells. The distributions assume an elliptical shape rather than that of the theoretical support boundary (white) because the frequency distribution of the ER itself is largely isotropic and falls steeply in strength from its DC peak. (**B**) Comparative post-RL deconvolution orthoslices in the *xz*_specimen_ plane at SNR = 30 through the ER, showing no evidence of sidelobe artifacts for any case. The poor resolution of the Gaussian light sheet in the optical axial direction ${\hat{e}}_{z_{\mathrm{optical}}}$ (upper left subpanel) renders the perinuclear ER difficult to resolve (red circle).

The theoretical resolution limits of the nine light sheets in movies S8 and S9 are summarized in table S1, using the definitions and equations of note S2 and fig. S2. Both the experimental configuration used in the measurements here (${\mathrm{NA}}_{\mathrm{exc}}^{\mathrm{obj}}=0.60$, NA_det_ = 1.0) and that used in (*1*) (${\mathrm{NA}}_{\mathrm{exc}}^{\mathrm{obj}}=0.70$, NA_det_ = 1.1) are included for comparison. Estimates of the resolution limit $R({\hat{e}}_{z_{\mathrm{optical}}})$ for all nine based on simulated images of a variable pitch stripe pattern are summarized in Fig. 4, along with the corresponding theoretical limit (blue) from table S1. Measurements of the detectable spatial frequencies from the ER within live LLC-PK1 cells are summarized for all nine light sheets in Fig. 5A and shown along with a boundary curve indicating the theoretical support in each case. Last, deconvolved orthoslices from the cell images are compared (Fig. 5B) in the *xz*_specimen_ plane that exhibits the greatest resolution gain with increasing NA_exc_ of the light sheet but also the greatest potential for sidelobe ghost artifacts if the data are not correctly deconvolved.

Considering first the MB and AC square LLS, we find close agreement between the theoretical $R({\hat{e}}_{z_{\mathrm{optical}}})$ (651 and 659 nm, respectively) and corresponding simulation-based estimates (661 and 624 nm, respectively; figs. S9B and S12). Notably, these limits are well beyond the theoretical estimates of 1162 and 1017 nm for the Gaussian and sinc light sheets, respectively (figs. S3 and S4), as well as the simulation-based estimate of 881 nm in the sinc case. Experimentally, after deconvolution, all four light sheets recover sample spatial frequencies across the majority of their support regions, although the support itself differs in extent in each case based on the ${\mathrm{NA}}_{\mathrm{exc}}^{\max}$ needed to achieve the common light sheet propagation length of *y*_FWHM_~ 50λ_exc_/*n*. Given that $R({\hat{e}}_{x_{\mathrm{optical}}}^{\mathrm{swept}})=$ 260 nm for all light sheets, the resolution for the Gaussian and sinc light sheets is particularly anisotropic. This is evidenced as a smearing of sample structure along the ${\hat{e}}_{z_{\mathrm{optical}}}$ axis in deconvolved *xz*_specimen_ orthoslices (white arrow, upper left, Fig. 5B) that is most pronounced in the Gaussian case, where this smearing makes it difficult to resolve ER tubules and sheets in the dense perinuclear region (red circle).

These results directly conflict with the conclusions of (*10*–*12*) that the resolution of Gaussian (in actuality, sinc, as described above) and square LLSs is similar for comparable propagation length. There are several possible reasons for this discrepancy that are explored in depth in note S8, but in brief, although in (*10*) an MB square LLS with an annulus of NA_annulus_= 0.55/0.44 (fig. S18A) identical to that of one of the MB square LLSs in (*2*) was used for comparison (fig. S18B), in other important aspects (NA_exc_ of the equatorial bands and the SLM cropping factor ϵ) these light sheets differed. As a result, the LLS in (*10*) had far weaker ±2*k_o_*NA_exc_ harmonics and much deeper troughs in ${\mathrm{OTF}}_{\mathrm{overall}}^{\mathrm{swept}}(k_{x},y,k_{z})$ than the corresponding one in (*2*), leading to lower resolution dominated by the sinc-like equatorial bands of the square LLS rather than the higher-resolution polar bands as in (*2*).

Overall, Figs. 4 and 5 demonstrate that all seven LLSs were able to meet or slightly exceed their theoretical resolution limits as defined by $R({\hat{e}}_{z_{\mathrm{optical}}})$ and the support boundaries of Fig. 5A, even at an SNR of 30 compatible with long-term noninvasive live cell imaging. Given that these limits are defined by ${\mathrm{NA}}_{\mathrm{exc}}^{\max}$ (table S1), which, in the hexagonal and hexrect cases, can approach the limits ${\mathrm{NA}}_{\mathrm{exc}}^{\mathrm{obj}}=$ 0.60 (here) or 0.70 [in (*2*)], the resolution along ${\hat{e}}_{z_{\mathrm{optical}}}$ can reach 3.3 or 2.8× that of a Gaussian light sheet of identical length *y*_FWHM_~ 50λ_exc_/*n*, and the maximum axial resolution (*R*_*z*_optical__)_max_ at the widefield bowtie position can reach 3.8 or 4.6× that of a widefield microscope at λ_det_ = 520 nm and ${\mathrm{NA}}_{\mathrm{exc}}^{\det}=$ 1.0 (here) or 1.1 [in (*2*)]. These ratios increase further with increasing light sheet length, since ${\mathrm{NA}}_{\mathrm{exc}}^{\max}$ remains unchanged for an LLS but decreases as $1/\sqrt{y_{\mathrm{FWHM}}}$ for a Gaussian one.

#### Accuracy of image reconstruction

One surprising finding on comparing all nine light sheets is the apparent recovery of sample spatial frequencies by RL deconvolution outside the theoretical support: In Fig. 4, seven of them were able to resolve line pairs in simulated images separated by (green) less than the theoretical limit $R({\hat{e}}_{z_{\mathrm{optical}}})$ (blue), and all nine cellular FFTs exhibited partial filling of the outward facing pair missing cones associated with the furthest shifted copies of OTF_det_(***k***) (e.g., yellow arrows, middle left, Fig. 5A). Furthermore, FSC-guided RL deconvolution was able to fill the troughs in the overall OTFs of all seven LLSs (e.g., light blue arrows, center, Fig. 5A). Together, these observations suggest that, unlike linear Wiener deconvolution, iterative Bayesian restoration with a non-negative prior can recover the otherwise missing information in the OTF troughs of LLSs and slightly expand the axial support while also producing sharper images (e.g., fig. S19A). However, even Wiener deconvolution can produce accurate reconstructions for light sheets with strong excitation sidelobes, such as (fig. S19, B and C) the MB hexagonal light sheet of Fig. 2B, where the pair of sidelobes flanking the central excitation peak is >75% of that peak’s intensity (Fig. 2Bg, orange curve).

The problem of creating accurate representations of sample structure from images acquired by a microscope having an overall PSF with strong sidebands and, equivalently, deep overall OTF troughs was investigated previously (*28*–*30*) in comparisons of 4Pi (*31*), SW [SWM, (*32*)], and image interference and incoherent interference illumination [I5M, (*33*)] microscopy. The findings there and their relevance to LLS are considered in depth in note S9. Briefly, the sidelobe conditions for which RL deconvolution produces artifact-free images of sample structure in 4Pi microscopy are consistent with the conditions that produce accurate reconstructions of simulated stripe test patterns and experimental image volumes of live LLC-PK1 cells with the light sheets studied here. Furthermore, in either modality, accurate, ghost-free reconstruction implies the ability of RL deconvolution to recover sample spatial frequencies even within deep OTF gaps, as surmised above. Since these results contradict the arguments in (*10*–*12*) that strong sidelobes lead to image artifacts, we also break down these arguments in note S9 and explain why they are not relevant for the light sheets considered here.

It should perhaps not be surprising that the fluorescence generated by the sidelobes of an LLS provides valuable high-resolution information rather than obscuring background, given the success of widefield 3D SIM (*13*). There, periodic interference patterns often extending throughout the entirety of whole cells create fuzzy raw images rife with ghost artifacts. However, after acquiring 15 such images per *z* plane at three different orientations and five equal phase steps within the lateral period of the interference pattern, overlapping specimen spatial frequencies in these images are separated, amplitude-corrected by deconvolution, and reassembled into a final image of ~2× resolution gain in all three dimensions. Accurate image reconstruction by RL deconvolution in LLSM is generally much easier, given the generally much tighter envelope bounding the sidelobes of a swept LLS.

This tighter bounding envelope allows LLSM to extend SIM to samples that are so large and/or densely fluorescent that the amount of out-of-focus emission is too large to enable accurate reconstruction by widefield SIM (*2*, *23*). Axially, the resolution (*R*_*z*_optical__)_max_ of LLS-SIM is identical to swept LLSM with the same light sheet: 316 nm in the case of a hexagonal LLS of NA_exc_ = 0.46 and σ_NA_ = 0.10 (fig. S20 and movie S10). This is 2.2× better than (*R*_*z*_optical__)_max_ of a widefield microscope at NA_exc_= 1.2 and slightly better than the 344-nm axial resolution at NA_exc_ = 1.2 of widefield 3D SIM. Laterally, however, the resolution $R({\hat{e}}_{x_{optical}}^{SI})=$ 183 nm is 1.4× better than in the swept mode with the same light sheet, and the harmonics of ${\mathrm{OTF}}_{\mathrm{exc}}^{\mathrm{fixed}}(k)$ of the hexagonal lattice (Fig. 1Cc) create copies of OTF_det_(***k***) in ${\mathrm{OTF}}_{\mathrm{overall}}^{\mathrm{SI}}(k)$ that fill the gaps in the swept OTF to result in a more uniform OTF throughout the extended support without the need for RL deconvolution. Although this comes at the cost of acquiring five phase-stepped raw images per plane, LLS-SIM is sufficiently rapid and gentle that we imaged a 80 × 194 × 18 μm^3^ field of living LLC-PK1 cells expressing an ER marker at 5.6 s per volume for 100 volumes with minimal photobleaching (movie S10), and the FFTs of the reconstructed image volume indicated the ability to recover sample information across most of the expanded support region (fig. S20, upper right inset, right panel).

#### Excitation envelope and photobleaching

Another concern expressed in (*10*–*12*) is that sidelobes to the excitation PSF lead to accelerated photobleaching and phototoxicity. In movie S11, the theoretical light sheet excitation cross-section (red) and cumulative intensity from the center of the light sheet (blue), normalized to the integrated intensity across the entire light sheet,

$$I_{\mathrm{cumulative}}^{\mathrm{swept}}(y,z)=\int_{-z}^{z}{\mathrm{PSF}}_{\mathrm{exc}}^{\mathrm{swept}}(y,z^{\prime }){dz}^{\prime }/\int_{-\infty }^{\infty }{\mathrm{PSF}}_{\mathrm{exc}}^{\mathrm{swept}}(y,z^{\prime }){dz}^{\prime }$$ (24)

is shown as a function of position *y* along the propagation axis for the Gaussian, sinc, and seven LLSs of common length *y*_FWHM_ ∼ 50λ_exc_/*n* in movies S8 and S9. At the excitation focus, the FWHM of $I_{\mathrm{cumulative}}^{\mathrm{swept}}(0,z)$ scales approximately with ${\left({\mathrm{NA}}_{\mathrm{exc}}^{\max}\right)}^{2}$ in most cases. Furthermore, at the edges of the propagation range, where ${\mathrm{PSF}}_{\mathrm{exc}}^{\mathrm{swept}}(|y_{\mathrm{HWHM}}|,0)$, the FWHM of $I_{\mathrm{cumulative}}^{\mathrm{swept}}(y_{\mathrm{HWHM}},z)$ approximately doubles, as expected by energy conservation. Thus, there is potentially a quadratically increasing cost in terms of photobleaching and phototoxicity at higher desired $R({\hat{e}}_{z_{\mathrm{optical}}})$, which should be addressed.

**Fig. 6.**
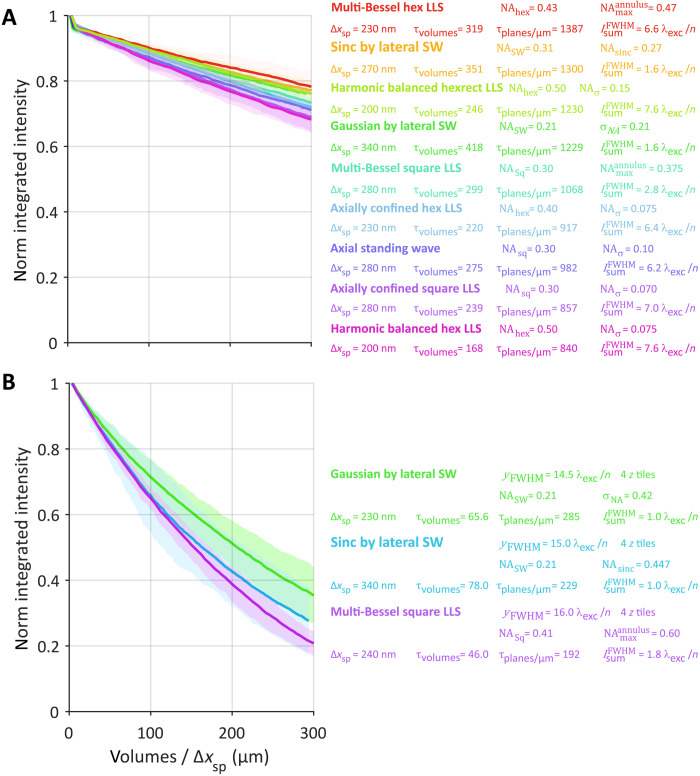
Comparative normalized bleaching rates determined by live 3D imaging of confluent human induced pluripotent stem cells. The cells are gene-edited for mono-allelic expression of mEGFP–α-tubulin. (**A**) Nine light sheets of Figs. 4 and 5, all of length *y*_FWHM_~ 50 λ_exc_/*n*. (**B**) Gaussian, sinc, and MB square LLSs, all of length *y*_FWHM_~15 λ_exc_/*n*, so chosen to have NA_exc_, and hence axial resolution, comparable to the hexagonal and hexagonal-rectangular light sheets in (A).

It is difficult to assess phototoxicity quantitatively and apply the findings broadly, as it depends on the following: cell type, state, density, passage number, and expression level; fluorophore type and delivery; environment past and present (e.g., temperature, pH, CO_2_, contamination, and substrate adhesion); and imaging wavelength, intensity, and total dose. Hence, we focus on the simpler problem of quantifying photobleaching across light sheets, since it appears less dependent on a number of these parameters. Specifically, as a reproducible standard, we use the photobleaching of living confluent human induced pluripotent stem cells (hiPSCs) gene-edited for mono-allelic expression of monomeric enhanced green fluorescent protein (mEGFP)–α-tubulin (fig. S21). For the Gaussian, sinc, and seven LLSs in Figs. 2 and 3, figs. S9B and S11 to 13, and movies S8, S9, and S11, all of length *y*_FWHM_ ∼ 50 λ_exc_/*n*, we imaged cells at an SNR ∼ 20, as measured at microtubules, for 100 volumes of 151 planes each at 2.1-s intervals. The step size ∆*x*_sp_ between planes varied to achieve Nyquist sampling for ${\mathrm{NA}}_{\mathrm{exc}}^{\max}$ of each light sheet (as given in Fig. 6A and table S1). We imaged six different fields of cells for each light sheet and fit a single exponential *I*(*n*_volume_) = *I_o_* exp(−*n*_volume_/τ_volume_) to the bleaching data from each session to estimate τ_volume_ (Fig. 6A) and its uncertainty (translucent band for each light sheet; Fig. 6A).

Expressed in terms of τ_volume_, the bleaching rate between the nine light sheets varied by ∼2.0×, with the Gaussian light sheet bleaching the slowest. However, these differences are far less than would be expected if the only role of excitation sidelobes was to create out-of-focus haze that accelerates photobleaching: After all, the integrated intensity $I_{\mathrm{cumulative}}^{\mathrm{swept}}(0,z_{\mathrm{HWHM}}^{\mathrm{center}})$ across the FWHM of the central excitation peak in the Gaussian and sinc light sheets was 80 and 70% of the total, whereas $I_{\mathrm{cumulative}}^{\mathrm{swept}}(0,z_{\mathrm{HWHM}}^{\mathrm{center}})$ was only 12 to 18% for the seven LLSs in movie S11. These numbers provide additional evidence that LLS sidelobes provide useful signal. Furthermore, τ_volume_ does not take into account that the Gaussian and sinc light sheets move in coarser steps (∆*x*_sp_ = 340 and 270 nm, respectively) by virtue of their lower ${\mathrm{NA}}_{\mathrm{exc}}^{\max}$, and therefore, the signal they produce at the 151 planes/volume used here comes from a larger volume having a correspondingly larger photon budget than the LLS. Once the bleaching rate is normalized by τ_planes/μm_ = τ_volume_/∆*x*_sp_ to account for the extra information per unit length of FOV produced by light sheets of higher ${\mathrm{NA}}_{\mathrm{exc}}^{\max}$ (or, equivalently, the greater number of voxels in an image volume of fixed size), the bleaching rates of all seven LLSs in Fig. 6 are to within ∼30% of that in the Gaussian and sinc cases. Thus, to close order, all these light sheets are equally efficient in converting fluorescent photons into useful signal. This is consistent with the successful reassignment of sidelobe fluorescence to its correct origins after RL deconvolution at comparable SNR seen for all LLSs in LLC-PK1 cells (e.g., Fig. 5B) as well as the hiPSCs used for the bleaching measurements here (fig. S21).

Rather than using an LLS to image across a long FOV in the propagation direction ${\hat{e}}_{y_{\mathrm{optical}}}$ at high ${\mathrm{NA}}_{\mathrm{exc}}^{\max}$, an alternative is to scan a Gaussian or sinc light sheet of comparably high ${\mathrm{NA}}_{\mathrm{exc}}^{\max}$ but shorter length *y*_FWHM_ across a comparable FOV in the ${\hat{e}}_{y_{\mathrm{optical}}}$ direction at each image plane (*34*). To eliminate the collection of out-of-focus fluorescence from parts of the light sheet outside the ∣*y*∣ ≤ *y*_HWHM_ *z*-confined propagation portion but inside the *y* FOV (e.g., gold arrow in fig. S22), the camera integration window moves with the confined portion as the light sheet is scanned. We evaluated the imaging performance of short light sheets such as these by imaging live ER-labeled LLC-PK1 cells over the same ~50λ_exc_/*n* FOV in the propagation direction as used in the examples above, but with Gaussian (fig. S22 and movie S12), sinc (fig. S23 and movie S13), and MB square LLSs (fig. S10 and movie S4) of length ~15λ_exc_/*n*. Because we were not equipped to rapidly scan these light sheets across the *y* FOV, we instead imaged the cells with four tiles stacked in the ${\hat{e}}_{z_{\mathrm{specimen}}}$ direction, which gave the small overlap between tiles needed to successfully stitch the data into a single image volume. The integration time for each single tile frame was set to one-fourth that used for the longer light sheets used elsewhere here to achieve a total signal integration time over the entire volume comparable to that used with the longer light sheets, although the overhead associated with the additional scan steps and tiling resulted in total imaging times ~4.0× slower.

As seen in panels I to L of figs. S10, S22, and S23, the agreement between the theoretical and experimental overall OTFs at both the focal plane and *y* = *y*_HWHM_ is good for all three light sheets. In addition, all three were able to recover sample spatial frequencies (FFT insets, panels Q) up to the boundary of their *k_z_* support, corresponding to $R({\hat{e}}_{z_{\mathrm{optical}}})=$ 581, 546, and 407 nm for the Gaussian, sinc, and MB square cases of ${\mathrm{NA}}_{\mathrm{exc}}^{\max}=$ 0.42, 0.45, and 0.60, respectively. However, unlike the nine light sheets of length *y*_FWHM_ ∼ 50λ_exc_/*n* in Fig. 6A, all three short light sheets in Fig. 6B induced photobleaching in hiPSCs endogenously expressing mEGFP–α-tubulin substantially faster than the reference long Gaussian light sheet of fig. S3, with $\tau_{\mathrm{planes}/\mu m}/\tau_{\mathrm{planes}/\mu m}^{\mathrm{ref}}=$ 4.3, 5.4, and 6.4, respectively.

The reason for faster bleaching with these shorter light sheets is clear: For all light sheets studied here, both long and short, nearly all the fluorescence generated within the region ∣*y*∣ ≤ *y*_HWHM_ is collected and converted to useful signal, including that produced by any significant sidelobes, at SNR levels of 20 to 30, consistent with long-term 3D live cell imaging (fig. S24A, blue regions). However, if the specimen is longer than *y*_FWHM_ in the ${\hat{e}}_{y_{\mathrm{optical}}}$ direction, fluorescence is also generated beyond ∣*y*∣ = *y*_HWHM_ that is increasingly out-of-focus and information poor (fig. S24B, red regions). This background obscures the in-focus signal (fig. S24B, blue region) as the light sheet is scanned in ${\hat{e}}_{y_{\mathrm{optical}}}$ to cover a larger $y_{\mathrm{optical}}^{\mathrm{FOV}}$ unless a sliding camera integration window of width *y*_FWHM_ is used to reject it.

By this argument, any of the light sheets studied here should photobleach a specimen of size $y_{\mathrm{optical}}^{\mathrm{FOV}}>y_{\mathrm{FWHM}}$ at a rate τ_planes/μm_ ∝ 1/*y*_FWHM_. Given the four tiles used for the short light sheets in Fig. 6B, this is consistent with the 4.3× faster bleaching seen for the short Gaussian light sheet (fig. S22) versus the long one (fig. S3). However, the photobleaching rate increases further with increasing ${\mathrm{NA}}_{\mathrm{exc}}^{\max}$ for the short sinc and MB square light sheets. This is consistent with (*2*) and (*23*), where it was determined that photobleaching increases nonlinearly with increasing peak intensity in the specimen. The LLSs studied here are particularly advantageous in this regard, because by spreading the excitation across multiple planes simultaneously (the fluorescence from all that contribute useful signal), the intensity in the central peak is kept lower for the same SNR than would be the case even if it were possible to produce a sidelobe-free light sheet of the same central peak width and propagation length. Furthermore, it has been shown that live specimens often exhibit phototoxic effects long before substantial photobleaching is evident [e.g., movie S3 of (*2*); figure 3 (H and I) of (*20*)], so the relative noninvasiveness of LLSs compared to axially scanned confined beams of similar ${\mathrm{NA}}_{\mathrm{exc}}^{\max}$ might be expected to be even more pronounced.

The above results demonstrate that LLSs of all four symmetries in Fig. 1 can experimentally achieve resolution $R({\hat{e}}_{z_{\mathrm{optical}}})$ well in excess of that possible with Gaussian and sinc light sheets of similar length and are completely consistent with the expectations of theoretical models. Furthermore, the out-of-focus fluorescence these light sheets generate can be efficiently reassigned by RL deconvolution to their original sources to achieve accurate, background-free, high-resolution reconstructions of sample structure without accelerating photobleaching beyond that observed with low-resolution Gaussian beams of similar length. Consequently, as has been shown [e.g., (*2*–*9*)], LLSM is well suited to reveal novel 3D biological processes noninvasively at high resolution in both space and time. Our introduction here of the hexrect pattern and HB LLSs further improves their performance and expands their potential range of applicability, particularly at higher resolution (i.e., higher NA_exc_) and/or over larger fields of view (i.e., longer *y*_FWHM_).

## MATERIALS AND METHODS

### Light sheet instrumentation

The light sheets were experimentally characterized using an adaptive optical LLS microscope similar to one previously described (*7*). Briefly, a 488-nm laser (500 mW, MPB Communications Inc., 2RU-VFL-P-500-488-B1R) was expanded to 1/e^2^ diameter of 2.0 mm and passed onto an acousto-optic tunable filter (AOTF; Quanta-Tech, AA Opto Electronic, AOTFnC-400.650-CPCh-TN). The collimated beam was fanned out to uniformly expand in the *x*_optical_ axis using a Powell lens (Laserline Optics Canada, LOCP-8.9R20-2.0). The *z*_optical_ axis was expanded using a pair of 50- and 250-mm cylindrical lenses (25 mm diameter; Thorlabs, ACY254-050, LJ1267RM-A). The expanded beam illuminated a horizontal stipe on a grayscale SLM (Meadowlark Optics, AVR Optics, AVR17-0105). The light diffracted by the SLM was focused onto a mask containing user-selected annuli of numerous sizes (Thorlabs Imaging) to block unwanted DC and higher diffraction orders. The light passing through the chosen annulus was reflected off a pair of galvanometer mirrors (Cambridge Technology, Novanta Photonics, 6SD11226 and 6SD11587), which were conjugated to the back pupil of the EO (Thorlabs, TL20X-MPL) and used to scan along the *x*_optical_ and *z*_optical_ axes. An additional custom mask was placed near the back pupil of EO when an appropriate annulus was not available in the standard annular mask. The fluorescence generated by the specimen was collected through the DO (Zeiss, 20×, 1.0 NA, 1.8-mm free working distance (FWD), 421452-9800-000), projected onto a pupil-conjugate deformable mirror (DM; ALPAO, DM69) and imaged onto a scientific complementary metal-oxide semiconductor (sCMOS) camera (Hamamatsu ORCA Fusion). The correction of system aberration in the excitation and detection light path is discussed in notes S10 and S11.

### Sample preparation

The coverslips (Thorlabs, CG15XH) used for imaging were cleaned by sonicating in 70% ethanol and Milli-Q water for a minimum of 30 min each and stored in Milli-Q water. Before use, the coverslips were air-dried and plasma-treated (Harrick Plasma, PDC-32G) for 45 to 60 s at a maximum radio frequency power of 18 W under a vacuum pressure of 0.20 torr. We deposited 0.10 ml of 0.1% poly-d-lysine (PDL; Sigma-Aldrich, P0899) to cover the coverslip surface immediately after plasma treatment. The PDL was allowed to air-dry, subsequently rinsed with Milli-Q water, and finally deposited 200-nm-diameter fluorescent beads (Invitrogen FluoSpheres Carboxylate-Modified Microspheres, 505/515 nm, F8811) to achieve a density of ~1 bead per 100 × 100 μm^2^ imaged area. Pig kidney epithelial cells (LLC-PK1) were a gift from M. Davidson at Florida State University. The mono-allelic mEGFP-tagged TUBA1B WTC iPSCs, AICS-0012 cl.105, were purchased through Coriell and developed at the Allen Institute for Cell Science (allencell.org/cell-catalog). The LLC-PK1 cells were grown in Dulbecco’s modified Eagle’s medium (DMEM) with GlutaMAX (Gibco, 10566016) supplemented with 10% fetal bovine serum (FBS; Avantor Seradigm). The iPSC line was grown in StemFlex (Gibco, A3349401) on Matrigel-coated (Corning, 354230) plates. Matrigel was diluted with DMEM/F12 without phenol red (Gibco, 11039021) at a 1:30 ratio. We deposited 1.0 ml of diluted Matrigel to each well of a six-well plate and incubated at 25°C for 2 hours before use. Both cell lines were cultured under standard conditions (37°C, 5% CO_2_, 100% humidity) with weekly passaging. For LLC-PK1 imaging, the cells were plated on the bead-coated 25-mm coverslips and imaged between 30 and 80% confluency. The LLC-PK1 cells were imaged at 37°C in Leibovitz’s L-15 medium without phenol red (Gibco, 21083027), with 5% FBS (American Type Culture Collection, SCRR-30-2020), and an antibiotic cocktail containing 0.1% ampicillin (Thermo Fisher Scientific, 611770250), 0.1% kanamycin (Thermo Fisher Scientific, 11815024), and 0.1% penicillin/streptomycin (Thermo Fisher Scientific, 15070063). To plate hiPSCs on the bead-coated coverslips, we were further treated with Matrigel as described above. The hiPSCs were imaged at 37°C with 16% O_2_ and 5% CO_2_ in StemFlex supplement with DMEM/F12 medium without phenol red (Thermo Fisher Scientific, 21041025).

### Experimental light sheet characterization using fluorescent beads

To characterize different light sheets, 200-nm-diameter fluorescent beads plated on a 25-mm coverslip were imaged in ~45 ml of medium at 37°C. The cross-sectional excitation light sheet profile xzPSF was measured by placing a bead at the focus of the light sheet. The light sheet was then scanned in 100-nm steps over 10 × 10 μm^2^ with the *x* and *z* galvos, while the integrated bead fluorescence at each pixel was recorded. The 3D overall PSF was measured at various locations *y* along the propagation direction by the coordinated movement of two specimen stages (SmarAct MLS-3252-S and SLS-5252-S for the *x*_sp_ and *z*_sp_ axes, respectively) to translate the bead along the DO axis (*z*_optical_) while recording an image of the bead every 100 nm over a 15-μm range. Autofocus using fluorescent beads was performed before each measurement to ensure that the light sheet was correctly centered on the focal plane of DO at the start of each *z* scan as described previously (*7*).

### Experimental light sheet characterization using live cells

To characterize different light sheets, we imaged LLC-PK1 cells stably expressing the ER marker mEmerald-Calnexin. Using these cells, the resolution of each light sheet was obtained as shown in panels P and Q of Figs. 2B and 3 and figs. S3, S4, S9B, S10 to S13, S17, S22, S23, and S25 at SNR ~ 30 by scanning the sample stage at constant velocity and acquiring one *xy*_optical_ image every 20 ms, with the speed set such that the sample traversed a distance Δ*x*_sp_ as given in Fig. 6 for each light sheet in this time. To characterize the performance and photobleaching rate of each light sheet when imaging the 3D dynamics of living cells, 100 image volumes were collected at 3- to 4-s intervals at a lower SNR ~ 20 and a higher speed of ~2 ms per plane for 1000 planes per volume (table S2). The cell data were deconvolved using PSFs acquired under identical conditions as described below, and then deskewed and rotated to display the volumes in specimen coordinates. The photobleaching measurements and deconvolution of experimental data are discussed in notes S12 and S13, respectively.

### Generation and processing of simulated stripe patterns

We generated the raw stripe pattern volumes by convolving the ground truth stripe pattern with the theoretical ${\mathrm{PSF}}_{\mathrm{overall}}^{\mathrm{swept}}(x)$using a pixel size of 0.10 medium wavelengths (corresponding to 36.7 nm for 488 nm in water). First, we simulated the 28 ground truth stripes as a binary image within a 3D volume (1001 × 1001 × 1001 pixel^3^), where each stripe was centered along *y*_optical_ with 799 pixels in *x*_optical_ and 1 pixel in *z*_optical_. The first 26 successive stripes were positioned by linearly spaced increments from 1 to 25 pixels, with the 27th and 28th stripes incremented 100 and 151 pixels, respectively. Second, we simulated ${\mathrm{PSF}}_{\mathrm{overall}}^{\mathrm{swept}}(x)$ for each light sheet by the product ${\mathrm{PSF}}_{\mathrm{exc}}^{\mathrm{swept}}(x)\cdot {\mathrm{PSF}}_{\det}(x)$, where ${\mathrm{PSF}}_{\mathrm{exc}}^{\mathrm{swept}}(x)$ was simulated on the basis of the corresponding light sheet parameters, and PSF_det_(***x***) was simulated with the model of Aguet (*35*) and Richards and Wolf (*36*), using the program PSF Generator from http://bigwww.epfl.ch/algorithms/psfgenerator. Third, the convolved volumes were downsampled to achieve a pixel size of 0.108 μm, normalized by its 99.9 percentile, and multiplied by 400 (for SNR = 20 presented in this paper). Poisson noise was then added, along with a camera background offset of 100 counts to match the experimental data. The Poisson noise was approximated by the pixel-dependent variance Gaussian distribution *N*(0, *I_i_*), where *I_i_* is the pixel intensity for pixel *i*; the Gaussian noise follows *N*(0,4^2^), where 4 is the SD of the shuttered camera images.

We used RL deconvolution as described above to deconvolve the simulated raw stripe pattern volumes using ${\mathrm{PSF}}_{\mathrm{overall}}^{\mathrm{swept}}(x)$ downsampled to the experimental pixel size of 0.108 μm. Since the results are uniform along *x*_optical_, we determined the optimal RL iterations by calculating the FSC on a single cropped subvolume containing all 28 stripes.

Additional details pertaining to LLS-SIM reconstruction, stitching tiled subvolumes, and data visualization are discussed in notes S14 to S16. MATLAB source code used for light sheet simulations can be accessed on GitHub (https://github.com/abcucberkeley/LLS_Simulation).

## Supplementary Materials

### This PDF file includes:

Supplementary Text Figs. S1 to S27 Tables S1 and S2 Legends for movies S1 to S18 References
