# Supplementary material for: Characterization, comparison, and optimization of lattice light sheets
Source: Sci Adv. 2023 Mar 31;9(13):eade6623. doi: 10.1126/sciadv.ade6623 (PMC10065451; doi:10.1126/sciadv.ade6623)
Supplement: Supplementary file 1 — Supplementary Text Figs. S1 to S27 Tables S1 and S2 Legends for movies S1 to S18 References [file sciadv.ade6623_sm.pdf]

Science Advances
AAAS

Supplementary Materials for

Characterization, Comparison, and Optimization of Lattice Light Sheets

Gaoxiang Liu *et al.*

Corresponding author: Eric Betzig, betzige@janelia.hhmi.org; Srigokul Upadhyayula, sup@berkeley.edu

Sci. Adv. **9**, eade6623 (2023)
DOI: 10.1126/sciadv.ade6623

The PDF file includes:

Supplementary Text
Figs. S1 to S27
Tables S1 and S2
Legends for movies S1 to S18
References

Other Supplementary Material for this manuscript includes the following:

Movies S1 to S18

Supplementary Text

S1. Methods of light sheet microscopy using a laterally confined beam

A. Swept beam light sheet microscopy

When a beam of excitation profile $PSF_{exc}^{fixed}(\boldsymbol{x})$ confined in two dimensions perpendicular to its direction of propagation y is swept continuously in $x \perp y, z$ across a desired FOV large compared to its confinement in x:

$$PSF_{exc}^{swept}(\boldsymbol{x}) = \int_0^{FOV} PSF_{exc}^{fixed}(x - x', y, z)dx' \approx \int_{-\infty}^{\infty} PSF_{exc}^{fixed}(x - x', y, z)dx' = \iiint_{-\infty}^{\infty} PSF_{exc}^{fixed}(x - x', y, z)\delta(y')\delta(z')\boldsymbol{dx}' = PSF_{exc}^{fixed}(\boldsymbol{x}) \otimes [\delta(y)\delta(z)] \quad (S1a)$$

which is the convolution of the stationary beam with an infinite line along the x axis. Thus, $PSF_{exc}^{swept}(\boldsymbol{x})$ is constant along x and confined in z. By the convolution theorem, the corresponding transverse cross-sectional OTF at any position y is then given by:

$$OTF_{exc}^{swept}(k_x, y, k_z) = FT_{xz}\{PSF_{exc}^{swept}(\boldsymbol{x})\} \cdot \delta(k_x) = OTF_{exc}^{fixed}(0, y, k_z) \quad (S1b)$$

This result is intuitively clear: the amplitudes of any spatial frequencies of nonzero k_x in OTF_{exc}^{fixed} will be averaged out by the sweep operation, whereas purely axial and/or longitudinal spatial frequencies are unaffected by a lateral sweep. Eq. (S1b) is also true for any periodic excitation pattern $PSF_{exc}^{fixed}(x + T, y, z) = PSF_{exc}^{fixed}(\boldsymbol{x})$ that is swept at constant velocity over an integral multiple of T during the acquisition of each frame, such as with a triangle wave pattern ("dithered").

Combining Eq. (3) with Eq. (S1b) and explicitly writing out the convolution in the former yields the generalized swept beam OTF in terms of the pupil electric field:

$$OTF_{exc}^{swept}(y, k_z) = OTF_{exc}^{fixed}(0, y, k_z)$$
$$\propto \int_{-NA_{exc}k_o}^{NA_{exc}k_o} dk_x' \left\{ \int_{-\infty}^{\infty} dk_z' E_{pupil}(k_x', k_z')\overline{E_{pupil}(k_x', k_z - k_z')} \exp[i\mathrm{K}(k_x', k_z', k_z)y] \right\} \quad (S2a)$$

where:

$$\mathrm{K}(k_x', k_z', k_z) = \left(\sqrt{k^2 - k_x'^2 - k_z'^2} - \sqrt{k^2 - k_x'^2 - (k_z - k_z')^2}\right) \quad (S2b)$$

$OTF_{exc}^{swept}(y, k_z)$ is therefore the 1D auto-correlation in k_z of the 2D generalized pupil function $E_{pupil}(k_x, k_z)\exp(ik_y y)$. It is equivalent to the incoherent sum (or integral) of the 1D autocorrelations in k_z of every 1D column of different k_x in the generalized pupil function. This result, which is graphically depicted in Fig. S1, is known as the field synthesis theorem (*11*).

The ultimate performance of a light sheet microscope is determined by its overall PSF and corresponding OTF. These in turn depend on the interplay of the light sheet's excitation PSF with $PSF_{det}(\boldsymbol{x})$, the ideal diffraction-limited PSF of a detection objective of numerical aperture NA_{det}. For a swept light sheet:

$$PSF_{overall}^{swept}(\boldsymbol{x}) = PSF_{exc}^{swept}(y,z) \cdot PSF_{det}(\boldsymbol{x}) \qquad (S3a)$$

$$OTF_{overall}^{swept}(y,\boldsymbol{k}) = OTF_{exc}^{swept}(y,k_z) \otimes_{k_z} OTF_{det}(\boldsymbol{k}) \quad \text{where:} \qquad (S3b)$$

$$OTF_{det}(\boldsymbol{k}) = FT_{k_x k_y k_z}\{PSF_{det}(\boldsymbol{x})\} \qquad (S3c)$$

For our theoretical calculations of light sheet performance, we calculated $PSF_{det}(\boldsymbol{x})$ over a 3D volume of $(\pm 50\,\lambda_{exc}/n)^3$ sufficiently large to encompass all excitation sidelobes of any of the light sheets we studied, which is necessary for an accurate representation of $OTF_{overall}^{swept}(y,\boldsymbol{k})$. Notably, by Eq (S3c) the size of this volume also determines the scaling of $OTF_{det}(\boldsymbol{k})$ at all $\boldsymbol{k} \neq 0$ relative to the DC peak, which itself becomes singular as the volume → ∞.

B. Confocal light sheet microscopy

When a confined beam $PSF_{exc}^{fixed}(\boldsymbol{x})$ created with a maximum numerical aperture of excitation NA_{exc} is moved discretely in steps $\Delta x \sim \lambda_{exc}/(4NA_{exc})$ across the FOV rather than continuously as above, and the fluorescence emission at each step is recorded at a camera conjugate to $PSF_{exc}^{fixed}(\boldsymbol{x})$ by a slit $(2N_{x'}+1)$ pixels wide centered at x, an optically sectioned confocal PSF $PSF_{overall}^{conf}(\boldsymbol{x})$ is obtained by summing the recorded emission over all pixels in x':

$$PSF_{overall}^{conf}(\boldsymbol{x}) = PSF_{exc}^{fixed}(\boldsymbol{x}) \cdot \sum_{n=-N_{x'}}^{N_{x'}} PSF_{det}(x - n\Delta x', y, z) \qquad (S4a)$$

where $\Delta x'$ is the pixel width. The effective detection PSF is therefore the discrete 1D convolution of the ideal PSF with the width of the pixel band:

$$PSF_{overall}^{conf}(\boldsymbol{x}) = PSF_{exc}^{fixed}(\boldsymbol{x}) \cdot PSF_{det}^{conf}(\boldsymbol{x}) \qquad (S4b)$$

where:

$$PSF_{det}^{conf}(\boldsymbol{x}) = PSF_{det}(\boldsymbol{x}) \otimes_x \left\{ rect\left[\frac{x}{(2N_{x'}+1)\Delta x'}\right]\right\} \qquad (S4c)$$

and $\text{rect}(x) = 1$ for $|x| < \frac{1}{2}, \frac{1}{2}$ for $x = \frac{1}{2}, 0$ for $|x| > \frac{1}{2}$. By the convolution theorem, the corresponding OTF at a given position y is given by:

$$\begin{aligned} OTF_{overall}^{conf}(y,\boldsymbol{k}) &= OTF_{exc}^{fixed}(k_x,y,k_z) \otimes_{k_x k_z} OTF_{det}^{conf}(\boldsymbol{k}) \\ &\propto OTF_{exc}^{fixed}(k_x,y,k_z) \otimes_{k_x k_z} \{OTF_{det}(\boldsymbol{k}) \cdot \text{sinc}[(2N_{x'}+1)k_x\Delta x'/2]\} \qquad (S4d) \end{aligned}$$

where $\text{sinc}(x) = \sin(x)/x$.

C. Incoherent structured illumination light sheet microscopy

When $PSF_{exc}^{fixed}(\boldsymbol{x})$ is moved in x in discrete steps of period $\mathrm{T} > \lambda_{exc}/(2NA_{exc})$ over a FOV large compared to its extent, the excitation PSF of the resulting structured light sheet can be approximated by:

$$PSF_{exc}^{iSI}(\boldsymbol{x}) = \sum_{j=-N}^{N} PSF_{exc}^{fixed}(x - j\mathrm{T}, y, z) \approx \sum_{j=-\infty}^{\infty} PSF_{exc}^{fixed}(x - j\mathrm{T}, y, z)$$

$$= \iiint_{-\infty}^{\infty} \sum_{j=-\infty}^{\infty} PSF_{exc}^{fixed}(x - x', y, z)\, \delta(x - j\mathrm{T})\delta(y')\delta(z')\boldsymbol{dx'} \qquad (S5a)$$

$$= PSF_{exc}^{fixed}(\boldsymbol{x}) \otimes \sum_{j=-\infty}^{\infty} \delta(x - j\mathrm{T})\delta(y)\,\delta(z)$$

In other words, the PSF of the stepped light sheet is the convolution of the stationary PSF with an infinite series of delta functions of period T along the x axis. By the convolution theorem, the corresponding excitation OTF is then given by:

$$OTF_{exc}^{iSI}(k_x, y, k_z) = OTF_{exc}^{fixed}(k_x, y, k_z) \sum_{j=-\infty}^{\infty} \delta\left(k_x - \frac{2\pi j}{\mathrm{T}}\right) = \sum_{j=-J}^{J} OTF_{exc}^{fixed}\left(\frac{2\pi j}{\mathrm{T}}, y, k_z\right) \quad (S5b)$$

where J is the largest integer for which $J < 2NA_{exc}\mathrm{T}/\lambda_{exc}$, since higher values of J correspond to spatial frequencies $\boldsymbol{k}$ beyond the theoretical resolution limits defined by $\left|OTF_{exc}^{fixed}(\boldsymbol{k})\right| > 0$ (i.e., the "support"). Indeed, for $J > 2NA_{exc}\mathrm{T}/\lambda_{exc}$, Eq. (S5b) reduces to Eq. (S1b), because the stepped copies of $PSF_{exc}^{fixed}(\mathbf{x})$ are then no longer mutually resolvable, and the stepped light sheet becomes continuous.

A single fluorescence image collected with $PSF_{exc}^{iSI}(\boldsymbol{x})$ contains information about the specimen out to $k_x^{SImax} = \pm 4\pi(NA_{exc}/\lambda_{exc} + NA_{det}/\lambda_{det})$ down shifted by the $2J + 1$ bands in Eq. (S5b) to overlap within the $k_x^{detmax} = \pm 4\pi NA_{det}/\lambda_{det}$ passband of the detection objective. Using the principles of structured illumination microscopy (SIM(*13*)), this information can be reassigned to its correct location in an expanded frequency space representation of the specimen by: a) acquiring $2J + 1$ raw images with $PSF_{exc}^{SI}(\boldsymbol{x})$ successively shifted by $\Delta x = \mathrm{T}/(2J + 1)$ between each; b) separating the overlapped frequency components in these images by matrix inversion; c) assigning them to their correct locations in k_x; and d) precisely stitching them together in amplitude and phase across the extended support by cross-correlation. The net result is a light sheet image with resolution in x extended from k_x^{detmax} to k_x^{SImax}.

Prior to deconvolution, the strength of any frequency shifted copy of information in any reconstructed SIM image is proportional to the strength of the excitation harmonic responsible for the shift. Thus, the effective overall OTF for incoherent light sheet SIM is:

$$OTF_{overal}^{iSI}(y, \boldsymbol{k}) = OTF_{exc}^{iSI}(k_x, y, k_z) \otimes_{k_x k_z} OTF_{det}(\boldsymbol{k}) \qquad (S6)$$

D. Coherent structured illumination light sheet microscopy

A periodic coherent structured light sheet can be created by writing (e.g., with a spatial light modulator (SLM)) a periodic array in x of $E_{exc}^{fixed}(x, 0, z)$ from Eqs. (1) at a plane conjugate to the focal plane of the excitation objective. Each array element j laterally displaced a distance $x = j\mathrm{T}$ when projected to the focal plane arises from a pupil field given by:

$$E_{pupil}^{j\mathrm{T}}(x_p, z_p) = E_{pupil}^{0}(x_p, z_p) \cdot exp\left[i2\pi\left(\frac{j\mathrm{T}}{\lambda_{exc}F}\right)x_p\right] \qquad (S7a)$$

where $E_{pupil}^{0}(x_p, z_p)$ is the pupil field that gives rise to the centered ($x = 0$) copy $E_{exc}^{fixed}(\boldsymbol{x})$ from above, and F is the focal length of the objective. The total electric field at the rear pupil $E_{pupil}^{\mathrm{cSI}}(x_p, z_p)$ for an infinite linear array of such beams is then given by the superposition of their individual pupil fields $E_{pupil}^{j\mathrm{T}}(x_p, z_p)$:

$$E_{pupil}^{\mathrm{cSI}}(x_p, z_p) = \sum_{j=-\infty}^{\infty} E_{pupil}^{j\mathrm{T}}(x_p, z_p) = E_{pupil}^{0}(x_p, z_p) \cdot \sum_{j=-\infty}^{\infty} exp\left[i2\pi\left(\frac{j\mathrm{T}}{\lambda_{exc}F}\right)x_p\right] =$$
$$E_{pupil}^{0}(x_p, z_p) \cdot \frac{\lambda_{exc}F}{\mathrm{T}} \sum_{m=-\infty}^{\infty} \delta\left(x_p - m\frac{\lambda_{exc}F}{\mathrm{T}}\right) \qquad (S7b)$$

where the identity $\sum_{-\infty}^{\infty} exp(i2\pi jx/\beta) = \beta \sum_{-\infty}^{\infty} \delta(x - m\beta)$ was used in the lower line of Eq. (S7b). Since $E_{pupil}^{0}(x_p, z_p) = 0$ for all $|x_p| > NA_{exc}F$ outside of the pupil, Eq. (S7b) reduces to a finite sum:

$$E_{pupil}^{\mathrm{cSI}}(x_p, z_p) \propto \sum_{m=-M}^{M} E_{pupil}^{0}\left(m\frac{\lambda_{exc}F}{\mathrm{T}}, z_p\right) \qquad (S7c)$$

where M is the largest integer for which $M < NA_{exc}\mathrm{T}/\lambda_{exc}$. In other words, the electric field in the rear pupil required to produce a light sheet consisting of a linear array of coherent beams of period T consists of a periodic series of $2M + 1$ lines parallel to the z_p axis.

Combining Eqs. (1d) and (S7c), the electric field of the coherent structured light sheet is given by:

$$E_{exc}^{cSI}(\boldsymbol{x}) \propto \sum_{m=-M}^{M} FT_{k_x k_z}^{-1}\left[E_{pupil}^{0}\left(\frac{2\pi m}{\mathrm{T}}, k_z\right)\exp(ik_y y)\right] \qquad (S8a)$$

Eq. (2a) then gives the corresponding excitation PSF:

$$PSF_{exc}^{cSI}(\boldsymbol{x}) \propto \sum_{m=-M}^{M} \sum_{m'=-M}^{M} FT_{k_x k_z}^{-1}\left[E_{pupil}^{0}\left(\frac{2\pi m}{\mathrm{T}}, k_z\right)\exp(ik_y y)\right]$$
$$\cdot \overline{FT_{k_x k_z}^{-1}\left[E_{pupil}^{0}\left(\frac{2\pi m'}{\mathrm{T}}, k_z\right)\exp(ik_y y)\right]} \qquad (S8b)$$

and Eq. (3) plus Eq. (S7c) give the corresponding excitation OTF:

$$OTF_{exc}^{cSI}(k_x, y, k_z) \propto \sum_{m=-M}^{M} \sum_{m'=-M}^{M} \left\{E_{pupil}^0\left(\frac{2\pi m}{\mathrm{T}}, k_z\right)\exp(ik_y y)\right\} \otimes_{k_x k_z} \overline{\left\{E_{pupil}^0\left(\frac{2\pi m'}{\mathrm{T}}, k_z\right)\exp(ik_y y)\right\}} \quad (S8c)$$

Thus, the excitation OTF of the coherent structured light sheet consists of $4M + 1$ equally spaced discrete harmonics in k_x. As in the incoherent case in supplementary note S1C, these down shift specimen information extending out to $k_x^{SImax} = \pm 4\pi(NA_{exc}/\lambda_{exc} + NA_{det}/\lambda_{det})$ to the detection passband, and by acquiring $4M + 1$ raw images with $PSF_{exc}^{SI}(\boldsymbol{x})$ successively shifted by $\Delta x = \mathrm{T}/(4M + 1)$ between each image, the principles of image reconstruction from 3D SIM can be applied to create a light sheet image with resolution in x extended from k_x^{detmax} to k_x^{SImax}. However, whereas the strengths of the incoherent harmonics are dictated by the autocorrelation over all points in the pupil field $E_{pupil}^0(k_x, k_z)$ giving rise to the beam that is stepped (Eqs. (3) and (S5b)), the strengths of the coherent harmonics are dictated by the autocorrelation of the much smaller subset of points in the pupil field where $k_x = \frac{2\pi m}{\mathrm{T}}$ for $-M < m < M$ (Eq. S8c). Since each additional point in an autocorrelation adds to the DC total, the nonzero incoherent harmonics are generally much weaker than the harmonic ones. Hence, effective overall OTF for coherent structured light sheet microscopy:

$$OTF_{overal}^{cSI}(y, \boldsymbol{k}) = OTF_{exc}^{cSI}(k_x, y, k_z) \otimes_{k_x k_z} OTF_{det}(\boldsymbol{k}) \quad (S9)$$

is generally much stronger than the incoherent one (Eq. (S6)).

S2. Theoretical resolution limits

The theoretical resolution limit of a linear optical microscope is defined by the support of:

$$OTF_{overall}(\boldsymbol{k}) = OTF_{exc}(\boldsymbol{k}) \otimes OTF_{det}(\boldsymbol{k}) \quad (S9a)$$

This support is a 2D surface in 3D space. The theoretical resolution $R(\hat{\boldsymbol{e}}_{\boldsymbol{k}})$ in any particular direction $\hat{\boldsymbol{e}}_k$ is determined by the magnitude k of the vector $\boldsymbol{k} = k\hat{\boldsymbol{e}}_k$ from the origin of $OTF_{overall}(\boldsymbol{k})$ to this surface. There are several directions $\hat{\boldsymbol{e}}_{\boldsymbol{k}}$ of physical interest for light sheet microscopy (Fig. S2). First, because all light sheets are designed to vary slowly in the propagation direction $\hat{\boldsymbol{e}}_{y_{optical}}$ defined by the optical axis of the excitation objective,

$$OTF_{exc}(\boldsymbol{k}) \sim \delta(k_y) OTF_{exc}(k_x, k_z) \quad (S9b)$$

Hence, resolution they provide along $\hat{\boldsymbol{e}}_{y_{optical}}$ is dictated primarily by the lateral support of the detection objective:

$$R\left(\hat{\boldsymbol{e}}_{y_{optical}}\right) = \frac{2\pi}{k_{y_{optical}}} = \frac{\lambda_{det}}{2NA_{det}} \quad (S10a)$$

Second, for all four ways above in which $PSF_{exc}^{fixed}(\boldsymbol{x})$ can be moved to create a light sheet, the resolution in the direction $\hat{\boldsymbol{e}}_{z_{optical}}$ defined by the optical axis z of the detection objective is:

$$R\left(\hat{\boldsymbol{e}}_{z_{optical}}\right) = \frac{2\pi}{k_{z_{optical}}} = \frac{\lambda_{exc}}{2NA_{exc}} \qquad (S10b)$$

since this is the location in $OTF_{overall}(\boldsymbol{k})$ where the center of $OTF_{det}(\boldsymbol{k})$ is furthest shifted axially by its convolution with $OTF_{exc}(\boldsymbol{k})$. For an ideal optical lattice, $NA_{exc} = NA_{lattice}$, the latter being the NA on which its B discrete illumination points are located (white dots, Figs. 1Aa, 1Ba, 1Ca, and 1Da). However, for a lattice light sheet, these points are stretched into lines of effective length $(\Delta k_z)_b$ along k_z, so that $NA_{exc} = \sim NA_{lattice} + (\Delta k_z)_b/2$. Third, the highest overall axial resolution is not along $\hat{\boldsymbol{e}}_{z_{optical}}$ but rather along the direction $\hat{\boldsymbol{e}}_{z_{max}}$ defined by the point of highest axial resolution in this shifted copy of $OTF_{det}(\boldsymbol{k})$ (Fig. S2A):

$$\boldsymbol{k}_{z_{max}} = \left(\frac{2\pi NA_{det}}{\lambda_{det}}\right)\hat{\boldsymbol{e}}_{y_{optical}} + 2\pi\left[\frac{2NA_{exc}}{\lambda_{exc}} + \frac{\left(n - \sqrt{n^2 - NA_{det}^2}\right)}{\lambda_{det}}\right]\hat{\boldsymbol{e}}_{z_{optical}}$$

Hence, the maximum axial resolution at this point is given by:

$$(R_{z_{optical}})_{max} = \frac{2\pi}{\boldsymbol{k}_{z_{max}} \cdot \hat{\boldsymbol{e}}_{z_{optical}}} = \left[\frac{2NA_{exc}}{\lambda_{exc}} + \frac{\left(n - \sqrt{n^2 - NA_{det}^2}\right)}{\lambda_{det}}\right]^{-1} \qquad (S10c)$$

Fourth, by Eqs. (S9), the support of $OTF_{overall}(\boldsymbol{k})$ in the $k_y k_z$ plane is approximately rectangular, because it is given by the convolution of $OTF_{exc}^{swept}(k_z)$ with $OTF_{det}(\boldsymbol{k})$ along $\hat{\boldsymbol{e}}_{z_{optical}}$. The theoretical resolution is thus particularly high in the diagonal direction:

$$\boldsymbol{k}_{yz_{diag}} = \left(\frac{4\pi NA_{det}}{\lambda_{det}}\right)\hat{\boldsymbol{e}}_{y_{optical}} + \left(\frac{4\pi NA_{exc}}{\lambda_{exc}}\right)\hat{\boldsymbol{e}}_{z_{optical}}$$

given by the point of highest lateral resolution in the axially furthest shifted copy of $OTF_{det}(\boldsymbol{k})$ (Fig. S2A), where:

$$R\left(\hat{\boldsymbol{e}}_{yz_{diag}}\right) = \frac{2\pi}{k_{yz_{diag}}} = \left[\left(\frac{2NA_{det}}{\lambda_{det}}\right)^2 + \left(\frac{2NA_{exc}}{\lambda_{exc}}\right)^2\right]^{-1/2} \qquad (S10d)$$

The theoretical resolution limits in the direction $\hat{\boldsymbol{e}}_{x_{optical}} = \hat{\boldsymbol{e}}_{z_{optical}} \times \hat{\boldsymbol{e}}_{y_{optical}}$ depends on the way in which $PSF_{exc}^{fixed}(\boldsymbol{x})$ is moved along $\hat{\boldsymbol{e}}_{x_{optical}}$ to create a light sheet. In the swept mode (supplementary note S1A), the excitation does not contribute to the resolution in the direction $\hat{\boldsymbol{e}}_{x_{optical}}$, and the results above involving $\hat{\boldsymbol{e}}_{y_{optical}}$ still hold true:

$$R\left(\hat{\boldsymbol{e}}_{x_{optical}}^{swept}\right) = \frac{\lambda_{det}}{2NA_{det}} \qquad (S10e)$$

$$R\left(\hat{\boldsymbol{e}}_{xz_{diag}}^{swept}\right) = \frac{2\pi}{k_{xz_{diag}}} = \left[\left(\frac{2NA_{det}}{\lambda_{det}}\right)^2 + \left(\frac{2NA_{exc}}{\lambda_{exc}}\right)^2\right]^{-1/2} \qquad (S10f)$$

In the confocal mode (supplementary note S1B), the convolution of $OTF_{det}^{conf}(\boldsymbol{k})$ with $OTF_{exc}(\boldsymbol{k})$ (Eq. (S4d)) extends the support along $\hat{\boldsymbol{e}}_{x_{optical}}$ to the sum of the supports of the excitation and detection individually:

$$R\left(\hat{\boldsymbol{e}}_{x_{optical}}^{conf}\right) = \left[\frac{2NA_{det}}{\lambda_{det}} + \frac{2NA_{exc}}{\lambda_{exc}}\right]^{-1} \qquad (S10g)$$

although the weakness of the OTFs near their individual supports makes the confocal OTF exceptionally weak near its lateral support. Finally, for both the incoherent and coherent structured illumination modes (Secs. S1C, S1D), the support of $OTF_{exc}^{SI}(\boldsymbol{k})$ along $\hat{\boldsymbol{e}}_{x_{optical}}$ is given by the highest harmonic in Eqs. (S5b) or (S8c), which convolved with $OTF_{det}^{conf}(\boldsymbol{k})$ gives (Fig. S2B):

$$R\left(\hat{\boldsymbol{e}}_{x_{optical}}^{SI}\right) = \left[\frac{J}{\mathrm{T}} + \frac{2NA_{det}}{\lambda_{det}}\right]^{-1} \qquad (S10h)$$

where J is the largest integer for which $J < 2NA_{exc}\mathrm{T}/\lambda_{exc}$. $OTF_{overall}^{cSI}(\boldsymbol{k})$ for the coherent mode is generally much stronger near its expanded support along $\hat{\boldsymbol{e}}_{x_{optical}}$ than either $OTF_{overall}^{conf}(\boldsymbol{k})$ or $OTF_{overall}^{iSI}(\boldsymbol{k})$, since $OTF_{exc}^{cSI}(\boldsymbol{k})$ is itself much stronger.

In LLSM, both objectives are tilted (Fig. S2C) at an angle α in the $xz_{specimen}$ plane ($\alpha = 32.45°$ for the LLSM used here) defined by the directions parallel and perpendicular to the sample substrate in order to fit within the 2π steradian space above the substrate. The resolution in the directions $\hat{\boldsymbol{e}}_{x_{specimen}}$ and $\hat{\boldsymbol{e}}_{z_{specimen}}$ are of particular interest for cultured cells. These are given by the furthest projections of the four corners of $OTF_{overall}(\boldsymbol{k})$ onto the $\hat{\boldsymbol{e}}_{x_{specimen}}$ and $\hat{\boldsymbol{e}}_{z_{specimen}}$ axes. From Eq. (S10d) and Fig. S2C:

$$(R_{x_{specimen}})_{max} = \left[\left(\frac{2NA_{det}}{\lambda_{det}}\right)^2 + \left(\frac{2NA_{exc}}{\lambda_{exc}}\right)^2\right]^{-1/2} \Big/ \cos(\psi_{yz} - \alpha) \qquad (S10i)$$

$$(R_{z_{specimen}})_{max} = \left[\left(\frac{2NA_{det}}{\lambda_{det}}\right)^2 + \left(\frac{2NA_{exc}}{\lambda_{exc}}\right)^2\right]^{-1/2} \Big/ \sin(\psi_{yz} + \alpha) \quad \text{where:} \quad (S10j)$$

$$\psi_{yz} = \mathrm{atan}\left(\frac{\lambda_{det}NA_{exc}}{\lambda_{exc}NA_{det}}\right) \qquad (S10k)$$

In the direction $\hat{\boldsymbol{e}}_{y_{specimen}} = \hat{\boldsymbol{e}}_{x_{optical}}$, the resolution is given by Eqs. (S10e) and (S10h) for the swept and SIM modes, respectively.

S3. General Experimental Considerations, Continued

There exist a number of metrics by which the performance of different light sheets can be compared, both theoretically and experimentally. These include:

A. Spatial resolution

In a microscope, the image $I(\boldsymbol{x})$ is given by the convolution of the specimen $S(\boldsymbol{x})$ with the overall PSF of the microscope:

$$I(\boldsymbol{x}) = S(\boldsymbol{x}) \otimes_{xyz} PSF_{overall}(\boldsymbol{x}) \quad \text{or, equivalently:} \qquad (S11a)$$

$$FT_{xyz}\{I(\boldsymbol{x})\} = FT_{xyz}\{S(\boldsymbol{x})\} \cdot OTF_{overall}(\boldsymbol{k}) \qquad (S11b)$$

The support, where $OTF_{overall}(\boldsymbol{k}) \rightarrow 0$, provides a hard limit in $\boldsymbol{k}$ space beyond which information about $S(\boldsymbol{x})$ cannot be recovered by traditional means. However, to minimize photobleaching and phototoxicity, live imaging requires modest photon counts in $I(\boldsymbol{x})$. Consequently, Poisson noise is the dominant noise source in experimental images:

$$FT_{xyz}\{I_{exp}(\boldsymbol{x})\} = FT_{xyz}\{S(\boldsymbol{x})\} \cdot OTF_{overall}(\boldsymbol{k}) + N(\boldsymbol{k}) \qquad (S11c)$$

where $N(\boldsymbol{k})$ represents a flat white noise spectrum. Information in $I_{exp}(\boldsymbol{x})$ becomes unrecoverable in a practical sense at specific spatial frequencies $\boldsymbol{k}'$ where:

$$N(\boldsymbol{k}') > FT_{xyz}\{S(\boldsymbol{x})\} \cdot OTF_{overall}(\boldsymbol{k}') \qquad (S11d)$$

From these equations we draw several conclusions (*14*):

i. The experimental resolution depends on the noise in the image – the theoretical resolution as defined by the support of $OTF_{overall}(\boldsymbol{k})$ can only be approached at a sufficiently high signal-to-noise ratio (SNR). Here we compare all light sheets under two experimental limits: a single image volume acquired at high SNR (~1.0×10^3 peak photon counts/pixel) to test whether the theoretical limits can be approached under optimal experimental conditions; and a time series of 100 image volumes at a more modest SNR (~2.5×10^2 peak photon counts/pixel above background) consistent with long term high speed non-invasive imaging.

ii. The experimental resolution and the fidelity of deconvolved images depend on the accuracy to which the experimental $OTF_{overall}^{exp}(\boldsymbol{k})$ is known. Because $OTF_{overall}^{exp}(\boldsymbol{k})$ is determined from $FT_{xyz}\{PSF_{overall}^{exp}(\boldsymbol{x})\}$, it is important that: a) $PSF_{overall}^{exp}(\boldsymbol{x})$ be measured from a sub-diffractive fluorescent bead at a sufficiently high SNR (>1.0×10^4 peak photon counts/pixel for the measurements herein) such that the noise in $OTF_{overall}^{exp}(\boldsymbol{k})$ contributes little to the deconvolved image $I_{deconv}(\boldsymbol{x})$ compared to the noise in $I_{exp}(\boldsymbol{x})$; and b) $PSF_{overall}^{exp}(\boldsymbol{x})$ be measured over a volume that encompasses all possible sidelobes that could contribute measurable signal, so that such signal can be accurately reassigned to its point of origin during deconvolution.

iii. The experimental resolution depends on the spatial frequency distribution $FT_{xyz}\{S(\boldsymbol{x})\}$ of the specimen. Sparse specimens dominated by puncta or lines of sub-diffractive width exhibit strong frequency spectra throughout the support and therefore more readily produce images with measurable high spatial frequency content above the noise floor for a given SNR. However,

biological specimens are often densely labeled and/or contain a broad range of feature sizes, from sub-diffractive to many microns. Such specimens exhibit spectra $FT_{xyz}\{S(\boldsymbol{x})\}$ strongly weighted towards DC, and the DC peak is further enhanced by the non-negativity of fluorescence emission. In evaluating performance across different light sheets, it is important to use densely fluorescent 3D specimens representative of this common but more challenging limit for comparison.

iv. Due to iii, the experimental resolution cannot be determined from $PSF_{overall}^{exp}(\boldsymbol{x})$ or $OTF_{overall}^{exp}(\boldsymbol{k})$, but only from where $FT_{xyz}\{I_{exp}(\boldsymbol{x})\}$ reaches its noise floor. However, due to Eq. (S11c), $FT_{xyz}\{I_{exp}(\boldsymbol{x})\}$ only contains spatial frequencies that exist in $FT_{xyz}\{S(\boldsymbol{x})\}$. Therefore, in order to have the capability of measuring potential spatial frequencies up to the theoretical support, the specimen itself must contain substantial amplitudes of all frequencies.

v. Based on iii and iv, light sheets should be compared using a standard living specimen that is dense in both real and reciprocal space. For this reason, we choose the endoplasmic reticulum (ER) of cultured LLC-PK1 pig kidney cells for such comparisons, as its thin tubules and complex sheets form a dense and intricate 3D network, particularly in the perinuclear region.

B. Deconvolution and accuracy of image reconstruction

An even more important metric is that the microscope must provide an accurate representation of the specimen to within the limits of its resolution. However, by Eq. (S11b), *every* microscope acts as a low pass filter that unevenly transmits to the raw image $I_{exp}(\boldsymbol{x})$ information about the specimen $S(\boldsymbol{x})$. Accurate deconvolution is therefore necessary in any microscope to compensate for this filtering and produce a reconstructed image $I_{deconv}(\boldsymbol{x})$ that closely matches $S(\boldsymbol{x})$. In LLSM, deconvolution is principally important, as sidelobes flanking the central excitation band of the light sheet can generate fluorescence at points well away from the center of $PSF_{overall}^{exp}(\boldsymbol{x})$. These can lead to ghost images of the sample structure in $I_{exp}(\boldsymbol{x})$ that require accurate deconvolution to assign them to their true sources. Relatedly, $OTF_{overall}^{exp}(\boldsymbol{k})$ of certain LLSs contain deep troughs that transmit sample information very weakly. Recovering this information without introducing excessive noise can give a more complete representation of $S(\boldsymbol{x})$. In addition, $PSF_{overall}(\boldsymbol{x})$ and $OTF_{overall}(\boldsymbol{k})$ degrade for light sheets of all types along their propagation direction y with increasing distance from the focal point, even within their typical range of use. The accuracy of reconstruction must therefore be verified across the entire field of view. Finally, photobleaching causes the SNR of $I_{exp}(\boldsymbol{x},t)$ to decrease over time in 4D movies of subcellular dynamics, and reconstruction parameters may have to change accordingly to avoid introducing artifacts or overamplifying noise.

In this work, we use iterative Richardson-Lucy (RL) deconvolution (*37-39*). To avoid any aliasing or interpolation errors, we measure the experimental PSF required for this purpose in the same sample scan coordinates (skewed space) as the raw image data, with the same sample step size Δx_{sp} and camera integration time per plane. Furthermore, to ensure that fluorescence generated by all sidelobes is correctly reassigned (which is essential to eliminate ghosting artifacts) we measure the PSF over a 3D FOV in skewed space that encompasses all sidelobe emission

within the excitation envelope of the light sheet (e.g., blue curves, Movie 11) and the raw image frames over a FOV in the $xy_{optical}$ plane equal to the desired FOV (equal to y_{FWHM} in the $\hat{\boldsymbol{e}}_{y_{optical}}$ direction) within the specimen plus the FOV of the PSF. Post-deconvolution we then crop the data to desired specimen FOV.

A key parameter in RL deconvolution is the number of iterations used. Too few, and the original spatial frequencies in the specimen remain under-amplified in the final image, while the sidelobe signal is not fully reassigned. Too many, and the image noise is overamplified, known continuous structures like the ER become discontinuous, and spatial frequencies in the reconstructed image begin to exceed the theoretical support. Here we determine the optimal number of iterations by Fourier Shell Correlation (FSC) (*40*). Because this optimum varies across different regions of the specimen, we calculate the optimum for multiple subregions in the $\hat{\boldsymbol{e}}_{x_{optical}}$ direction and choose as the global optimum the mean of these measurements +2.58 standard deviations, corresponding to the 99th percentile of the distribution. In all cases we find that this results in good reconstructions of the ER with minimal artifacts and a specimen FFT that mostly fills the theoretical support region yet largely remains confined to it. More details of the approach used are given in Supplementary Information.

The best measure of the fidelity of a reconstructed image is the extent to which it conforms to known priors about the specimen. For the LLC-PK1 cells we use here for light sheet comparisons, the ER network should be continuous throughout the cell, the sparse tubules of the peripheral reticular network should exhibit no ghost images and appear near diffraction-limited in width, and the dense ER in the perinuclear region should surround the interphase nucleus.

C. Light sheet propagation characteristics

A key characteristic of any light sheet is its propagation length, often defined by the distance y_{FWHM} over which its excitation intensity exceeds 50% of its peak value at the focal point. A longer light sheet produces simultaneous fluorescence emission over a larger area, thereby reducing: a) the peak intensity required to image a given volume at a given speed, and the higher phototoxicity that can come with it; b) the number of sub-volumes required to cover a given image volume, and the overhead associated with camera readout, sample translation, and stitching overlap between sub-volumes; and c) the likelihood of sample motion induced discontinuities between adjacent sub-volumes.

Herein we compare different light sheets having similar propagation distances and explore how other properties and performance metrics vary under this constraint. Specifically, we choose $y_{FWHM} \sim 50\lambda_{exc}/n$, unless otherwise noted. Given the $\alpha = 32.45°$ between the optical and specimen axes, this yields a FOV perpendicular to the specimen substrate sufficient to image even mitotic LLC-PK1 cells up to $\sim 10\mu m$ tall at λ_{exc} = 488 nm.

Even within $|y| \leq y_{HWHM}$, $PSF_{overall}(\boldsymbol{x})$ and $OTF_{overall}(\boldsymbol{k})$ can vary substantially. Therefore, for all light sheets, we also calculate these parameters at different y, and compare to experimental measurements at the focus ($y = 0$) and near the half-width at half maximum ($y = 24\lambda_{exc}/n$).

D. Axial extent of excitation

Longer light sheets can be made either by reducing the maximum numerical aperture of the excitation or by restricting the excitation to a narrower range of maximum/minimum numerical aperture in the rear pupil. The former comes at the expense of overall axial resolution and the latter at the expense of greater excitation energy in axial sidelobes flanking the central excitation peak of the light sheet. Here we report the axial excitation profile of all light sheets $PSF_{exc}^{swept}(0,z)$ at the focal point and the longitudinal cross-section $PSF_{exc}^{swept}(y,z)$ for $y = 0$ to $200\lambda_{exc}/n$, and explore the effect of sidelobe excitation, if any, on the fidelity of image reconstruction or rate of photobleaching.

S4. Gaussian Beam Light Sheet Microscopy, Continued

The pupil field that creates a cylindrically symmetric Gaussian beam at a focus is itself Gaussian:

$$E_{pupil}^{Gauss}(x_p, z_p) = E_o \exp\left[-(x_p^2 + z_p^2)/(\rho_{NA} \cdot NA_{exc}F)^2\right] \quad \text{or:} \qquad (S12a)$$

$$E_{pupil}^{Gauss}(k_x, k_z) = E_o \exp[-(k_x^2 + k_z^2)/(\rho_{NA} \cdot 2\pi NA_{exc}/\lambda_{exc})^2] \qquad (S12b)$$

$\rho_{NA} \cdot NA_{exc} = \sigma_{NA}$ is the $1/e^2$ radius of the intensity of the Gaussian beam input at the rear pupil, normalized to the pupil radius. Using Eqs. (1d) and (2a), the stationary PSF of the Gaussian beam at the focus is given by:

$$PSF_{exc}^{Gauss}(x,0,z) \propto \exp\left[-2(\pi\rho_{NA}NA_{exc})^2 \cdot \frac{x^2 + z^2}{\lambda_{exc}^2}\right] \qquad (S13a)$$

which can be written in the form:

$$PSF_{exc}^{Gauss}(x,0,z) \propto \exp\left[-2 \cdot \frac{x^2 + z^2}{w_o^2}\right] \qquad (S13b)$$

where w_o is the $1/e^2$ radius of the intensity cross section at the focus:

$$w_o = \frac{\lambda_{exc}}{\pi\rho_{NA}NA_{exc}} \qquad (S13c)$$

The corresponding stationary OTF at the focus is given by:

$$OTF_{exc}^{Gauss}(k_x,0,k_z) \propto FT\{PSF_{exc}^{Gauss}(x,0,z)\} \propto \exp\left[-\frac{1}{8}(k_x^2 + k_z^2)w_o^2\right] \qquad (S14)$$

According to Eq. (S1b), the axial swept sheet excitation OTF at the focus is:

$$OTF_{exc}^{sGauss}(k_x,k_z) = OTF_{exc}^{Gauss}(0,0,k_z) \propto \delta(k_x)\exp\left[-\frac{1}{8}(k_z w_o)^2\right] \qquad (S15a)$$

and the cross-sectional swept sheet excitation PSF is given by its inverse FT:

$$PSF_{exc}^{sGauss}(x,z) \propto \exp\left[-2\cdot\left(\frac{z}{w_o}\right)^2\right] \qquad (S15b)$$

Applying Eqs. (S3) to Eqs. (S15a,b) then gives $OTF_{overall}^{sGauss}(k_x, 0, k_z)$ and $PSF_{overall}^{sGauss}(x, 0, z)$ at the focus. The overall OTF at the focus is then given by $OTF_{overall}^{sGauss}(k_x, 0, k_z) = FT\{PSF_{overall}^{sGauss}(x, 0, z)\}$. For points $y \neq 0$ along the propagation axis, the above parameters are calculated by evaluating the integral in Eq. (1) for $E_{exc}^{Gauss}(\boldsymbol{x})$ using $E_{pupil}^{Gauss}(k_x, k_z)$ from Eq. (S12b), and applying this to Eqs. (2), (3), (S1), and (S2). This includes $PSF_{exc}^{sGauss}(y, z)$, which shows the divergence of the Gaussian sheet with increasing distance from the excitation focus.

S5. Characteristics of Ideal Lattices of Different Symmetries

A. 1D axial standing wave

The smallest plane wave set that provides the greatest resolution extension in z for a given NA_{exc} consists of a pair wavevectors confined to the $k_x = 0$ plane, produced by the pupil field (Fig. 1Aa):

$$E_{pupil}^{zSW}(k_x, k_z) = E_o\delta(k_x)\delta(k_z \pm k_o NA_{exc}) \qquad (S16a)$$

where $\delta(k \pm k') \equiv \delta(k + k') + \delta(k - k')$. This creates an axial (z axis) standing wave (SW) $PSF_{exc}^{zSW}(x, z) = PSF_{exc}^{zSW}(z)$ (Fig. 1Ab) having a DC normalized OTF of (Fig. 1Ac):

$$OTF_{exc}^{zSW}(k_x, k_z) = \delta(k_x)\delta(k_z) + \frac{\delta(k_x)\delta(k_z \pm 2k_o NA_{exc})}{2} \qquad (S16b)$$

Because $PSF_{exc}^{zSW}(z)$ is uniform along the x axis, the swept (Fig. 1Ad) and stationary OTFs are identical (Eq. S1b):

$$OTF_{exc}^{szSW}(k_z) = OTF_{exc}^{zSW}(0, k_z) = \delta(k_z) + \frac{\delta(k_z \pm 2k_o NA_{exc})}{2} \qquad (S16c)$$

However, $PSF_{exc}^{zSW}(z)$ therefore has no non-zero harmonics in k_x and cannot provide resolution extension in x by the coherent SIM mode. For the swept mode, the overall OTF is given by (Fig. 1Af):

$$OTF_{overall}^{szSW}(\boldsymbol{k}) = \frac{OTF_{det}(k_x, k_y, k_z \pm 2k_o NA_{exc})}{2} + OTF_{det} = OTF_{overall}^{cSIzSW}(\boldsymbol{k}) \qquad (S16d)$$

where $OTF(\boldsymbol{k} \pm \boldsymbol{k}') = OTF(\boldsymbol{k} + \boldsymbol{k}') + OTF(\boldsymbol{k} - \boldsymbol{k}')$. Thus, the k_z shifted copies of $OTF_{det}(\boldsymbol{k})$ that are the source of z resolution extension are 1/2 as strong as the DC copy for the axial SW.

The axial SW light sheet is identical to a coherent multi-Bessel (MB) light sheet (Main Text 6B) of period T smaller than the diffraction limit (T $< \lambda_{exc}/NA_{exc}$). In this limit, only the two polar stripes in the $m = 0$ band of $E_{pupil}^{cMB}(k_x, k_z)$ in Eq. (9b) remain. As the annulus width

approaches zero, the light sheet becomes unbound, and the polar stripes shrink to the discrete points of Eq. (S16a).

B. 2D maximally symmetric square lattice

For all lattices, the DC region of $OTF_{overall}^{lattice}(\boldsymbol{k})$ is automatically covered by the first sum in Eq. (12b), and only gets stronger relative to the regions beyond $OTF_{det}(\boldsymbol{k})$ as more plane waves are added. Thus, usually it is unnecessary and even counterproductive to craft light sheets having pupil excitation near the $k_z = 0$ equatorial line. A useful exception is light sheets based on a maximally symmetric square lattice having the pupil field,

$$E_{pupil}^{Sq}(k_x, k_z) = E_o\delta(k_x)\delta(k_z \pm k_oNA_{exc}) + E_o\delta(k_z)\delta(k_x \pm k_oNA_{exc}) \qquad (S17a)$$

for three reasons. First, in the coherent SIM mode, the two additional illumination points (green arrows, Fig. 1Ba) on the $k_z = 0$ line extend the support of $OTF_{overall}^{cSISq}(\boldsymbol{k})$ (Fig. 1Be) by the same amount in k_x as do the two points on the $k_x = 0$ line common to both the square and axial SW lattices. Second, the stationary excitation OTF:

$$OTF_{exc}^{Sq}(k_x, k_z) = \delta(k_x)\delta(k_z) + \frac{\delta(k_z \pm k_oNA_{exc})\delta(k_x \pm k_oNA_{exc})}{2} + \frac{\delta(k_x)\delta(k_z \pm 2k_oNA_{exc})}{4} + \frac{\delta(k_z)\delta(k_x \pm 2k_oNA_{exc})}{4} \qquad (S17b)$$

has four cross terms $\delta(k_z \pm k_oNA_{exc})\ \delta(k_x \pm k_oNA_{exc})/2$ that, in the SIM mode, fill in the gaps seen in $OTF_{overall}^{sSq}(\boldsymbol{k})$ of the swept mode (light blue arrows, Fig. 1Bf). Finally, as all illumination points in the pupil are extended as lines in k_z to produce an axially confined LLS, the two equatorial points can be extended the furthest while still remaining within the annulus that dictates the light sheet propagation length. This both improves the light sheet confinement and reduces the size and depth of the troughs/gaps in $OTF_{exc}^{sSq}(k_z)$ (Fig. 1Bd) and $OTF_{overall}^{sSq}(\boldsymbol{k})$. However, these advantages come at the cost of a further two-fold diminishment of the strength of the $\pm 2k_oNA_{exc}$ axially shifted copies of $OTF_{det}(\boldsymbol{k})$ relative to those in the axial SW.

A square light sheet derived from the lattice described here is identical to a coherent MB light sheet of period $\mathrm{T} = \lambda_{exc}/NA_{exc}$. This leaves only the two polar stripes in the $m = 0$ band and the two equatorial stripes of the $m = \pm 1$ bands of $E_{pupil}^{cMB}(k_x, k_z)$ in Eq. (S8a). As the annulus width approaches zero, the light sheet becomes unbound, and these four stripes shrink to the discrete points of Eq. (S17a).

C. 2D maximally symmetric hexagonal lattice

For either the axial SW or the swept mode of the maximally symmetric square lattice, as NA_{exc} is increased to increase the axial support $(R_{z_{optical}})_{max}$ of Eq. (S10c), the gap between the DC copy and the $k_z = \pm 2k_oNA_{exc}$ shifted copies of $OTF_{det}(\boldsymbol{k})$ in $OTF_{overall}(\boldsymbol{k})$ increases, until eventually $OTF_{overall}(\boldsymbol{k})$ becomes discontinuous. This occurs when the shift is larger than the maximum width of the "bowtie" region of $OTF_{det}(\boldsymbol{k})$ or, using Eq. (S10c), when:

$$NA_{exc} > \frac{\lambda_{exc}}{\lambda_{det}}\left(n - \sqrt{n^2 - NA_{det}^2}\right) \qquad (S18)$$

The consequences of gaps or even complete discontinuities in $OTF_{overall}(\boldsymbol{k})$ will be explored below. However, one solution for the axial SW or swept square lattice is to add illumination points in the rear pupil to create additional shifted copies of $OTF_{det}(\boldsymbol{k})$ at the exact centers $k_z^{OTF} = \pm k_o NA_{exc}$ of their gaps. This requires illumination points at $k_z^{pupil} = \pm k_o NA_{exc}/2$ in the pupil. For an ideal non-diffracting 2D lattice these points must also lie on the same circle of radius $k_o NA_{exc}$ upon which the polar illumination points lie. Thus, $k_x^{pupil} = \pm\frac{\sqrt{3}}{2} k_o NA_{exc}$, and:

$$E_{pupil}^{Hex}(k_x, k_z) = E_o\delta(k_x)\delta(k_z \pm k_o NA_{exc}) + E_o\delta\left(k_x \pm \frac{\sqrt{3}k_o NA_{exc}}{2}\right)\delta\left(k_z \pm \frac{k_o NA_{exc}}{2}\right) \quad (S19a)$$

This describes six illumination points equally spaced azimuthally on a ring of radius $k_\rho = k_o NA_{exc}$ in the pupil (Fig. 1Ca). These are the exact conditions that produce an ideal maximally symmetric lattice of hexagonal symmetry (Fig. 1Cb).

The six wavevectors arising from this illumination create an $OTF_{exc}^{Hex}(k_x, k_z)$ having nineteen discrete non-zero spatial frequencies in a hexagonal array (Fig. 1Cc):

$$\begin{aligned} OTF_{exc}^{Hex}(k_x, k_z) = \delta(k_x)&\left[\delta(k_z) + \frac{\delta(k_z \pm k_o NA_{exc})}{3} + \frac{\delta(k_z \pm 2k_o NA_{exc})}{6}\right] + \\ &\delta\left(k_x \pm \frac{\sqrt{3}k_o NA_{exc}}{2}\right)\left[\delta\left(k_z \pm \frac{k_o NA_{exc}}{2}\right) + \delta\left(k_z \pm \frac{3k_o NA_{exc}}{2}\right)\right]/3 + \\ &\delta\left(k_x \pm \sqrt{3}k_o NA_{exc}\right)\left[\frac{\delta(k_z)}{3} + \frac{\delta(k_z \pm k_o NA_{exc})}{6}\right] \end{aligned} \qquad (S19b)$$

that, when convolved with $OTF_{det}(\boldsymbol{k})$ according to Eq. (S9), yields a gap-free $OTF_{overall}^{cSIHex}(\boldsymbol{k})$ in the SIM mode that is reasonably uniform throughout its support (Fig. 1Ce).

In the swept mode, by Eq.(S1b) only the terms having $\delta(k_x)$ in $OTF_{exc}^{Hex}(k_x, k_z)$ remain in $OTF_{exc}^{sHex}(k_z)$ (Fig. 1Cd). This leaves five copies of $OTF_{det}(\boldsymbol{k})$ in $OTF_{overall}^{sHex}(\boldsymbol{k})$, including two (gold arrows, Fig. 1Cf) that are centered at and help fill the gaps between the DC and $k_z = \pm 2k_o NA_{exc}$ shifted copies present in the axial SW. However, these furthest shifted copies are three-fold weaker than those of the axial SW.

A hexagonal light sheet derived from the lattice described here is identical to a coherent MB light sheet of period $\mathrm{T} = (2/\sqrt{3})\lambda_{exc}/NA_{exc}$. This leaves only the two polar stripes in the $m = 0$ band and two pairs of stripes each from the $m = \pm 1$ bands of $E_{pupil}^{cMB}(k_x, k_z)$ in Eq. (S8b). As the annulus width approaches zero, the light sheet becomes unbound, and these six stripes shrink to the discrete points of Eq. (S19a).

D. 2D hexagonal-rectangular aperiodic pattern

In the case of an ideal, infinite hexagonal lattice, the $k_z = \pm k_o NA_{exc}$ shifted copies of $OTF_{det}(\boldsymbol{k})$ in $OTF_{overall}^{sHex}(\boldsymbol{k})$ do not completely fill the gaps between the DC and $k_z = \pm 2k_o NA_{exc}$

copies, but rather leave a pair of smaller gaps flanking each of the $k_z = \pm k_o NA_{exc}$ copies. As NA_{exc} increases further, so do these four gaps. Following the same procedure as above, these gaps can be filled by adding eight more illumination points on the ring of $k_\rho = k_o NA_{exc}$ in the pupil at $k_z^{pupil} = \pm k_o NA_{exc}/4$ and $k_z^{pupil} = \pm 3k_o NA_{exc}/4$ (Fig. 1Da). This produces a complex, aperiodic interference pattern at the specimen focal plane (Fig. 1Db) consisting of 91 discrete spatial frequencies in $OTF_{exc}^{HexRect}(k_x, k_z)$ (Fig. 1Dc) which, by its aperiodic nature, cannot be applied to coherent structured illumination reconstruction to extend the x resolution. However, by Eq. (11), if the pattern is swept far enough, then the resulting $OTF_{exc}^{sHexRect}(k_z)$ (Fig. 1Dd) is the incoherent sum of the swept excitation OTFs of the hexagonal lattice above and two rectangular lattices of periods $(4/\sqrt{15})\lambda_{exc}/NA_{exc}$ and $(4/\sqrt{7})\lambda_{exc}/NA_{exc}$ corresponding to the illumination points at $k_z^{pupil} = \pm k_o NA_{exc}/4$ and $k_z^{pupil} = \pm 3k_o NA_{exc}/4$, respectively. Thus, we describe this as a hexagonal-rectangular (hexrect) aperiodic pattern. The $OTF_{overall}^{sHexRect}(\boldsymbol{k})$ (Fig. 1Df)) consists of nine copies of $OTF_{det}(\boldsymbol{k})$ equally spaced in k_z which further minimizes the volume occupied by gaps. However, because fourteen wavevectors are needed to produce the pattern, the DC copy is substantially stronger than all others – 14× stronger in the case of the $= \pm 2k_o NA_{exc}$ copies that give the greatest resolution extension in z.

Because the hexrect pattern is aperiodic, it is not related to a coherent MB light sheet. However, from the trends in Fig. 1, it is clear that as more illumination points are added to the pupil, the swept overall OTF becomes increasingly continuous but increasingly also dominated by the DC portion. In particular, the hexrect pattern, with sixteen illumination points, approaches the characteristics of a single swept Bessel beam (Fig. S5). In addition, as more illumination points are added, the maxima of the resulting coherent pattern become further spaced, requiring higher peak power to image at a given speed. Thus, as NA_{exc} is increased to increase the axial support, the lattice requiring the fewest number of illumination points to achieve the desired propagation length while still enabling faithful post-deconvolution image reconstruction should be selected.

S6. Calculation of field synthesis PSF band contributions to the multi-Bessel swept PSF

By Eq. (11) the swept sheet excitation PSF of a MB LLS is given by the incoherent sum of the excitation PSFs formed by the $2M + 1$ individual bands of fixed k_x in the pupil:

$$PSF_{exc}^{scMB}(0,z) = \sum_{m=-M}^{M} FT_{k_z}^{-1}\{E_{band}(m)\} \cdot FT_{k_z}^{-1}\left\{\overline{E_{band}(m)}\right\} = \sum_{m=-M}^{M} PSF_{band}(m) \quad (S20)$$

In the field synthesis approach (*1*), each band is a uniform intensity stripe that spans and is cropped by the annular pupil. There are two types of bands. Those that symmetrically span the k_x axis are identical to the sinc light sheet case and form a single beamlet. By Eq. (6b), they therefore each contribute a term:

$$PSF_{band}(m) \propto \text{sinc}^2((k_z^+)_m z) \text{ for } k_\rho^{max} > 2\pi|m|/\text{T} > k_\rho^{min} \quad \text{where:} \quad (S21a)$$

$$(k_z^+)_m = \sqrt{(k_\rho^{max})^2 - (2\pi m/\mathrm{T})^2} \qquad (S21b)$$

Bands with $2\pi|m|/T < k_\rho^{min}$ are split by the inner circle of the mask to produce a pair of beamlets in the pupil given by:

$$E_{band}(m) = E_o\,\delta\left(k_x - \frac{2\pi m}{\mathrm{T}}\right)\left\{\mathrm{rect}\left[\frac{k_z - \left(k_z^{mid}\right)_m}{\left(k_z^{range}\right)_m}\right] + \mathrm{rect}\left[\frac{k_z + \left(k_z^{mid}\right)_m}{\left(k_z^{range}\right)_m}\right]\right\} \text{ where: } (S21c)$$

$$\left(k_z^{mid}\right)_m = [(k_z^+)_m + (k_z^-)_m]/2, \quad \left(k_z^{range}\right)_m = (k_z^+)_m - (k_z^-)_m \qquad (S21d)$$

$$(k_z^-)_m = \sqrt{(k_\rho^{min})^2 - (2\pi m/\mathrm{T})^2} \qquad (S21e)$$

Inserting Eq. (S21c) into Eq. (11) then gives:

$$PSF_{band}(m,z) \propto \cos^2\left[\left(k_z^{mid}\right)_m z\right]\mathrm{sinc}^2\left[\left(k_z^{range}\right)_m z/2\right] \quad \text{for } k_\rho^{min} < \frac{2\pi|m|}{\mathrm{T}} \quad (S22)$$

Note that Eq. (S22) is actually valid for all k_ρ^{min}, because $\left(k_z^{mid}\right)_m = 0$ for $k_\rho^{max} > \frac{2\pi|m|}{\mathrm{T}} > k_\rho^{min}$, and then $\cos^2\left[\left(k_z^{mid}\right)_m z\right] = 1$.

S7. Axially confined (AC) lattice light sheets of specific symmetries

i. AC axial SW light sheet microscopy

An AC SW light sheet is created by a single polar pair of pupil beamlets, and thus is not subject to the same tradeoffs between multiple pupil bands characteristic of other LLSs. By Eq. (S8c), $OTF_{exc}^{sSW}(k_z)$ is given by the autocorrelation of the pair. Thus, for:

$$NA_{exc} \lesssim 2\sigma_{NA} \qquad (S23)$$

the DC term from the autocorrelation bridges the gap between the cross-correlation terms from the two beamlets to produce a gap-free excitation OTF and, by the convolution in Eq. (S3b), a gap-free overall OTF. This condition is explored theoretically and experimentally for an AC SW light sheet with $NA_{exc} = 0.25$, $\sigma_{NA} = 0.13$, and $y_{FWHM} \sim 50\lambda_{exc}/n$ in Fig. S25 and Movie 14. As with the Gaussian and sinc beams of low NA_{exc}, the pupil field consists of a pair of laterally offset axial SW pupil patterns, which in turn each consist of a pair of vertically offset beamlets, in order to avoid their clipping by the inner diameter of the annulus. As a result, the pupil field consists of four beamlets (Figs. S25C,D) that together produce a rectangular stationary LLS in the specimen (Figs. S25A,B). When this is swept, it produces a LLS equivalent to an axial SW of the desired NA_{exc}, σ_{NA}, and y_{FWHM}.

As with all AC LLSs, to achieve higher axial resolution for the same light sheet length, NA_{exc} must increase and σ_{NA} must decrease, resulting in increasingly wide OTF gaps. This problem is most severe for the axial SW, since it consists of only two beamlets of the widest possible

separation in k_z for a given NA_{exc}, and is explored in Fig. S11 for $NA_{exc} = 0.30$, $\sigma_{NA} = 0.10$. Under these conditions, $PSF_{exc}^{sSW}(z)$ exhibits a broad set of strong sidebands (Fig. S11E and orange curve, Fig. S11G). The innermost pair are not fully suppressed by the axial envelope of $PSF_{det}(\boldsymbol{x})$ (blue curve, Fig. S11G), leaving a weak pair of sidebands in $PSF_{overall}^{sSW}(\boldsymbol{x})$ (red arrows, Fig. S11G,H) that create parallel ghost features in both a simulated raw image of the stripe test pattern (red arrows, Fig. S11M) and xz raw views of live LLC-PK1 cells (red arrows, Fig. S11P). In addition, the theoretical OTF exhibits deep gaps, although these are partially filled in the experimental case (light blue arrows, Fig. S11I,J). Despite these issues, after 15 and 55 iterations respectively of RL deconvolution (Movie 15, parts 1 & 3), the sideband signals are correctly assigned to their true origins in the images and removed from the deconvolved results (Figs. S11N,O,&Q), leaving a minimum resolvable line separation of 550 nm (green lines Fig. S11O) and a FT of the cell volume that fills most of its support region (inset Fig. S11Q). Thus, despite the strong excitation sidelobes, the parallel ghosts in the raw data from the sidelobes of the overall PSF, and the deep OTF gaps, the axial SW light sheet at $y_{FWHM} \sim 50\lambda_{exc}/n$ can still yield accurate image reconstructions up to $NA_{exc} \sim 0.30$.

Whereas the results of Figs. 2B and S11 show that strong excitation sidelobes and deep gaps in the overall OTF need not compromise accurate volumetric image restoration, a truly discontinuous overall OTF is a different matter. By Eq. (S18), this occurs for a SW light sheet with $\lambda_{exc}/\lambda_{det} = 0.94$, $n = 1.33$, and $NA_{det} = 1.0$ when $NA_{exc} > 0.426$. In Fig. S17, this is approximated with $NA_{exc} = 0.45$ and $\sigma_{NA} = 0.065$, where the "bowtie" regions of the three copies of $OTF_{det}(\boldsymbol{k})$ in $OTF_{overall}^{sSW}(\boldsymbol{k})$ have little to no overlap (light blue arrows, Fig. S17I and yellow-green arrows, Fig. S17J). As a result, the excitation sidelobes of $PSF_{exc}^{sSW}(z)$ extend over $z > 20\,\lambda_{exc}/n$ (orange curve, Fig. S17G), and the pair immediately flanking the central peak are suppressed only 50% in $OTF_{overall}^{sSW}(\boldsymbol{k})$ by the envelope of $OTF_{det}(\boldsymbol{k})$ (red arrows, Figs. S17G,H). This leads to even stronger parallel ghost features in both a simulated image of the stripe test pattern (red arrows, Fig. S17M) and an experimental raw image volume of live cells (red arrows, Fig. S17P) than in the $NA_{exc} = 0.30$ case of Fig. S11. However, unlike in that case, these ghosts do not fully disappear after a FSC-indicated 20 and 75 iterations of RL deconvolution, respectively (red arrows, Figs. S17N,Q, and Movie 8, parts 1 & 3), and dips between the three copies of $OTF_{det}(\boldsymbol{k})$ remain in the FFT of the image volume even after RL deconvolution (light blue arrows, inset, Fig. S17Q). The suppressed or missing spatial frequencies are evidenced as a band of unresolved lines in the raw and deconvolved simulated images of the stripe pattern (blue bands, Figs. S17M,N,O) and the artifactual punctate appearance of the deconvolved ER (Fig. S17Q). Thus, the NA_{exc} and k_z locations of the beamlets of any LLS must be chosen to ensure sufficient overlap of the shifted copies of $OTF_{det}(\boldsymbol{k})$ in the swept overall OTF in order to produce interpretable images reflective of the true sample structure.

ii. AC square LLSM

The advantages and disadvantages of an AC square LLS are primarily the inverse of those for a MB one. In the AC case, the short and equal length equatorial and polar beamlets (green and

purple arrows, Fig. S12C) result, at the focal plane, in a stronger $OTF_{overall}^{sACSq}(\boldsymbol{k})$ within the $\pm 2k_o NA_{exc}$ shifted copies of $OTF_{det}(\boldsymbol{k})$ compared to the MB case (purple arrows, Figs. S12I,J vs. Figs. S9Bi,j). Resolution in the $\hat{\boldsymbol{e}}_z$ direction as determined by the smallest observable line pair in simulated images remains similar (624 nm vs. 660 nm, green arrows, Fig. 12O vs. Fig. S9Bo, Movie 16, part 1). However, by Eq. (16) the short equatorial beamlets propagate much further than the polar ones, and hence the low spatial frequencies they contribute to $OTF_{overall}^{sACSq}(\boldsymbol{k})$ increasingly dominate the $\pm 2k_o NA_{exc}$ shifted copies with increasing y (purple arrows, Fig. S12K,L vs Fig S9Bk,l). This results in a $PSF_{overall}^{sACSq}(\boldsymbol{x})$ spatially varying in y, with gradually decreasing axial resolution within y_{HWHM}.

iii. AC hexagonal LLSM

Similar trends are seen for the AC hexagonal LLS (Fig. S13), although not as pronounced, since the difference in length of the $k_x \neq 0$ side beamlets (gold arrows, Fig. 6C) between the MB and AC cases is not as great as with the square lattice. Notably, the cropping factor ϵ (Eqs. (4d,e)) and the bounding envelope $B(z)$ (Eq. (18)) work together to produce a sharply bound version of the desired lattice at the binary SLM (Fig. S13B). This creates an effective rect(z) bounding function to the diffracted field $E_{SLM}(x,z) = E_o \exp\left(-\Phi_{SLM}(x,z)\right)$ and, since $E_{pupil}(k_x, k_z) \propto FT_{xz}(E_{SLM}(x,z))$, a sinc($k_z$) bounding function to each beamlet in the rear pupil (pink and light blue arrows, Fig. S13C). These advantageously fill the gaps (pink and light blue arrows, Figs. S13I,J) in $OTF_{overall}^{sACHex}(\boldsymbol{k})$ between the five shifted copies of $OTF_{det}(\boldsymbol{k})$ seen in the MB case (Figs. 2Bi,j). The smallest resolvable linewidth of 514 nm (green arrows, Fig. S13O) after 20 RL iterations (Movie 17, part 1) matches the estimate of $\lambda_{exc}/(NA_{exc} + \sigma_{NA})$ from Eq. (S10b). However, at y_{HWHM}, the contribution of the $\pm 2k_o NA_{exc}$ shifted copies is greatly reduced (purple arrows, Figs. S13K,L vs. Figs. 2Bk,l) due to the shorter propagation length $(y_{FWHM})_b$ of the polar beamlets, leading to a variable PSF along y and effectively reduced axial resolution.

S8. Are square lattice and sinc light sheets essentially indistinguishable?

Three papers (*8-10*) have questioned the ability of square lattices to produce light sheets having practical axial resolution superior to Gaussian light sheets of comparable length. In actuality, the light sheets they compared to square lattices were sinc and not Gaussian in nature, but the assertion required investigation. For both MB (Fig. S9B, upper right Movies 8,9) and AC (Fig. S12, middle left Movies 8,9) square LLSs, we found close agreement between the theoretical $R\left(\hat{\boldsymbol{e}}_{z_{optical}}\right)$ (651 and 659 nm, respectively) and corresponding simulation-based estimates (661 and 624 nm, respectively). Notably, these limits were well beyond the theoretical estimates of 1162 nm and 1017 nm for the Gaussian and sinc light sheets respectively as well as the simulation-based estimate of 881 nm in the sinc case (Fig. S4o). These results were corroborated by

experimental FFTs from the ER in LLC-PK1 cells in panel O of Figs. S9B, S12, and S4. There are several possible reasons for the discrepancy between our findings and those in (*8*-*10*):

• In (*10*) and (*11*), experimental "Gaussian" light sheets were generated by illuminating the pupil with a thin line along $\hat{\boldsymbol{e}}_{z_{optical}}$ of uniform intensity cropped by an adjustable slit or annulus to the desired NA_{exc}. However, these are the conditions that produce a sinc light sheet (e.g., Fig. S4), not a Gaussian one, and as described in Main Text 4, the stronger weighting of high k_z points in the pupil leads to an $OTF_{overall}(\boldsymbol{k})$ that is stronger throughout its support region than in the Gaussian case, leading to improved resolution on both the stripe test pattern and live LLC-PK1 cells and, thanks to its slower divergence with its propagation range, an $OTF_{overall}(k_z)$ is ~10x stronger near the support at $|y|\sim y_{HWHM}$.

• (*10*-*12*) compare the performance of different light sheets based on the FWHM of the central peak of $PSF_{exc}^{swept}(x,0,z)$ at the specimen focal plane. However, this does not take into account the full spatial frequency content $OTF_{exc}^{swept}(k_z)$ encoded by the overall shape of the central peak and its sidelobes, nor its interplay with $OTF_{det}(\boldsymbol{k})$ that determines $OTF_{overall}^{swept}(\boldsymbol{k}) = OTF_{exc}^{swept}(k_z)\otimes_{k_z}OTF_{det}(\boldsymbol{k})$. Theoretically, it is this latter 3D function and the 2D support surface where it falls to zero that most accurately and completely define resolution (supplementary note S3A). Experimentally, it is the 3D FFT of the specimen and its self-consistent cross-correlation that defines the practical resolution under the specific conditions of the experiment. For the nine light sheets studied here, the experimental $OTF_{overall}^{swept}(\boldsymbol{k})$ closely matched theory, and the FFT of the ER in living LLC-PK1 cells closely filled the region bound by the theoretical support (Fig. 5A).

• For example, in (*10*) a MB square LLS of $NA_{annulus} = 0.55/0.44$ identical to that of one of the LLSs in (*2*) and a length of $y_{FWHM} = 51.8\ \lambda_{exc}/n$ (19 μm at λ_{exc} = 488 nm and $n = 1.33$) had a measured FWHM of 900 nm (Table 2 of (*10*)), vs a theoretical one of 980 nm in Fig. S18Ad for a light sheet we generated with the same conditions. The stated y_{FWHM} implies $NA_{exc} \approx 0.495$. At this NA_{exc}, the equatorial beamlets alone (green arrows, Fig. S18Aa) behave equivalently to a sinc light sheet of $NA_{exc} = 0.24$, which has a theoretical FWHM of 1100 nm (Fig. S4G) and the ability to resolve the 880 nm line pair in the simulated stripe pattern (green arrows, Fig. S4O). This is recapitulated as expected for the square LLS in Fig. S18Ai and taken alone would suggest that the MB square and sinc beams of comparable NA_{exc} offer comparable resolution. However, the polar beamlets (purple arrows, arrows, Fig. S18Aa) also contribute, creating $\pm 2k_o NA_{exc}$ shifted copies (purple arrows, Fig.S18Ae) of $OTF_{det}(\boldsymbol{k})$ in $OTF_{overall}^{swept}(\boldsymbol{k})$. Due to the far greater length of the equatorial beamlets, these are much weaker than the DC copy (green arrow, Fig. S18Ae), but the central peaks of these shifted copies are still strong enough (purple arrows, Fig. S18Af) that the light sheet can resolve line spacings in the 404-514 nm range after 10 iterations of RL deconvolution (light green arrows, Fig. S18Ai), slightly beyond the theoretical limit $R\left(\hat{\boldsymbol{e}}_{z_{optical}}\right) = 444$ nm with $NA_{exc}^{max} = 0.55$. Thus, despite their comparable FWHM, this square

LLS from (*11*) has a resolution limit along $\hat{\boldsymbol{e}}_{z_{optical}}$ ~2.5× and ~2.2× greater than that of Gaussian and sinc beams of similar length, respectively. On the other hand, the large separation in k_z between the ends of the equatorial bands and the ends of the polar bands in the pupil (Fig. S18Aa) lead to a pair of wide and deep troughs in $OTF_{overall}^{swept}(\boldsymbol{k})$ (light blue arrows, Figs. S18Ae,Af) that leave unresolved the line pairs of separation 550 to 844 nm at the SNR = 20 used in the simulation (red band, Fig. S18Ai). In addition, the $\pm 2k_o NA_{exc}$ shifted copies of $OTF_{det}(\boldsymbol{k})$ are so weak that most of the spatial frequencies they cover within the support boundary, such as along the "bowtie" line of $k_x = 2\pi NA_{det}/\lambda_{det}$ (green curve and orange arrows, Fig. S18Af) are probably unrecoverable at modest SNR. However, to produce accurate reconstructions of sample structure, a microscope should be able to recover all spatial frequencies within its support boundary. Thus, this particular LLS is far from optimal.

• Notably, although the LLS from (*10*) has the same $NA_{annulus}$ as the one from (*2*) to which it was compared, it differs in two critical aspects that make the comparison unfavorable. First, in (*11*) NA_{exc} was set to just above $NA_{annulus}^{min}$ so that the equatorial beamlets (green arrows, Fig. S18Ba) had, according to Eq. (15), the same propagation length as the polar ones (Fig. S8C). Second, the cropping factor ϵ (Eqs. (4)) was chosen to adjust the z extent of sidelobe excitation in the LLS cross-section (Fig. S18Bb vs. Fig. S18Ab) and, equivalently, the relative intensity of the equatorial and polar beamlets in the pupil (green and purple arrows, Fig. S18Ba vs. Fig. S18Aa).

These optimizations resulted in a LLS far more suitable for imaging than the corresponding one in (*10*). The longer equatorial beamlets behaved equivalently to a sinc light sheet of $NA_{exc} =$ 0.32, extending the resolvability of the wider line pairs in the test pattern simulation down to 697 nm (green arrows, Fig. S18Bi), and reducing the size of the OTF troughs (light blue arrows, Figs. S18Be,f) so that the range of unresolved line pairs along $\hat{\boldsymbol{e}}_{z_{optical}}$ was reduced to only 621-664 nm at SNR = 20 (red band, Fig. S18Bi). The reduced intensity of the equatorial beamlets relative to the polar ones yielded a narrower light sheet FWHM of 590 nm (Fig. S18Bd) and $\pm 2k_o NA_{exc}$ shifted copies of $OTF_{det}(\boldsymbol{k})$ more than 3× stronger than the comparative light sheet in (1) (purple and orange arrows, Fig. S18Bf vs. Fig. S18Af). Although the k_z support and hence the smallest resolvable line pair for the two light sheets were identical (404 nm), this increased OTF strength resulted in deeper modulation depth for all line pairs. Furthermore, the OTF strength along the "bowtie" line (green lines, Fig. S18Be,f) was similar to that of the MB hexagonal LLS of Fig. 2B, which proved sufficient to recover spatial sample frequencies of the ER in live LLC-PK1 cells throughout the support region (upper right inset, Fig. 2Bo). Thus, optimization of lattice light sheets requires a thorough understanding of all input parameters, and valid comparisons require all such parameters to be identical.

• In (*11*), the contribution of the polar beamlets to square and hexagonal lattices was deemed insignificant based on PSF linecuts (supplementary note of (*11*)). This left only the paired equatorial (square) or flanking (hexagonal) beamlets, of which they generated a single copy using a uniformly illuminated stripe. Thus, the "square" and "hexagonal" lattice light sheets used in their comparisons were actually sinc and cosine-sinc light sheets having NA_{exc}^{max} values

substantially smaller than the value $NA_{annulus}^{max}$ they would have if the polar beamlets were included, and hence substantially poorer resolution $R\left(\hat{\boldsymbol{e}}_{z_{optical}}\right)$. As shown in Fig. S18, given the proper optimization of all light sheet parameters, the polar beamlets can have a profound effect on LLS performance.

S9. Limits of accurate image reconstruction for PSFs with strong sidebands

The problem of creating accurate representations of sample structure from images acquired by a microscope having an overall PSF with strong sidebands and, equivalently, deep overall OTF troughs was investigated previously (*28-30*) in comparisons of 4Pi (*31*), standing wave (SWM, (*32*)) and image interference and incoherent interference illumination (I5M, (*33*)) microscopy. The findings include:

• For accurate linear deconvolution, the primary sidelobes in the overall PSF must be no stronger than 50% of the central peak (*29*). This condition is met by all the light sheets considered here, except for the axial SW light sheet of Fig. S17, where $NA_{exc} = 0.45$ was explicitly chosen to produce a discontinuous overall OTF according to Eq. (S18) and thereby create a condition where RL deconvolution would not be able to generate an accurate reconstruction.

• The preferred embodiment in (*29*) consisted of a two-photon 4Pi type A microscope (coherent excitation, incoherent detection) with primary sidelobes of the overall PSF at 18% the strength of the central peak. With this arrangement, a raw image of microtubules in a fixed fibroblast cell exhibited clear ghost images from these sidelobes, but the corresponding linear deconvolved image produced an accurate representation of the microtubules with no ghosts (Fig. 6 of (*29*)). In comparison, here all light sheets except for that in Fig. S17 exhibited shoulders to the central peak, rather than clearly separate sidebands, comparable to or often much smaller than this, leading to accurate post-deconvolution reconstructions of the ER in live LLC-PK1 cells.

• The single-photon 4Pi type C embodiment (coherent excitation and detection) was able to produce a nearly artifact-free images of simulated test structures after RL deconvolution (Fig. 2 of (*30*) and Fig. 6C of (*28*)), despite having overall OTF troughs ~5% of the DC peak (Fig. 2 of (*29*), Fig. 2 of (*28*)) and a pair of sidelobes 40-60% as strong as the central peak (Fig. 1 of (*30*) and Fig. 4 of (*28*)) that create a pair of bright ghosts in the simulated raw image (Fig. 2 of (*30*)). In Fig. 2 of (*30*), this required 1871 RL iterations.

• The stronger the sidelobes, the more RL iterations needed to achieve an optimal deconvolved result (*30*). Similar trends are seen for the light sheets presented here.

Although the results of (*28-30*) and the measurements here are mutually consistent, they disagree with the arguments presented in (*10-12*). We consider these differences as follows:

• Both (*10*) and (*11*) cite the 50% rule of (*29*) for the maximum sidelobe height beyond which accurate image reconstruction cannot be performed as a reason to dismiss the usefulness of hexagonal lattices. However, they both refer to the sidelobes of the *excitation* PSF (e.g., Fig. 3C of (*11*)), whereas (*29*) refers to the *overall* PSF, in which the excitation sidelobes are suppressed by the envelope of $PSF_{det}(\boldsymbol{x})$. Indeed, the primary *excitation* sidelobes of the two-photon 4Pi type A arrangement that produced accurate reconstructions in (*29, 30*) were stronger than this 50% threshold, and those of the axial SW and hexagonal LLSs of Figs. 2, 4, 6 of (*11*), Fig. S11, and Fig. S25 were 76%, 93%, 100%, 55%, and 85%, respectively, of the central peak intensity. However, in all these cases, the shoulders of the corresponding *overall* PSF were $< 25\%$ of the central peak, and each one was able to achieve reconstructions largely free of ghost artifacts (Movies 5, 7, 10, and 6 of (*11*), panels Q of Figs. S11 and S25, Movies 14 and 15).

• Similarly, (*11*) and (*12*) state that hexagonal or "periodic" light sheets exhibit gaps in the their OTFs that are prone to artifacts. While OTF troughs certainly exist for nearly all the LLSs considered here, all produce accurate reconstructions after an FSC-determined optimum number of RL iterations, as seen in panels Q and the comparative $xz_{specimen}$ orthoslices in Fig. 5B. Furthermore, despite the OTF troughs, all light sheets recovered the spatial frequencies at the locations of these troughs as evidenced by the comparative FFTs of their reconstructed image volumes in Fig. 5A, even at the modest SNR $\approx$ 20 (~500 counts/pixel, including ~100 dark counts/pixel) used in these experiments.

• Both (*10*) and (*12*) argue that sidelobes of the excitation PSF introduce "blur" and "background noise" that reduce "optical sectioning" and "image contrast". However, this is relevant only if one were to rely only on raw images. In any raw image, the true sample structure is convolved with the overall PSF of the microscope, so that fluorescence emission from 3D regions outside the specimen point conjugate to any raw image voxel, including those associated with the excitation sidelobes, is incorrectly assigned to that voxel. The purpose of deconvolution is then to reassign this misassigned signal to its correct sources in the deconvolved image. Thus, to the extent that this assignment can be performed accurately, the fluorescence emission from the sidelobes represents useful signal, not obscuring haze, blur, or background noise. The results for 4pi microscopy in (*28-30*) and all the light sheets in this work (except the specifically designed counterexample of Fig. S17) demonstrate that such accurate reassignment is possible, even with strong primary excitation sidelobes. In these cases, the optical sectioning post-deconvolution is defined by the support of the overall OTF in the $\hat{\boldsymbol{e}}_{z_{optical}}$ direction which, for most lattice light sheets, is well beyond that of confocal microscopy, and the image contrast is very high.

• Of (*10-12*), only (*11*) sought to demonstrate experimentally the claim that OTF troughs and strong primary excitation sidebands lead to image artifacts, even post-deconvolution. There, a light sheet designed to mimic a swept hexagonal LLS was generated by illuminating a pupil annulus of $NA_{annulus} = 0.536/0.450$ with a single uniform stripe parallel to the k_z axis at $NA =$ 0.423 (Hex79, row 3, Fig. 3 of (*11*)). The polar beamlets of the hexagonal lattice were not

included, as they were deemed inconsequential. The light sheet therefore actually mimics a swept rectangular cosine-sinc LLS of $NA_{exc}^{max} = 0.324$ (Fig. S26) with resolution $R\left(\hat{\boldsymbol{e}}_{z_{optical}}\right) = 753$ nm and a propagation length of $y_{FWHM} = 35.5\ \lambda_{exc}/n$. In 3D images of collagen and clathrin-coated pits in ARPE cells (Figs. 4 and 5 of (*11*)), clear ghost image artifacts were seen after 10 iterations of RL deconvolution, so chosen "to avoid clipping of dim features and over-deconvolution". However, by our FSC metric, we found that the hexagonal LLSs in Figs. 2B, 3A, and S13 optimally required 55, 65, and 105 iterations, respectively, at which point each showed negligible ghost artifacts. Indeed, all these light sheets still exhibited clear blur and ghost images after only 10 iterations (e.g., Fig. S27, for the case of the MB hexagonal LLS in Fig. 2B) but no such anomalies remained after the FSC-proscribed number of iterations. Yet the LLS of Fig. 2B has higher resolution $R\left(\hat{\boldsymbol{e}}_{z_{optical}}\right) = 519$ nm and a longer propagation length of $y_{FWHM} = 48.0\ \lambda_{exc}/n$ and therefore might be expected to be more susceptible to sidelobe-generating artifacts, not less. Thus, assuming correct alignment of the light sheet to the detection focal plane, it seems likely that the ghost artifacts for the "hexagonal" LLS in (*11*) are due to insufficient deconvolution.

S10. Correction of system aberrations in the excitation light path

Optical aberrations affect both the excitation and detection pathways of a light sheet microscope and can be classed into those intrinsic to the microscope system and those specific to the specimen (*7*). We mapped the system excitation aberration by using pupil segmentation (*41*). In this process, a sinusoidal SLM pattern is used to create two points (segments) of illumination in the rear pupil of EO. These in turn form a reference beam and a measurement beam in the specimen that produce a standing wave interference pattern whose phase can be measured with a fluorescent bead and camera. The phase of the measurement beam is ramped relative to the reference beam until maximum intensity is generated from the fluorescent bead. This process is then repeated across 488 measurement points in the pupil from NA 0.22 to 0.58 to generate a complete phase correction map. Aberration corrected light sheet patterns are then produced at the SLM by transforming the ideal light sheet pattern to the pupil conjugate plane, adding the measured phase correction, and transforming the result back to the SLM.

S11. Correction of system aberrations in the detection light path

The detection system aberration was obtained using the measured 3D detection PSF from a widefield-illuminated sub-diffractive fluorescent bead. Phase retrieval (*42, 43*) calculated on the PSF revealed the amount of detection wavefront error, which was then compensated using DM. This process was iterated until the bead's PSF matched the expected theoretical Richardson and Wolf PSF (*36*).

S12. Photobleaching measurements

To assess photobleaching, we imaged human induced pluripotent stem cells (hiPSCs) gene-edited for mono-allelic expression of mEGFP-Tubulin Alpha 1B as described in the main text. To calculate the bleaching rates from the timeseries measurements: (i) three pixels around the edges of the image volumes were masked to account for any sample drift during the timeseries measurements. (ii) Fluorescent beads were computationally removed by identifying them in the final volume and applying a spherical mask to remove them at all time points. (iii) The integrated intensities from the unmasked regions at each time point were normalized by the integrated intensity of the first time point of the timeseries; (iv) the average normalized integrated intensity across six different fields of view were fitted with a single exponential function to generate the bleaching rate curves. The 95% confidence interval was generated from ± 1.96 standard deviations of the normalized integrated intensity across the six different fields of view.

S13. Deconvolution of experimental data

The live 3D cell data was processed using RL deconvolution with experimentally measured PSFs as described in S3B above. To suppress edge artifacts amplified during deconvolution near the image boundaries, we applied a 1D smoothed window function to the raw data to dampen the signals near the $y_{optical}$ image borders. The weights for this function were computed by averaging the Gaussian smoothed $xy_{optical}$ image frames ($\sigma = 50$) along $x_{optical}$ and specimen scan axis (where the lowest normalized window value near the boundary was ~0.1). In most cases, the normalized weights have a bell-shaped profile along $y_{optical}$, which significantly suppressed artifacts near the image border while having no observable effect over the portion of the image within y_{FWHM}.

To determine the optimal number of RL iterations, we used Fourier Shell Correlation (FSC). Due to the variability of features across the entire volume, the volume was subdivided into multiple sub-volumes (~22 x 22 x 22 μm^3) offset by 50 to 100 voxels along $x_{optical}$, and the limit of correlated resolution reported by FSC was plotted as a function of the number of iterations for each subvolume. The minimum of this curve represents the optimal number of iterations for that subvolume. The number of RL iterations used for the entire volume was then defined by the mean of the optimal numbers from all subvolumes +2.58 standard deviations.

Typically, the FSC for each sub-volume was calculated with a radius interval of 540 nm (5 pixels) and at an angle interval of $\frac{\pi}{12}$. We used one-bit thresholding to determine the cutoff frequencies that define the relative resolution (*44*).

S14. LLS-SIM reconstruction

We used a harmonic balanced hexagonal lattice light sheet with $NA_{exc} = 0.46,\ \sigma_{NA} = 0.10$ to demonstrate the LLS-SIM mode, collecting five phase-stepped images for SIM reconstruction as described previously (*2*). We used GPU-accelerated SIM reconstruction software (https://github.com/scopetools/cudasirecon) (*13*) with an OTF calculated from an experimentally measured PSF to reconstruct data collected in the LLS-SIM mode. We adapted this code to use cosine apodization during reconstruction. To minimize reconstruction artifacts, we split the data (*45*) into 64 pixel chunks (with 16 pixels overlap at each border) along $x_{optical}$.

S15. Stitching of tiled volumes

For short lattices (y_{FWHM}~5 µm), we acquired four tiles to cover a similar field of view as the longer light-sheets (y_{FWHM}~20 µm). These tiles were stitched into a single volume using cross-correlation to estimate and adjust the optimal tile positions, and blended to merge the overlap regions (*46*). We used feather blending with p-norm (p = 10) weights (sum normalized to 1) based on distances to the nearest volume edge.

S16. Visualization and software

The figures and 2D movies were generated using MATLAB 2022a (MathWorks®). The timeseries datasets of LLC-PK1 were rendered in 3D using Imaris 9.9 (Oxford Instruments). The theoretical and experimental OTF figure panels were generated with gamma = 0.5, and the FFT figure panels corresponding to the LLC-PK1 ER data were generated with gamma = 0.3. The stitching, FSC, combined deskew, rotation, and GPU-accelerated 3D deconvolution software packages were all implemented in MATLAB 2019a-2022a, and are available as part of the LLSM3DTools package on GitHub (https://github.com/abcucberkeley/LLSM3DTools/tree/dev).

Supporting Figures

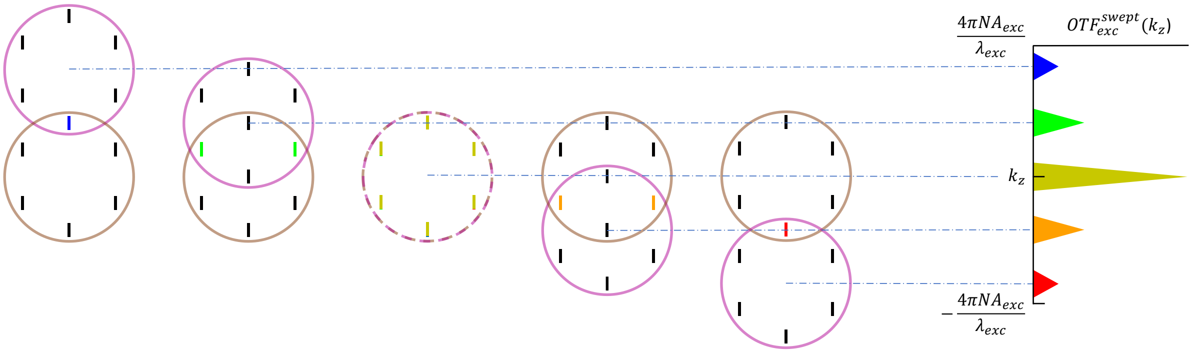

Fig. S1. Pictorial representation of the 1D autocorrelation in k_z of the pupil electric field which gives rise to the swept excitation transfer function $OTF_{exc}^{swept}(y, k_z)$. The integration that defines the autocorrelation is visualized as the translation in k_z of one copy $E_{pupil}(k_{x1}, k_{z1} - k_z)$ (bands in magenta circle) of the pupil field over a stationary identical one ($E_{pupil}(k_{x2}, k_{z2})$, bands in brown circle). For a light sheet swept in x, the integrand of the auto-correlation is non-zero only for bands (shown here in color) for which $k_{x1} - k_{x2} = 0$, creating a $OTF_{exc}^{swept}(y, k_z)$ (shown at right) with no k_x modulation, given by the incoherent sum of the independent 1D autocorrelations from each band of unique k_x.

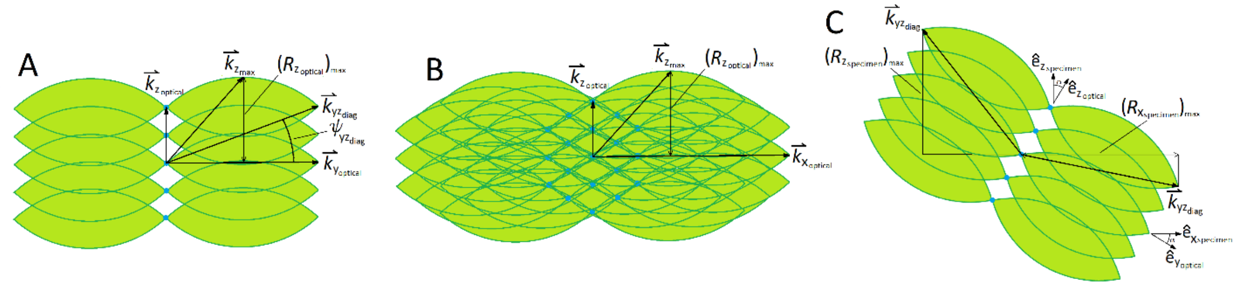

Fig. S2. Lattice light sheet overall optical transfer functions and directions of physical interest for defining resolution.

(A) Overall OTF of a swept hexagonal LLS in the $k_{yz_{optical}}$ plane in which the axes of both the excitation and detection objective lie, showing:

i) $\boldsymbol{k}_{\boldsymbol{z_{optical}}}$, the point of highest resolution $R\left(\hat{\boldsymbol{e}}_{\boldsymbol{z_{optical}}}\right)$ on the $\hat{\boldsymbol{e}}_{z_{optical}}$ axis

ii) $\left(R_{z_{optical}}\right)_{max}$, the highest resolution in the $\hat{\boldsymbol{e}}_{\boldsymbol{z_{optical}}}$ direction anywhere on the support boundary

iii) $\boldsymbol{k}_{\boldsymbol{yz_{diag}}}$, the point of highest *total* resolution $R\left(\hat{\boldsymbol{e}}_{\boldsymbol{yz_{diag}}}\right)$ anywhere on the support boundary in the $k_{yz_{optical}}$ plane

iv) $\boldsymbol{k}_{\boldsymbol{y_{optical}}}$, the point of highest resolution $R\left(\hat{\boldsymbol{e}}_{\boldsymbol{y_{optical}}}\right)$ on the $\hat{\boldsymbol{e}}_{y_{optical}}$ axis

(B) Lattice SIM overall OTF of a stepped hexagonal LLS in the $k_{xz_{optical}}$ plane defined by the direction $\hat{\boldsymbol{e}}_{\boldsymbol{x_{optical}}}$ of pattern phase stepping and the axis of the detection objective, showing $\boldsymbol{k}_{\boldsymbol{x_{optical}}}$, the point of highest resolution $R\left(\hat{\boldsymbol{e}}_{\boldsymbol{x_{optical}}}\right)$ on the $\hat{\boldsymbol{e}}_{y_{optical}}$ axis

(C) Overall OTF of a swept hexagonal LLS in the $k_{xz_{specimen}}$ plane defined by the scanning direction $\hat{\boldsymbol{e}}_{x_{specimen}}$ in the plane of the specimen substrate and the direction $\hat{\boldsymbol{e}}_{z_{specimen}}$ perpendicular to the substrate, showing:

i) $\left(R_{z_{specimen}}\right)_{max}$, the highest resolution in the $\hat{\boldsymbol{e}}_{\boldsymbol{z_{specimen}}}$ axial direction familiar to users of conventional upright and inverted microscopes

ii) $\left(R_{x_{specimen}}\right)_{max}$, the highest resolution in the $\hat{\boldsymbol{e}}_{\boldsymbol{x_{specimen}}}$ lateral direction familiar to users of conventional upright and inverted microscopes

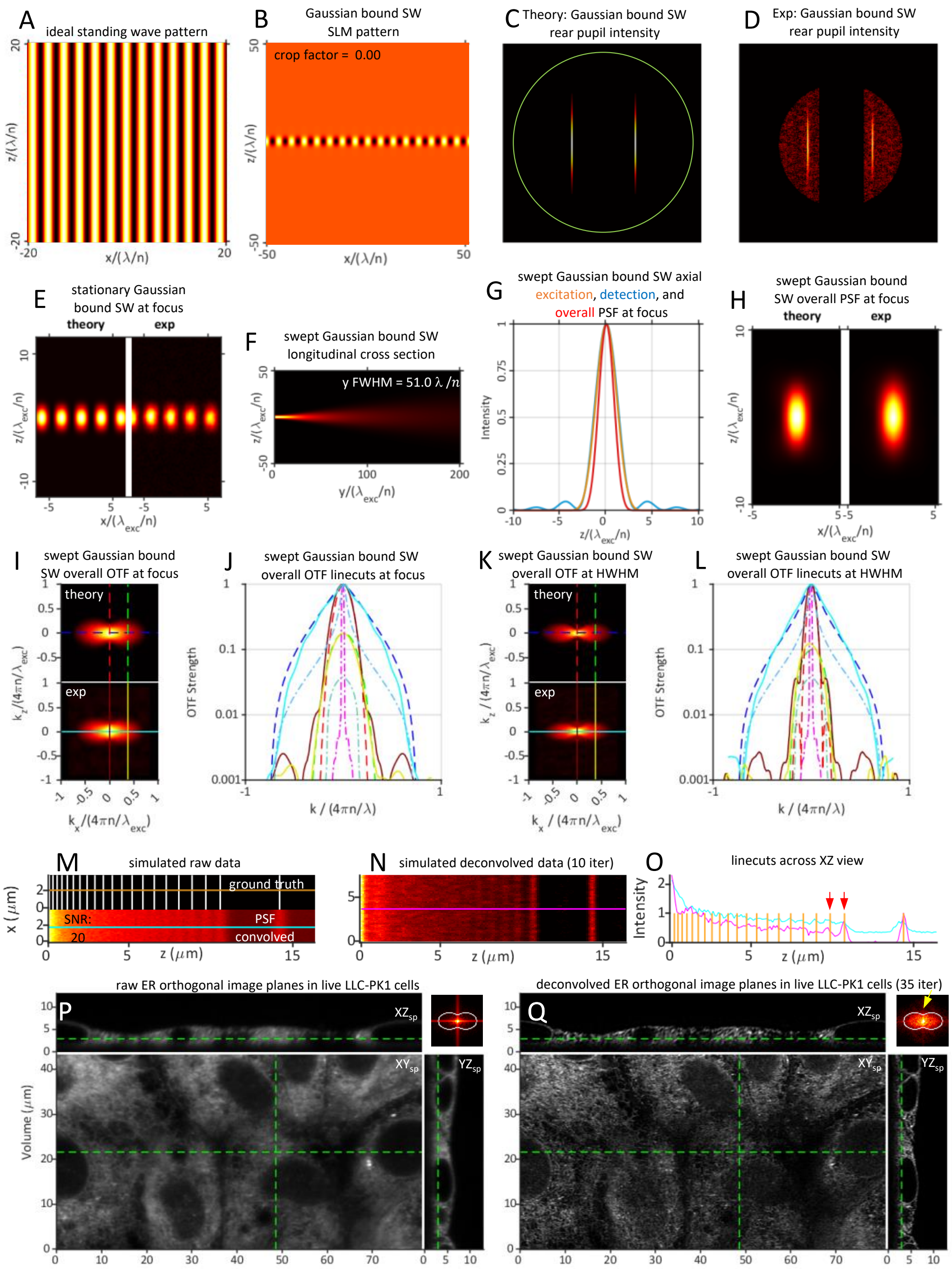

Fig. S3. Theoretical and experimentally measured characteristics of a swept Gaussian light sheet of propagation length $\boldsymbol{y_{FWHM} = 51.0\ \lambda_{exc}/n}$. The light sheet is created by a swept lateral standing wave of $NA_{exc} = 0.21$ having a Gaussian bounding envelope in k_z of $\sigma_{NA} = 0.21$, filtered by a pupil conjugate annulus of $NA_{annulus} = 0.40/0.20$. The panels above and in Figs. 2B, 3, S4, S9B, S10-S13, S17, S22, S23, and S25 show:

(A) Intensity $I_{exc}^{ideal\ fixed}(x, z)$ of the ideal excitation pattern at the focal plane upon which the light sheet is based;

(B) Grayscale phase pattern $\Phi_{SLM}(x, z)$ applied to the SLM to create the desired axially bound electric field pattern which is swept or stepped (SIM mode) to create the light sheet;

(C, D) Theoretical and experimental intensity pattern at the rear pupil of the excitation objective;
(E) Axially bound intensity pattern $I_{exc}^{fixed}(x,z)$ at the objective focal plane created by the pupil illumination pattern in **(C, D)**;
(F) Longitudinal linecut $I_{exc}^{swept}(y,z)$ through the light sheet created by sweeping the intensity pattern in **(E)** in the x direction;
(G) Theoretical axial linecuts at the focal plane through: the swept light sheet in **(F)** (orange); the point spread function $PSF_{det}(\boldsymbol{x})$ of the objective orthogonal to the light sheet used to image the emitted fluorescence (blue); and the overall $PSF_{overall}^{swept}(\boldsymbol{x}) = PSF_{det}(\boldsymbol{x}) \cdot PSF_{exc}^{swept}(\boldsymbol{x})$ that dictates the imaging properties of the microscope when the light sheet microscope is swept (red);
(H) Theoretical and experimental orthoslices through $PSF_{overall}^{swept}(\boldsymbol{x})$ at the excitation focal plane;
(I) Theoretical and experimental orthoslices through the overall optical transfer function $OTF_{overall}^{swept}(k_x, k_z)$ at the excitation focal plane;
(J) Linecuts through the theoretical (dashed lines) and experimental (solid lines) $OTF_{overall}^{swept}(k_x, k_z)$ at the locations shown in **(I)**. Also included for reference (dot-dash lines) are linecuts through $OTF_{det}(\boldsymbol{k})$ at the same locations, i.e., the k_x and k_z axes, as well as the line $k_x = 2\pi NA_{det}/\lambda_{det}$ where the widefield microscope has its highest resolution in z;
(K) Same as **(I)**, except at the $y = y_{HWHM}$ edge of the propagation region that defines the extent of the usable field of view in the y direction;
(L) Same as **(J)**, except at $y = y_{HWHM}$;
(M) Test pattern consisting of lines of variable spacing (top) and resulting raw light sheet image of the pattern (bottom);
(N) Image of the same test pattern after RL deconvolution;
(O) Linecuts through the test pattern (orange) and raw (aqua) and deconvolved (magenta) images in **(M)** and **(N)**, respectively;
(P) xy_{sp}, xz_{sp}, and yz_{sp} orthoslices in specimen coordinates at the locations denoted by dashed green lines through a raw 3D image volume of ER-labeled LLC-PK1 cells. Inset at upper right shows a $(k_x k_z)_{sp}$ orthoslice through the Fourier transform of the image volume;
(Q) Same as **(P)**, except using the deconvolved 3D image volume.

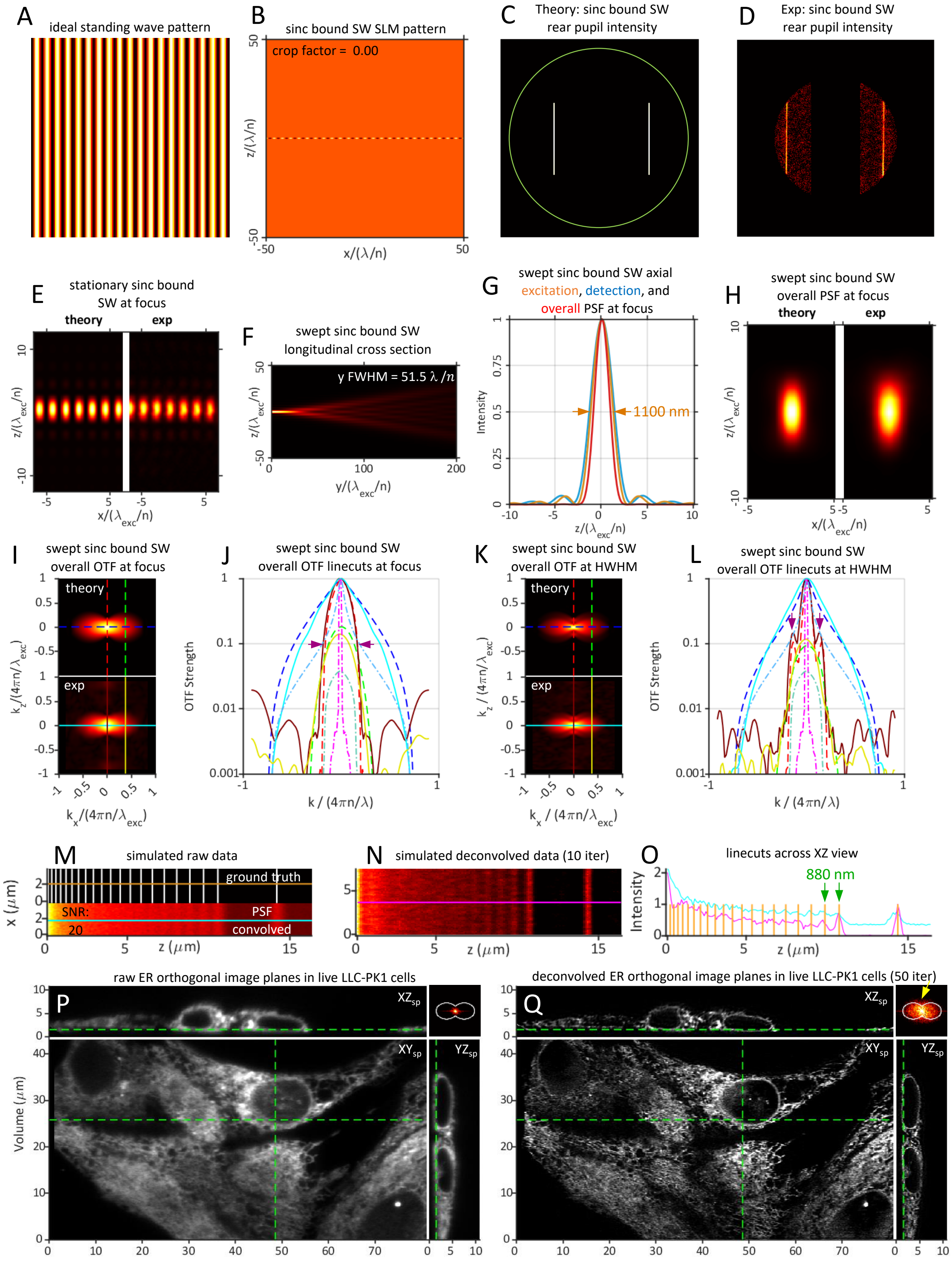

Fig. S4. **Theoretical and experimentally measured characteristics of a swept sinc light sheet of propagation length $y_{FWHM} = 51.5\ \lambda_{exc}/n$.** The light sheet is created by a pair of uniformly illuminated equatorial pupil bands of $NA_{exc} = 0.32$ filtered by an annulus of $NA_{annulus} = 0.40/0.20$ to limit the maximum NA in k_z to $NA_{sinc} = 0.24$ for the resulting swept lateral standing wave in the specimen. Panel descriptions are the same as in Fig. S3.

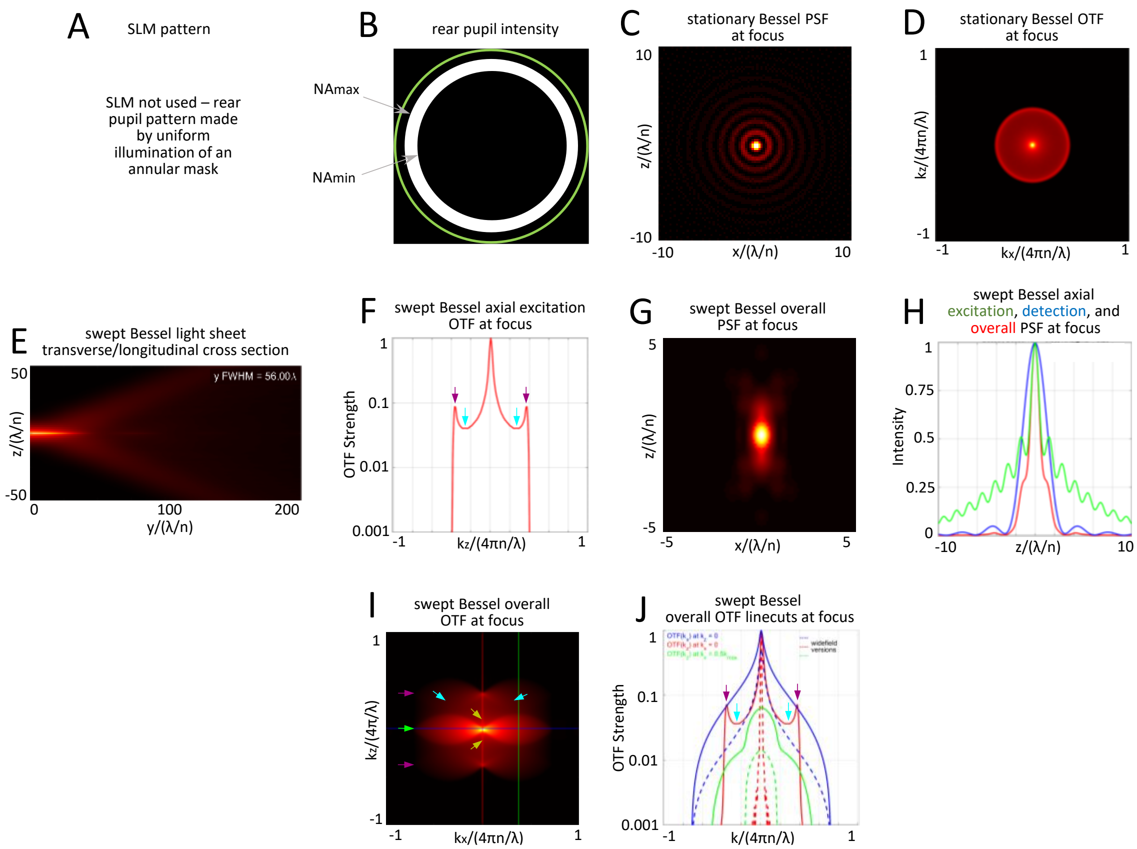

Fig. S5. Theoretical characteristics of a light sheet created by laterally sweeping a Bessel-like beam. $NA_{annulus} = 0.53/0.47$, and $y_{FWHM} = 56.0\ \lambda_{exc}/n$. The panels show:

(A) Not used here, but see Fig. S6;

(B) Pupil illumination pattern, showing maximum diameter of the pupil (green circle);

(C) Cross-sectional intensity $I_{exc}^{fixed}(x,z)$ of the fixed beam at the excitation focal plane;

(D) Optical transfer function $OTF_{exc}^{fixed}(k_x,k_z)$ of the fixed beam at the excitation focal plane;

(E) Longitudinal linecut $I_{exc}^{swept}(y,z)$ through the light sheet created by sweeping the intensity pattern in **(C)** in the x direction;

(F) Linecut through $OTF_{exc}^{fixed}(k_x,k_z)$ from **(D)**;

(G) Overall point spread function $PSF_{overall}^{swept}(\boldsymbol{x})$ of the swept beam light sheet at the excitation focal plane;

(H) Axial linecuts at the focal plane through: the swept light sheet in **(E)** (green); the point spread function $PSF_{det}(\boldsymbol{x})$ of the objective orthogonal to the light sheet used to image the emitted fluorescence (blue); and the overall $PSF_{overall}^{swept}(\boldsymbol{x})$ (red);

(I) Orthoslice through the overall swept transfer function $OTF_{overall}^{swept}(k_x,k_z)$ at the excitation focal plane;

(J) Linecuts (solid lines) through $OTF_{overall}^{swept}(k_x,k_z)$ at the locations shown in **(I)**. Also included for reference (dot-dash lines) are linecuts through $OTF_{det}(\boldsymbol{k})$ at the same locations, i.e., the k_x and k_z axes, as well as the line $k_x = 2\pi NA_{det}/\lambda_{det}$ where the widefield microscope has its highest resolution in z.

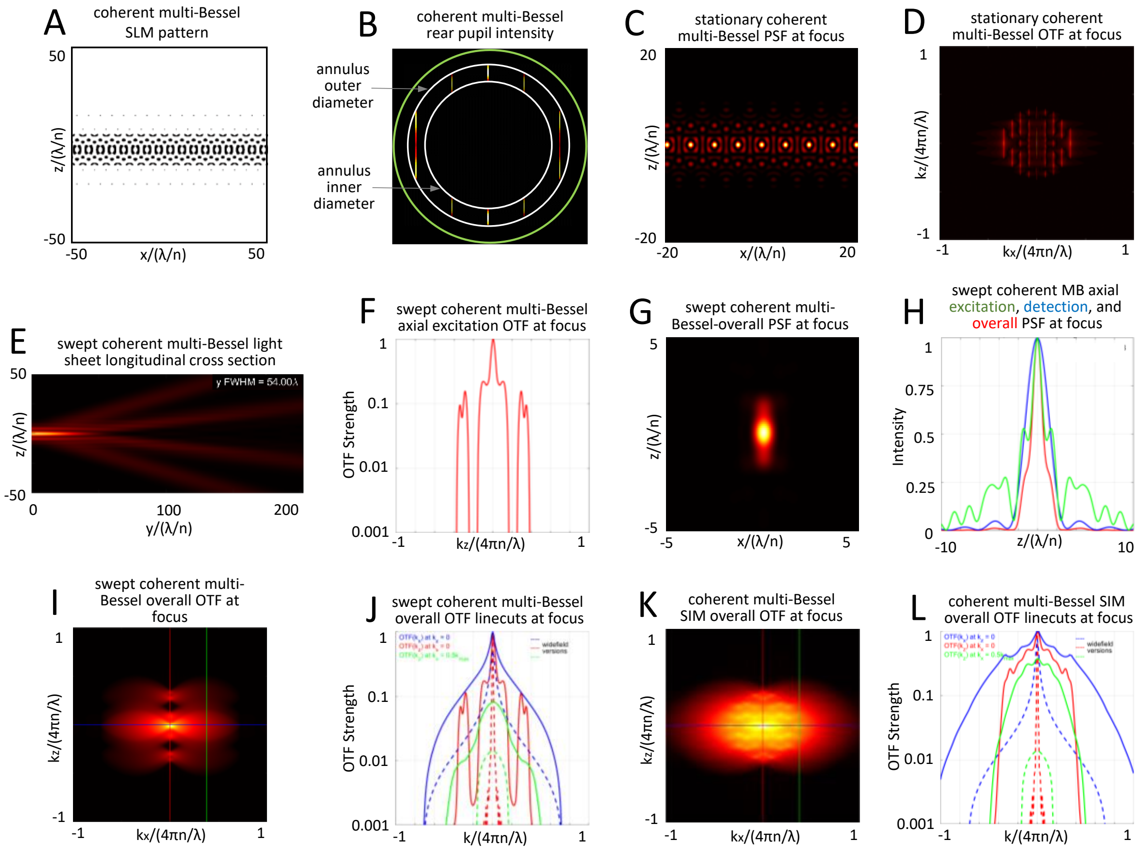

Fig. S6. Theoretical characteristics of a light sheet created by the coherent superposition of an infinite 1D periodic array of Bessel-like beams. $NA_{annulus} = 0.50/0.40$, $y_{FWHM} = 54.0$ λ_{exc}/n, and period $T = 2\lambda_{exc}/NA_{exc}$ where $NA_{exc} = 0.45$. The panels show:

(A) Binary phase pattern $\Phi_{SLM}(x, z)$ applied to the SLM to create the desired axially bound coherent multi-Bessel pattern which is swept or stepped (SIM mode) to create the light sheet.

(B)-(J) Same descriptions as in Fig. S5;

(K) Orthoslice at the excitation focal plane through the overall optical transfer function $OTF_{overall}^{SIM}(k_x, k_z)$ in the structured illumination mode;

(L) Linecuts (solid lines) through $OTF_{overall}^{SIM}(k_x, k_z)$ at the locations shown in **(K)**. Also included are linecuts through $OTF_{det}(\boldsymbol{k})$ as described for Fig. S5J.

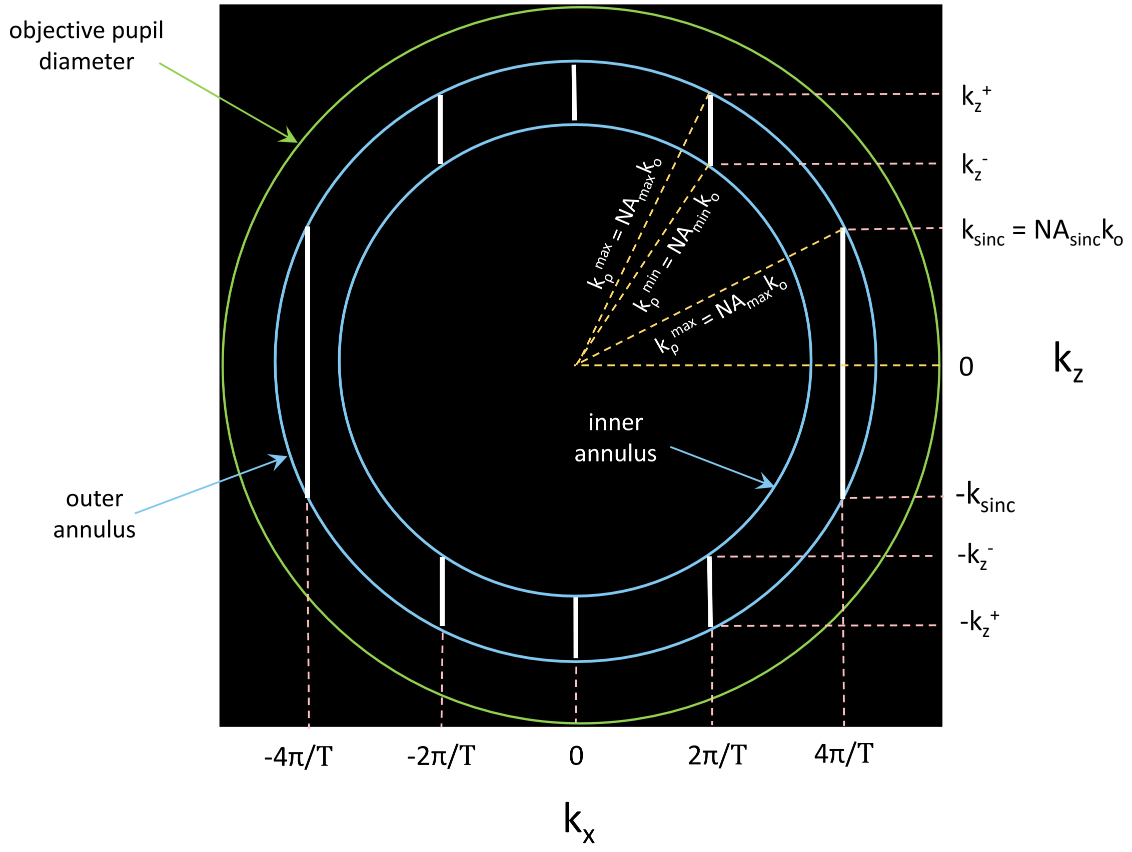

Fig. S7. **Parameters relevant to finding analytic expressions for the contribution of each pupil band to the cross sectional PSFs of sinc, cosine-sinc, or ideal coherent multi-Bessel light sheets.**

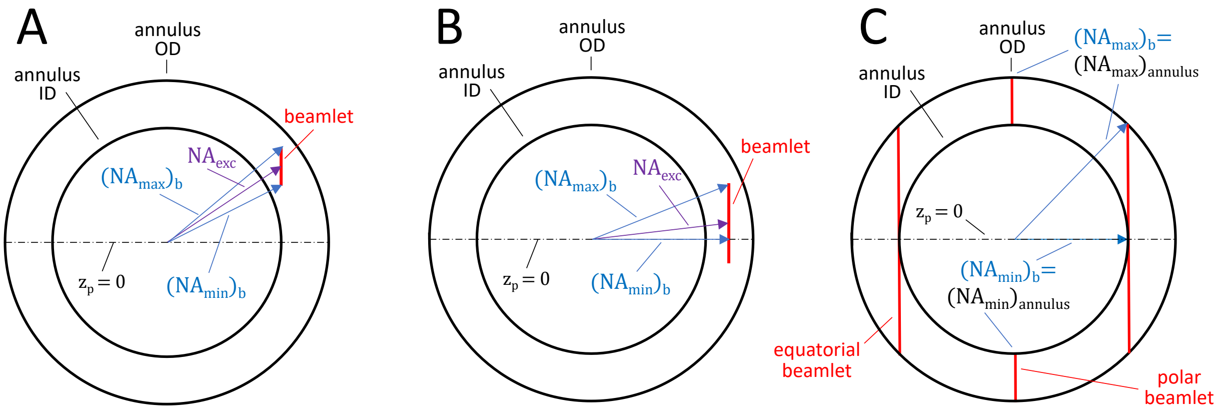

Fig. S8. Maximum and minimum numerical apertures $(NA_{max})_b$ and $(NA_{min})_b$ at the extrema of beamlets at different locations within the annular transmission region in the rear pupil.

(A) Beamlet fully above the $z_p = 0$ line;

(B) beamlet that straddles the $z_p = 0$ line;

(C) condition in a square lattice where the equatorial beamlets are tangent to the inner diameter of the annulus so that their $(NA_{max})_b$ and $(NA_{min})_b$ are identical to those of the polar beamlets.

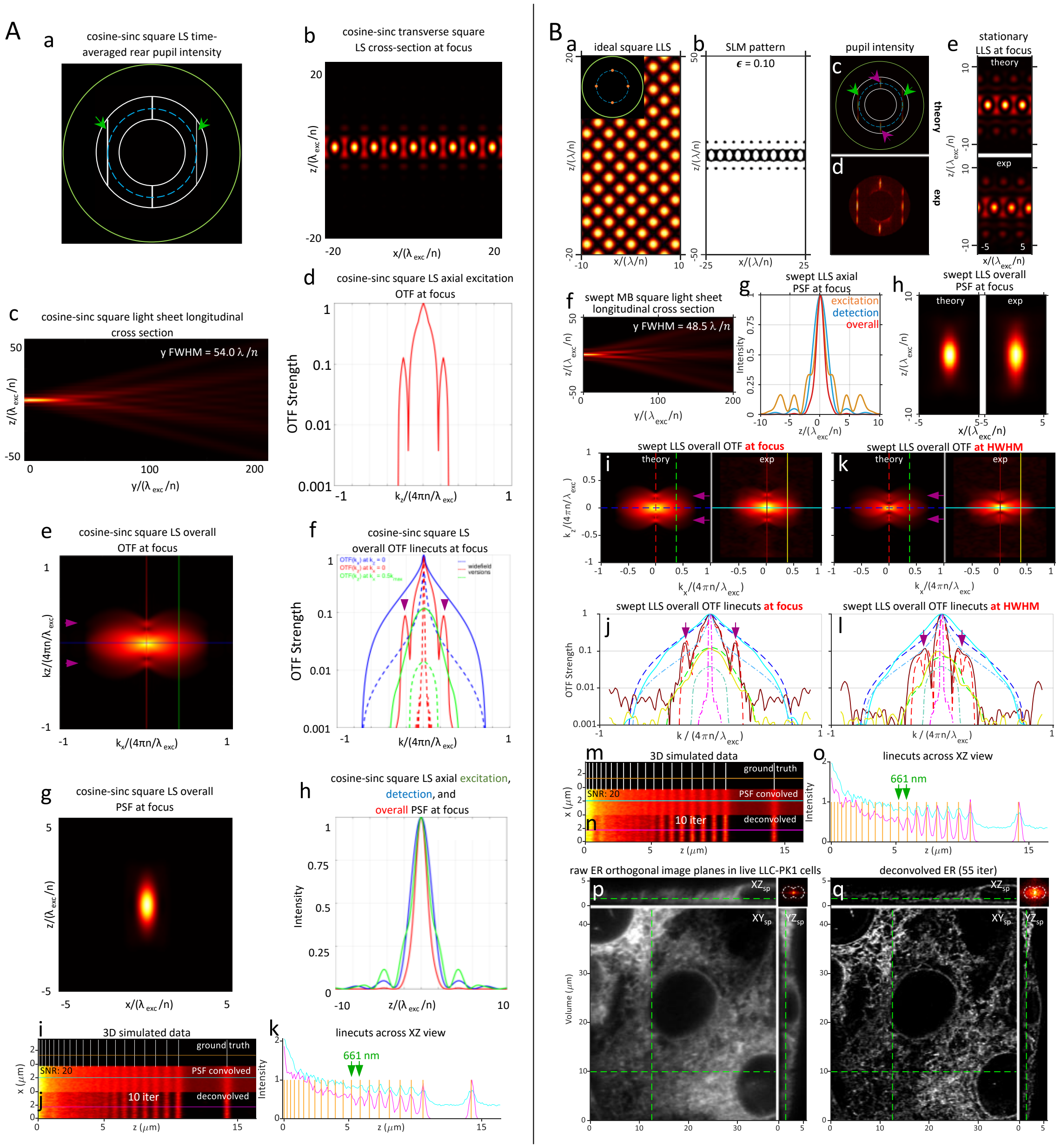

Fig. S9. Comparison of methods to generate a swept square lattice light sheet.

(A) Theoretical characteristics of a cosine-sinc light sheet generated by field synthesis (*15*), designed to mimic a swept lattice light sheet of square symmetry. NA_{exc} = 0.30, $NA_{annulus}$ = 0.375/0.225, $y_{FWHM} = 54.0\lambda_{exc}/n$.Subpanels in **(A)** describe:

a) Time-integrated pupil pattern after serial illumination of each pupil band;

b) Cross-sectional intensity $I_{exc}^{fixed}(x,z)$ of the LLS at the excitation focal plane that would exist if all bands in a) were illuminated simultaneously;

c) Longitudinal orthoslice $I_{exc}^{swept}(y,z)$ through the time-integrated light sheet;

d) Linecut through the time averaged excitation transfer function $OTF_{exc}^{swept}(k_x,k_z)$ at the excitation focal plane;

e) Orthoslice through the overall swept transfer function $OTF_{overall}^{swept}(k_x, k_z)$ at the excitation focal plane;

f) Linecuts (solid lines) through $OTF_{overall}^{swept}(k_x, k_z)$ at the locations shown in e). Also included for reference (dot-dash lines) are linecuts through $OTF_{det}(\boldsymbol{k})$ at the same locations, i.e., the k_x and k_z axes, as well as the line $k_x = 2\pi NA_{det}/\lambda_{det}$ where the widefield microscope has its highest resolution in z;

g) Orthoslice through the overall point spread function $PSF_{overall}^{swept}(\boldsymbol{x})$ of the light sheet at the excitation focal plane;

h) Axial linecuts at the focal plane through: the light sheet in c) (green); the point spread function $PSF_{det}(\boldsymbol{x})$ of the objective orthogonal to the light sheet used to image the emitted fluorescence (blue); and the overall $PSF_{overall}^{swept}(\boldsymbol{x})$ (red);

i) Test pattern of lines of variable spacing for simulated imaging;

j) Simulated RL-deconvolved image of the pattern in j) after imaging with $PSF_{overall}^{swept}(\boldsymbol{x})$ from g);

k) Linecuts through the test pattern (orange) and corresponding raw (aqua) and RL deconvolved (magenta) images. Green arrows show the narrowest resolved line pair;

(B) Theoretical and experimentally measured characteristics of a multi-Bessel LLS having square symmetry. $NA_{exc} = 0.30$, $NA_{annulus} = 0.375/0.225$, cropping factor $\epsilon = 0.10$, and $y_{FWHM} = 48.5\ \lambda_{exc}/n$. Panel descriptions are the same as in Fig. S3.

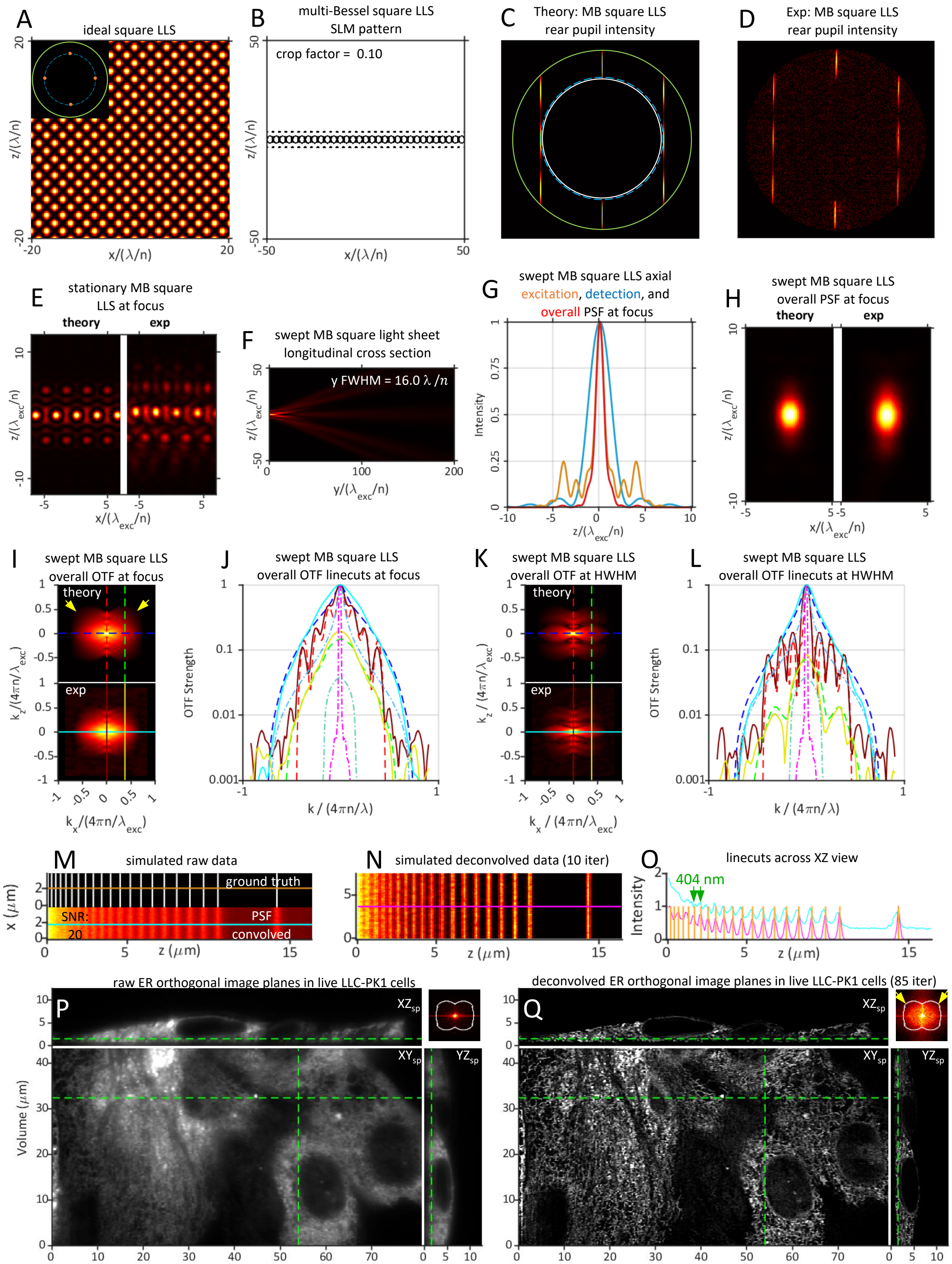

Fig. S10. Theoretical and experimentally measured characteristics of a short (y_{FWHM} = 16.0 λ_{exc}/n) multi-Bessel square LLS. $NA_{exc} = 0.41$, $\epsilon = 0.10$, and $NA_{annulus} = 0.60/0.40$. Panel descriptions are the same as in Fig. S3.

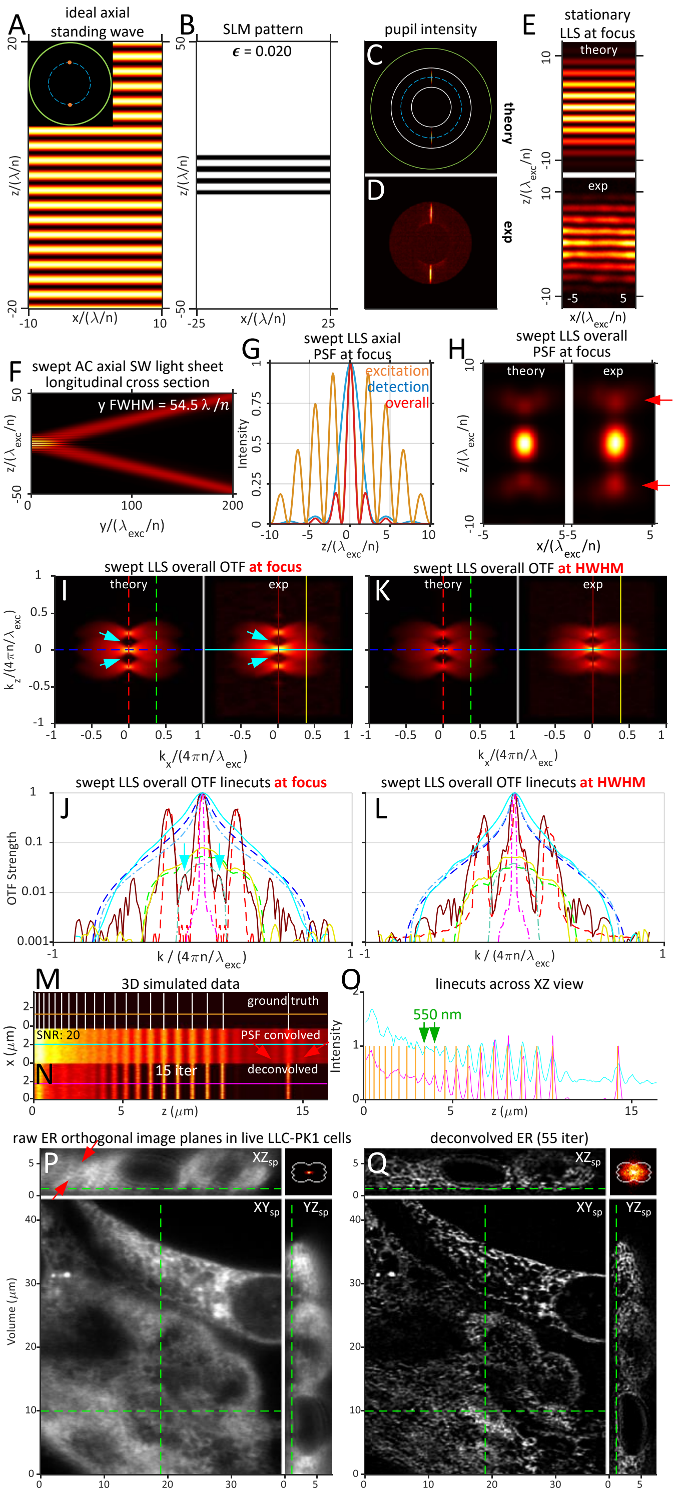

Fig. S11. **Theoretical and experimentally measured characteristics of an axial standing wave light sheet.** $NA_{exc} = 0.30$, $\epsilon = 0.020$, and $y_{FWHM} = 54.5\ \lambda_{exc}/n$ axially confined by a Gaussian bounding function of $\sigma_{NA} = 0.10$ and filtered by an annulus of $NA_{annulus} = 0.40/0.20$. Panel descriptions are the same as in Fig. S3.

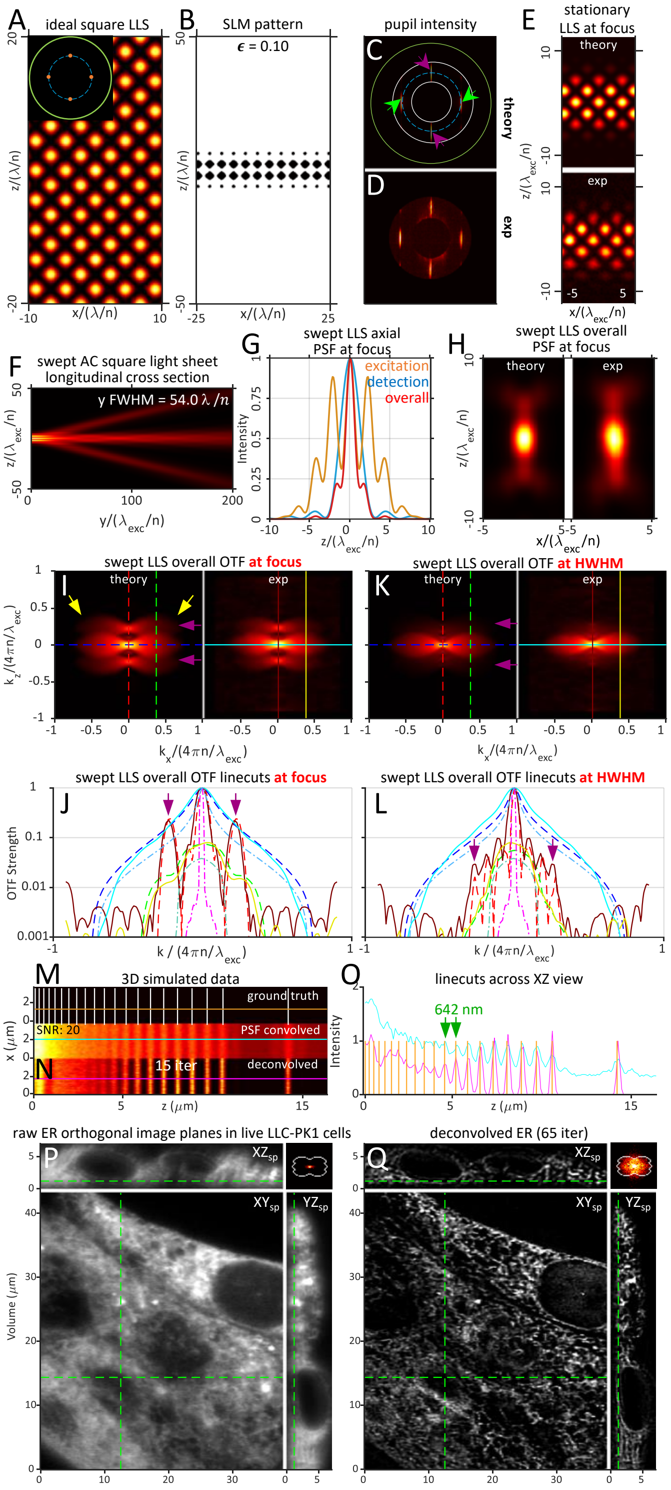

Fig. S12. **Theoretical and experimentally measured characteristics of an axially confined square LLS.** $NA_{exc} = 0.30$, $\sigma_{NA} = 0.090$, $\epsilon = 0.10$, $NA_{annulus} = 0.40/0.20$, and $y_{FWHM} = 54.0$ λ_{exc}/n. Panel descriptions are the same as in Fig. S3.

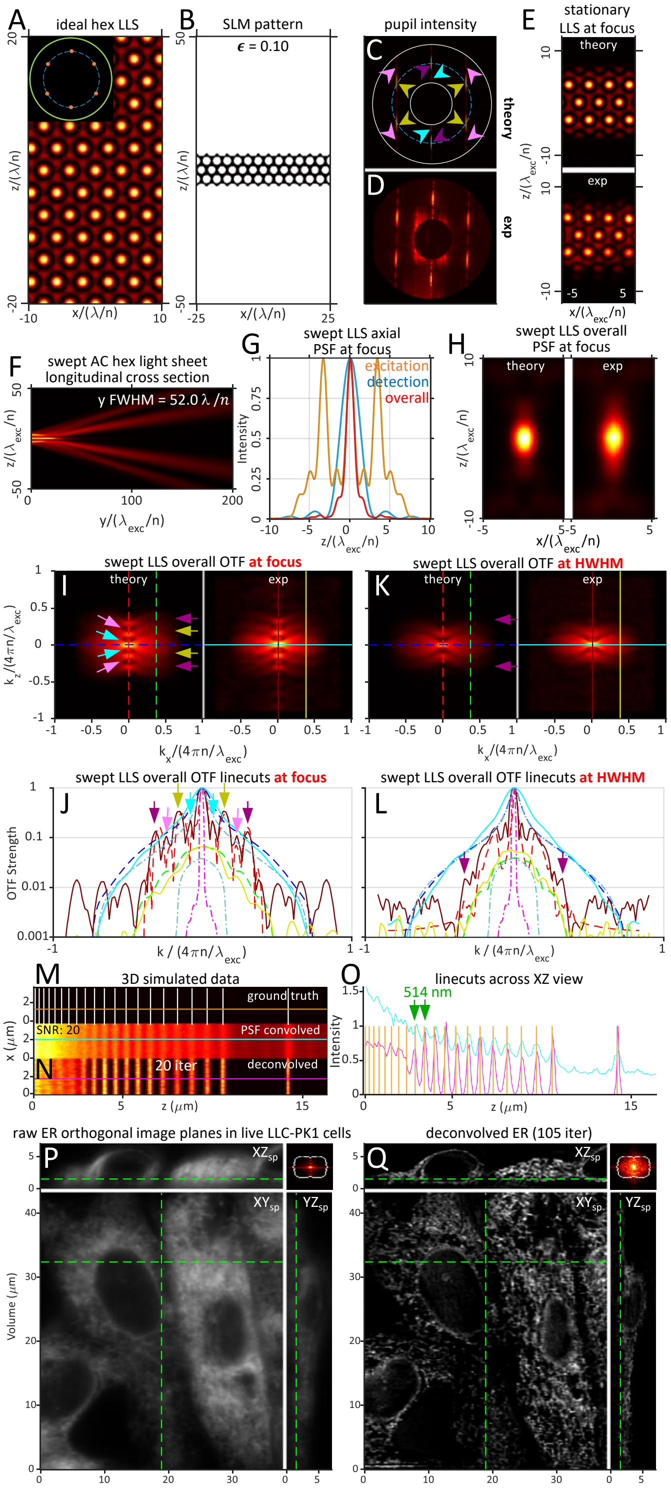

Fig. S13. Theoretical and experimentally measured characteristics of an axially confined hexagonal LLS. $NA_{exc} = 0.40$, $\sigma_{NA} = 0.075$, $\epsilon = 0.10$, $NA_{annulus} = 0.60/0.20$, and $y_{FWHM} = 52.0\ \lambda_{exc}/n$. Panel descriptions are the same as in Fig. S3.

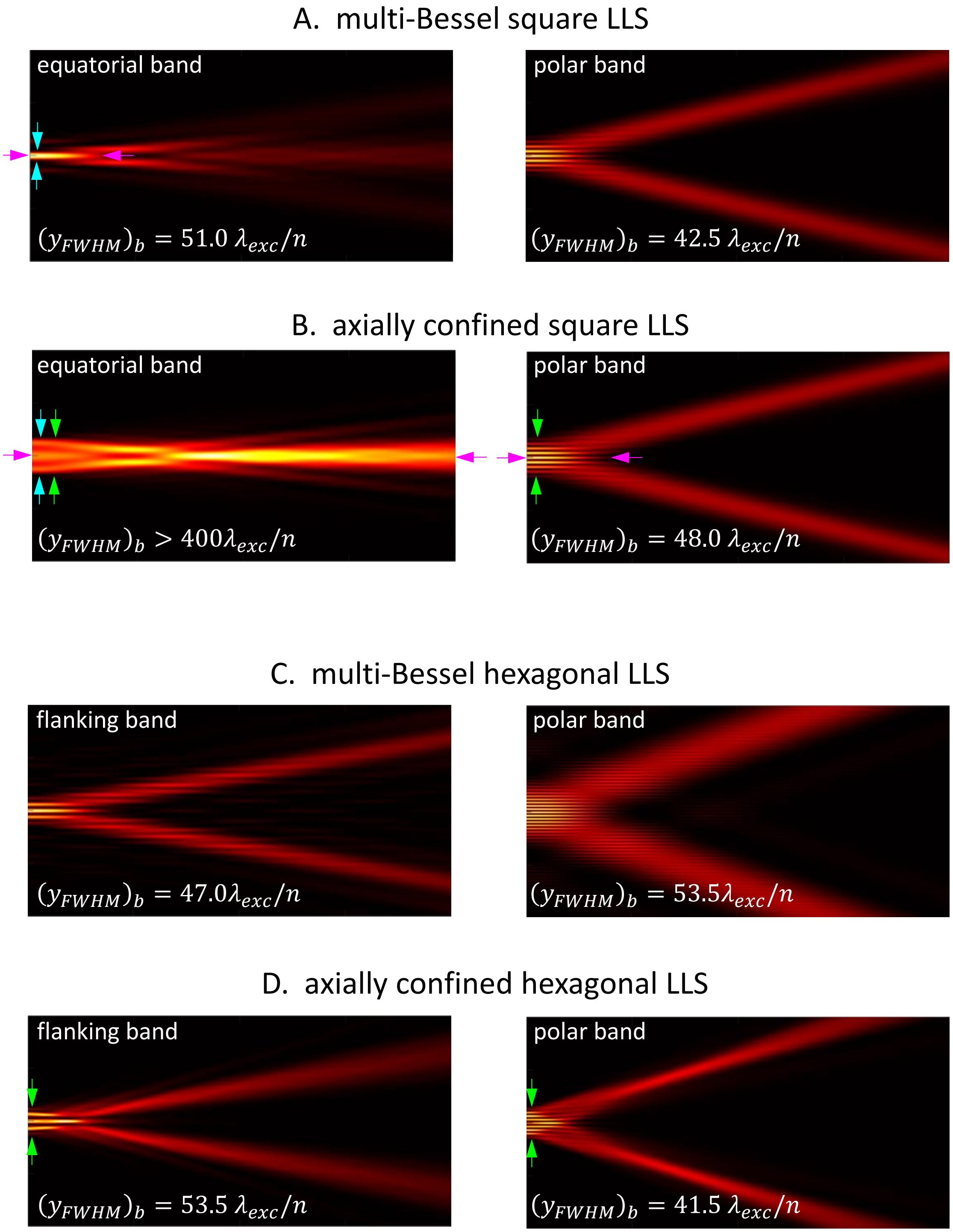

Fig. S14. Comparisons of the longitudinal propagation in the $yz_{optical}$ plane for the individual pupil bands of four different LLSs.
(A) multi-Bessel square LLS of Fig. S9B; **(B)** axially confined square LLS of Fig. S12; **(C)** multi-Bessel hexagonal LLS of Fig. 2B; and **(D)** axially confined hexagonal LLS of Fig. S13.

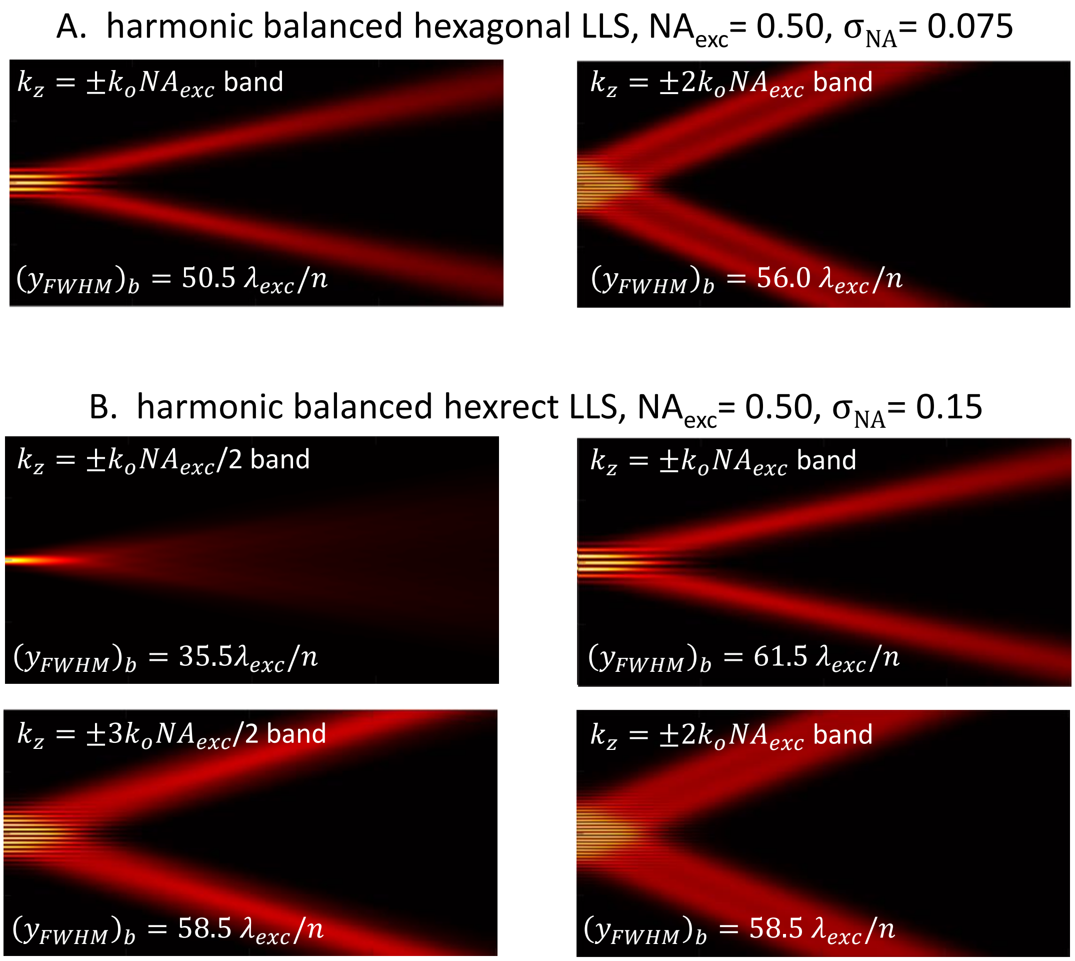

Fig. S15. Longitudinal propagation in the $yz_{optical}$ plane of the individual bands of two harmonic balanced LLSs. (A) the harmonic balanced hexagonal LLS of Fig. 3A; **(B)** the harmonic balanced hexrect pattern of Fig. 3B.

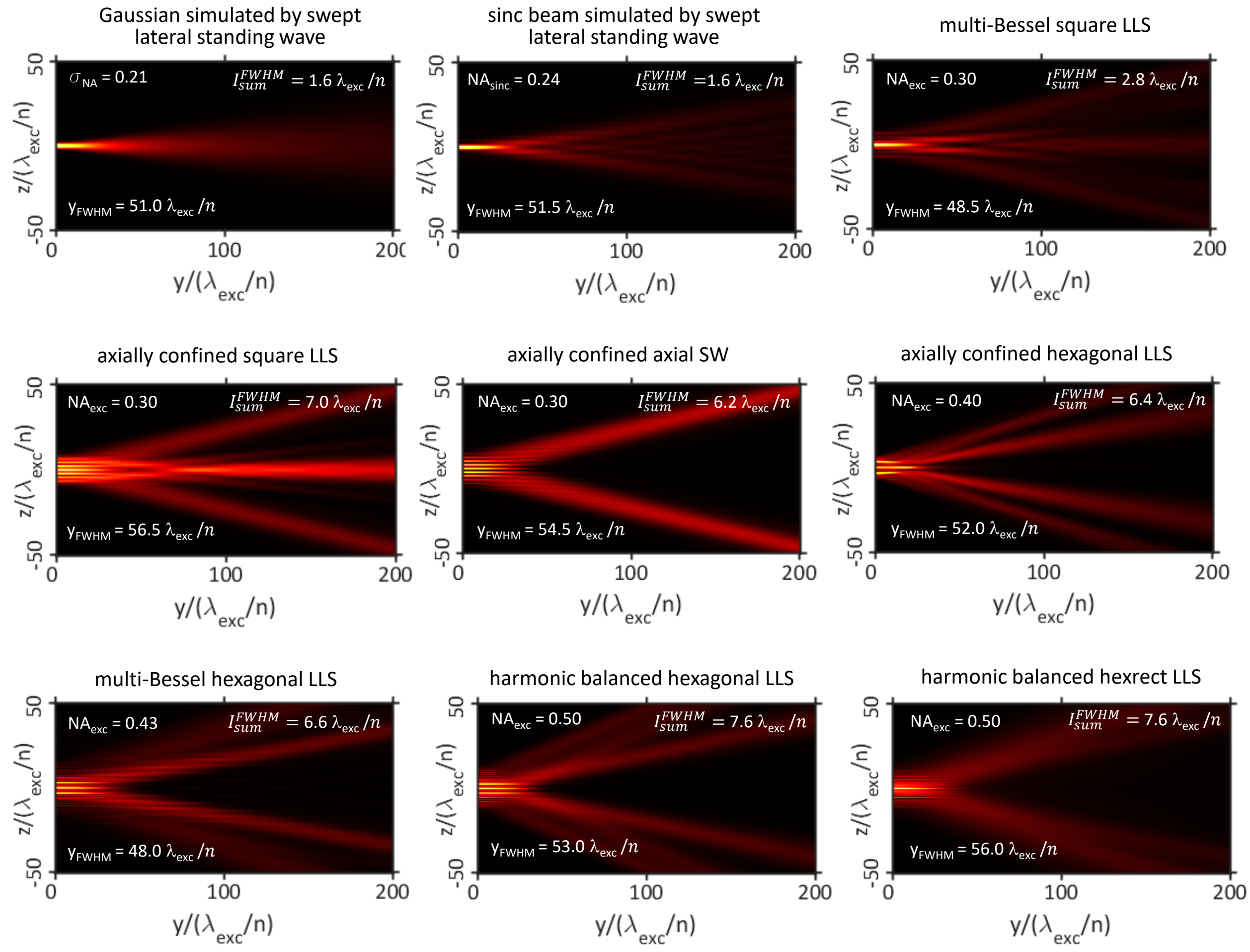

Fig. S16. **Comparisons of the longitudinal propagation in the $yz_{optical}$ plane and the axial confinement I_{sum}^{FWHM} of the excitation at the focal plane.** Light sheets shown are the Gaussian (Fig. S3), sinc (Fig. S4) and seven LLSs in Figs. 2, 3, S9B, and S11-13, all of length $y_{FWHM} \sim$ 50 λ_{exc}/n.

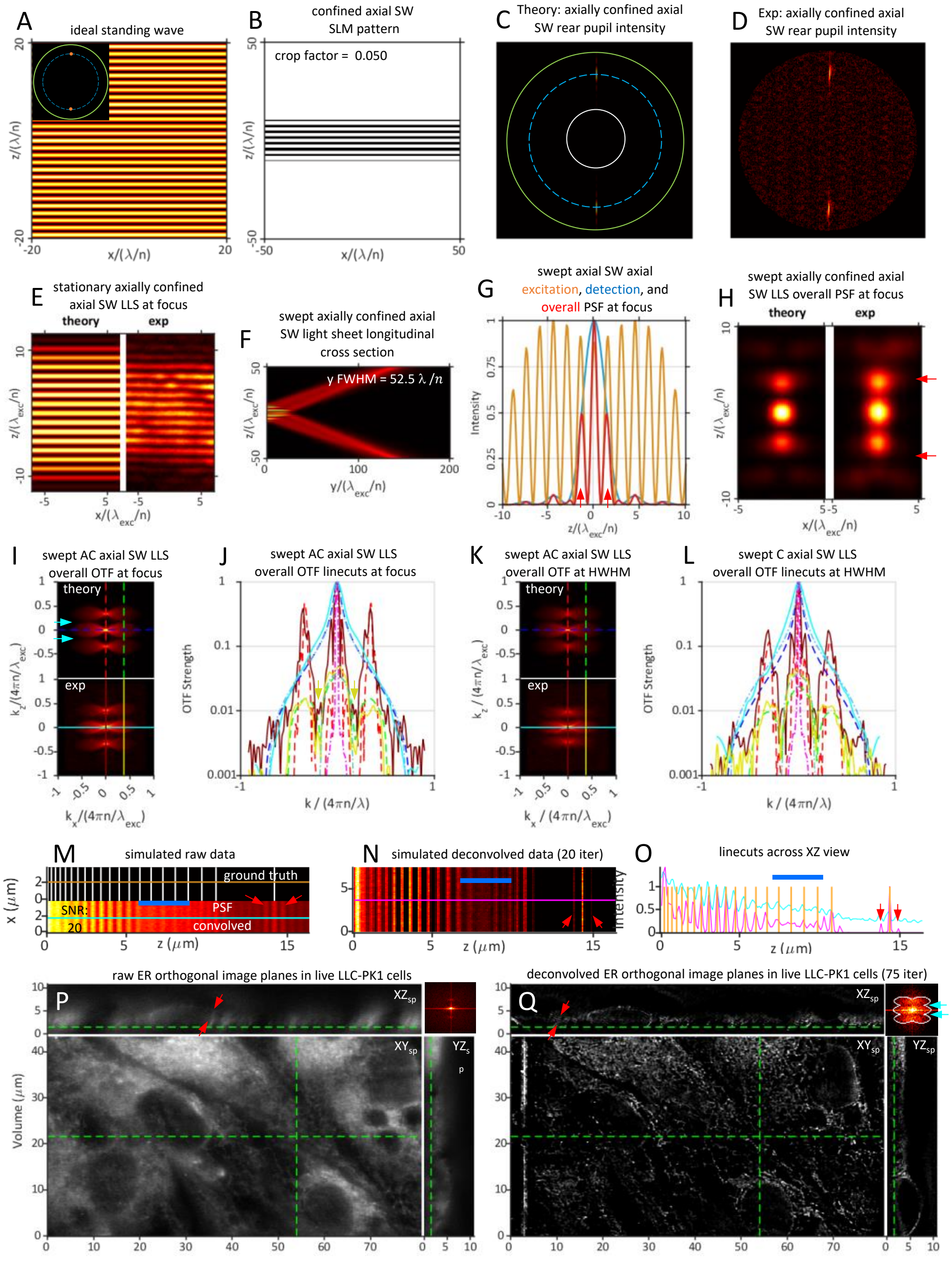

Fig. S17. **Theoretical and experimentally measured characteristics of a swept axially confined axial standing wave light sheet.** $NA_{exc} = 0.45$, $\sigma_{NA} = 0.13$, $\epsilon = 0.065$, $NA_{annulus} = 0.60/0.20$, and $y_{FWHM} = 52.5\ \lambda_{exc}/n$. Panel descriptions are the same as in Fig. S3. This light sheet produces a discontinuous overall OTF that leads to bands of missing spatial frequencies and post-deconvolution artifacts.

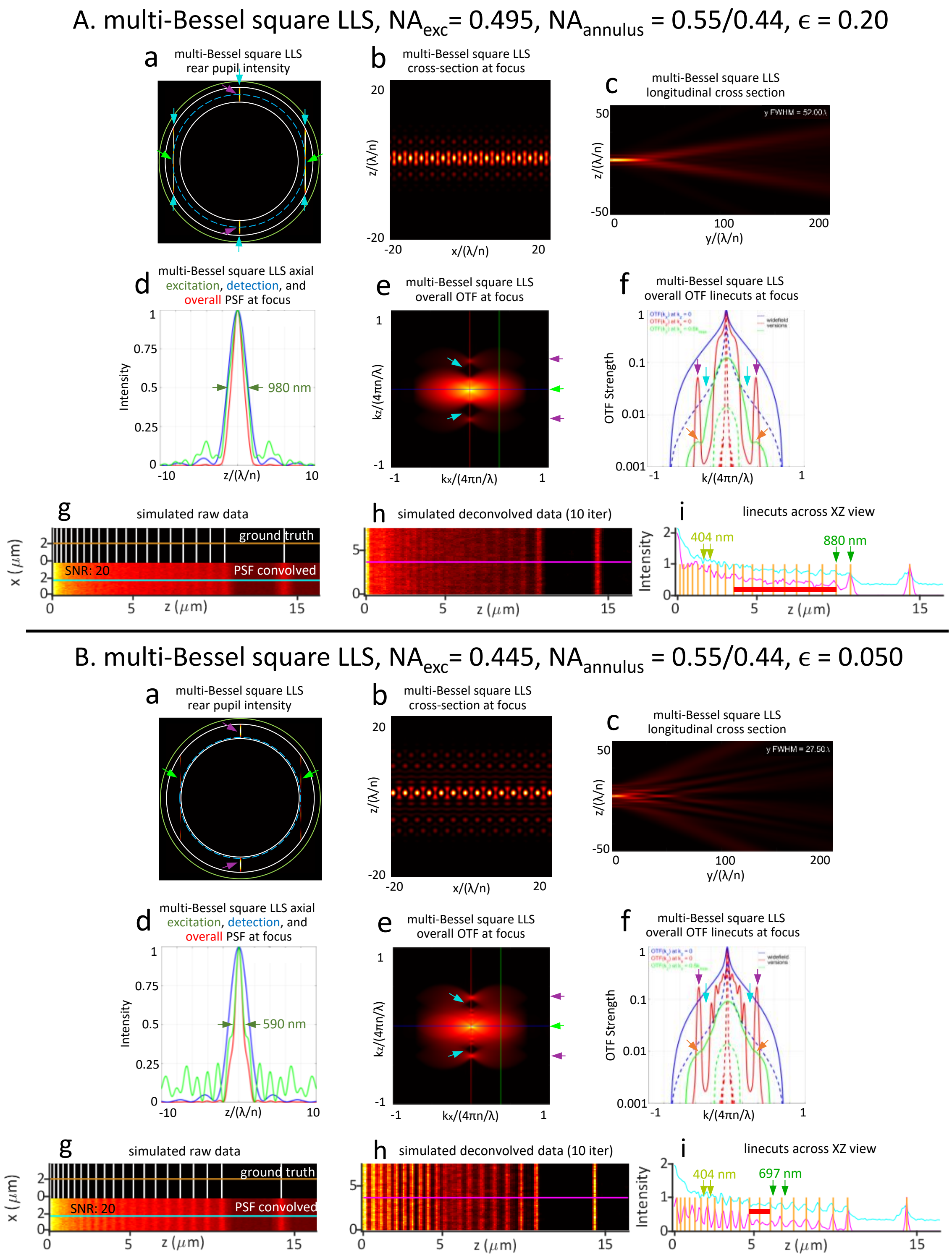

Fig. S18. Theoretical comparison of two multi-Bessel square lattice light sheets having the same $NA_{annulus} = 0.55/0.44$ but substantially different overall OTFs and simulated resolution. (A) LLS with $NA_{exc} = 0.495$, $\epsilon = 0.20$, and $y_{FWHM} = 52.0\ \lambda_{exc}/n$, similar to the conditions in (*8*). **(B)** LLS with $NA_{exc} = 0.445$, $\epsilon = 0.050$, and $y_{FWHM} = 27.5\ \lambda_{exc}/n$, similar to the conditions in (*11*). Panel descriptions are the same as in Fig. S9A.

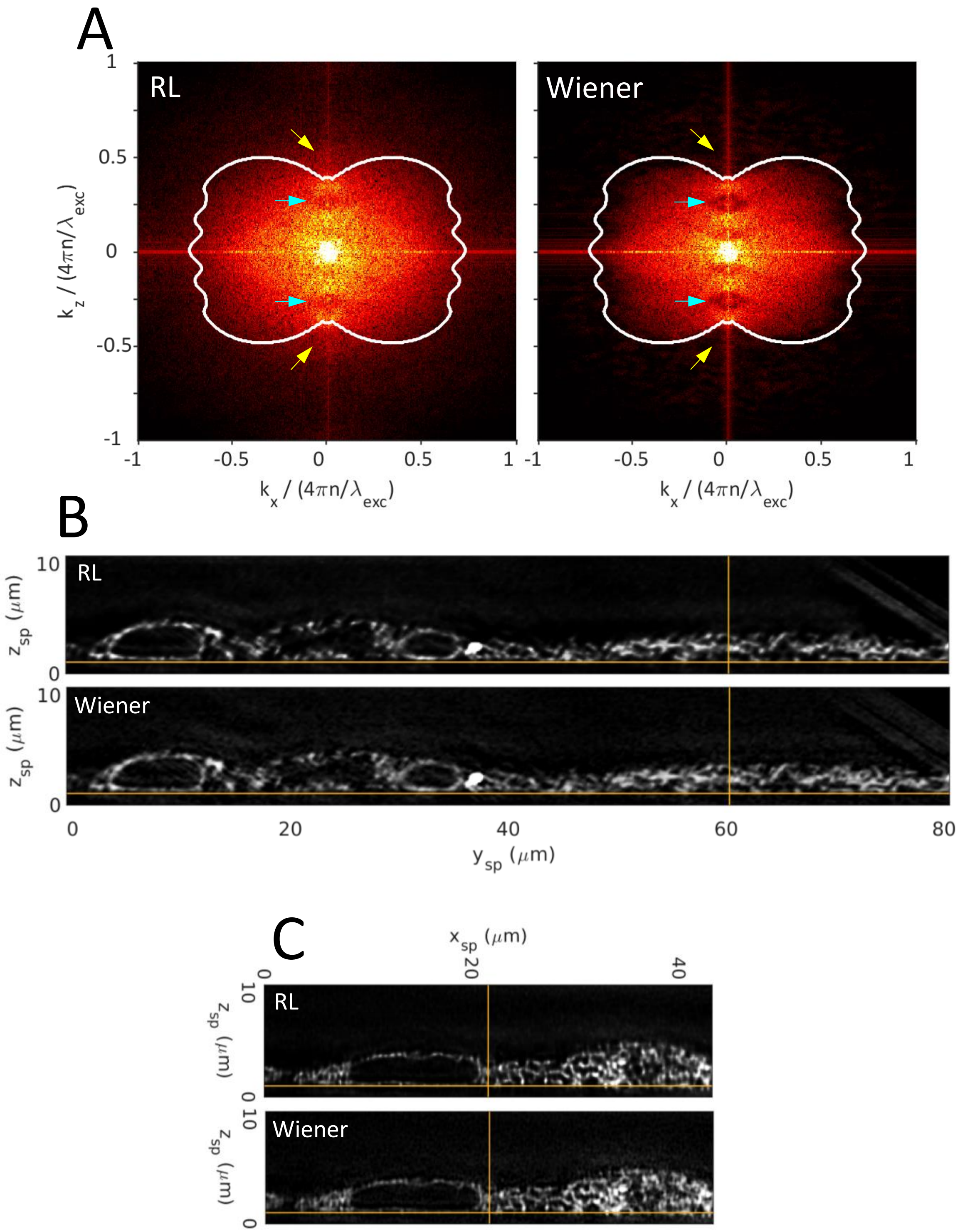

Fig. S19. Comparison of RL and Wiener deconvolution of a live cell image volume acquired with the multi-Bessel hexagonal light sheet of Fig. 2B. (A) FFT of the post-deconvolution images; **(B,C)** orthoslices in the $xz_{specimen}$ and $yz_{specimen}$ planes, respectively, through the deconvolved image volumes.

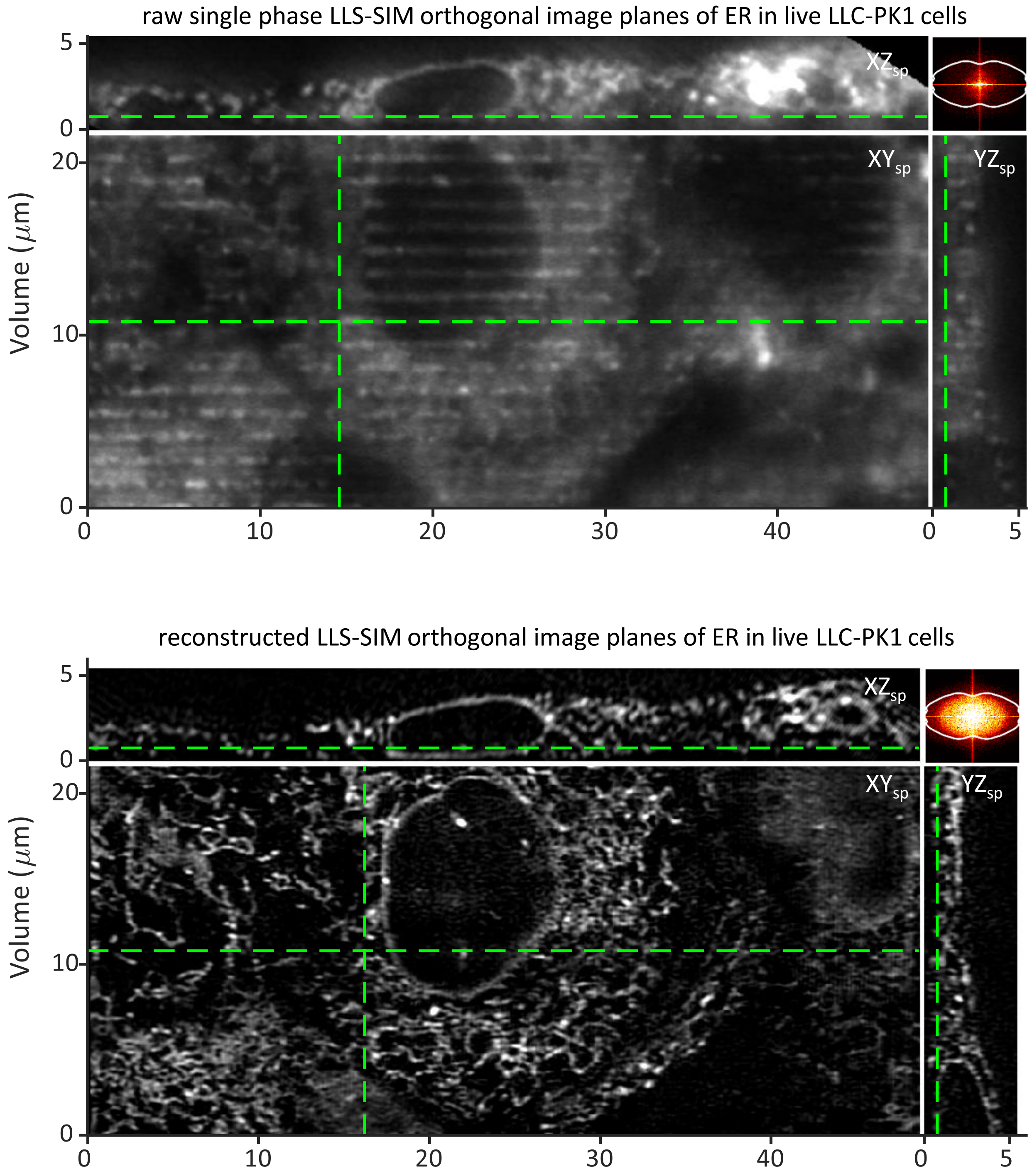

Fig. S20. **Raw and reconstructed image planes of the ER in living LLC-PK1 cells acquired with the structured illumination mode of LLSM.** Acquired using a harmonic balanced hexagonal lattice light sheet of $NA_{exc} = 0.46$, $\sigma_{NA} = 0.10$, $NA_{annulus} = 0.60/0.20$, and $y_{FWHM} = 42.5\ \lambda_{exc}/n$.

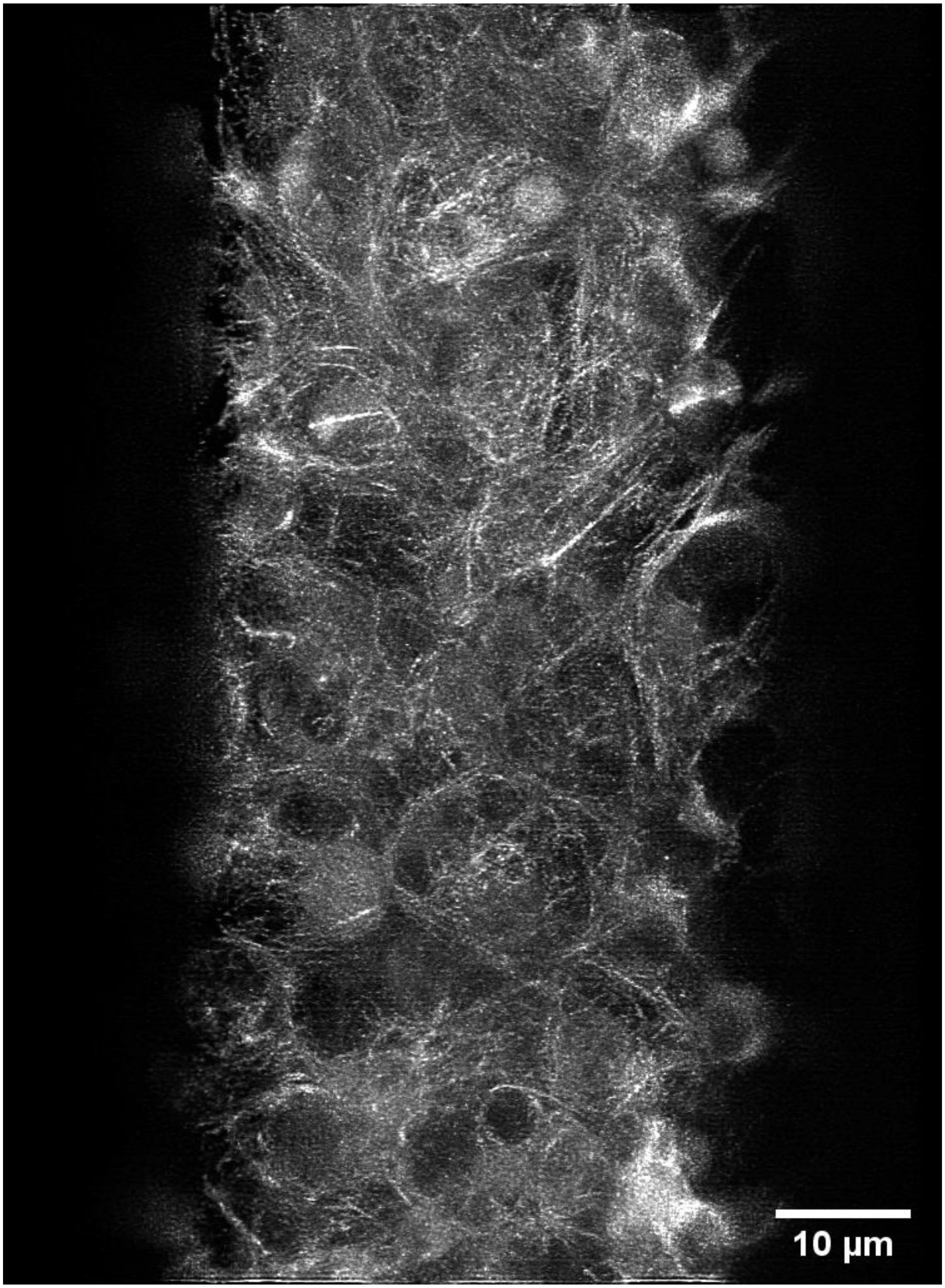

Fig. S21. **Maximum intensity projection of confluent human induced pluripotent stem cells expressing a tubulin marker of the type used for the photobleaching comparisons.**

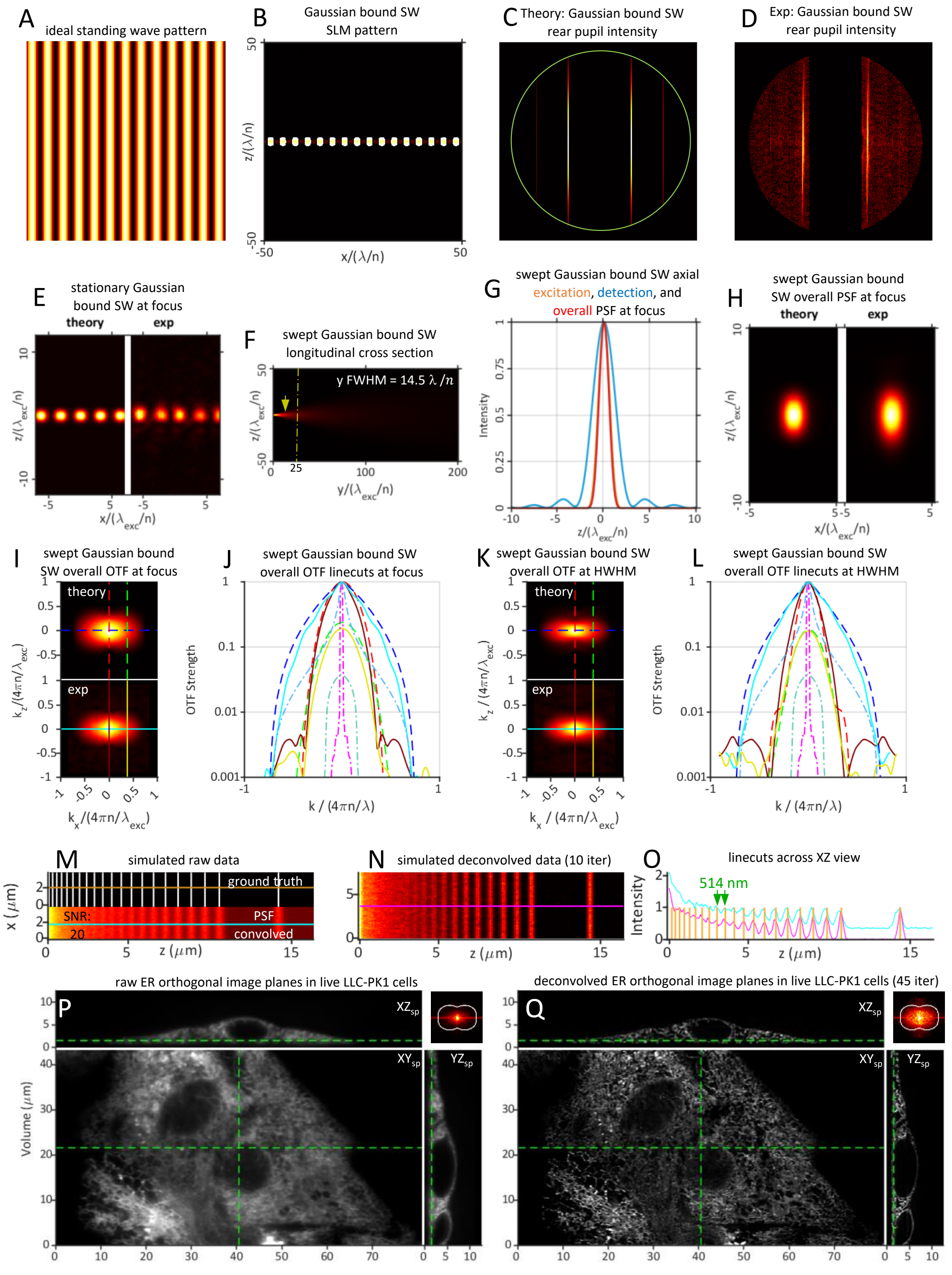

Fig. S22. **Theoretical and experimentally measured characteristics of a swept Gaussian light sheet of propagation length $y_{FWHM} = 14.5\ \lambda_{exc}/n$.** Light sheet created by a swept lateral standing wave of $NA_{exc} = 0.21$ having a Gaussian bounding envelope in k_z of $\sigma_{NA} = 0.42$, filtered by a pupil conjugate annulus of $NA_{annulus} = 0.60/0.20$. Given the short propagation range, the image volume in panels P and Q was constructed by stitching four tiled subregions in the $\hat{\boldsymbol{e}}_{z_{specimen}}$ direction. Panel descriptions are the same as in Fig. S3.

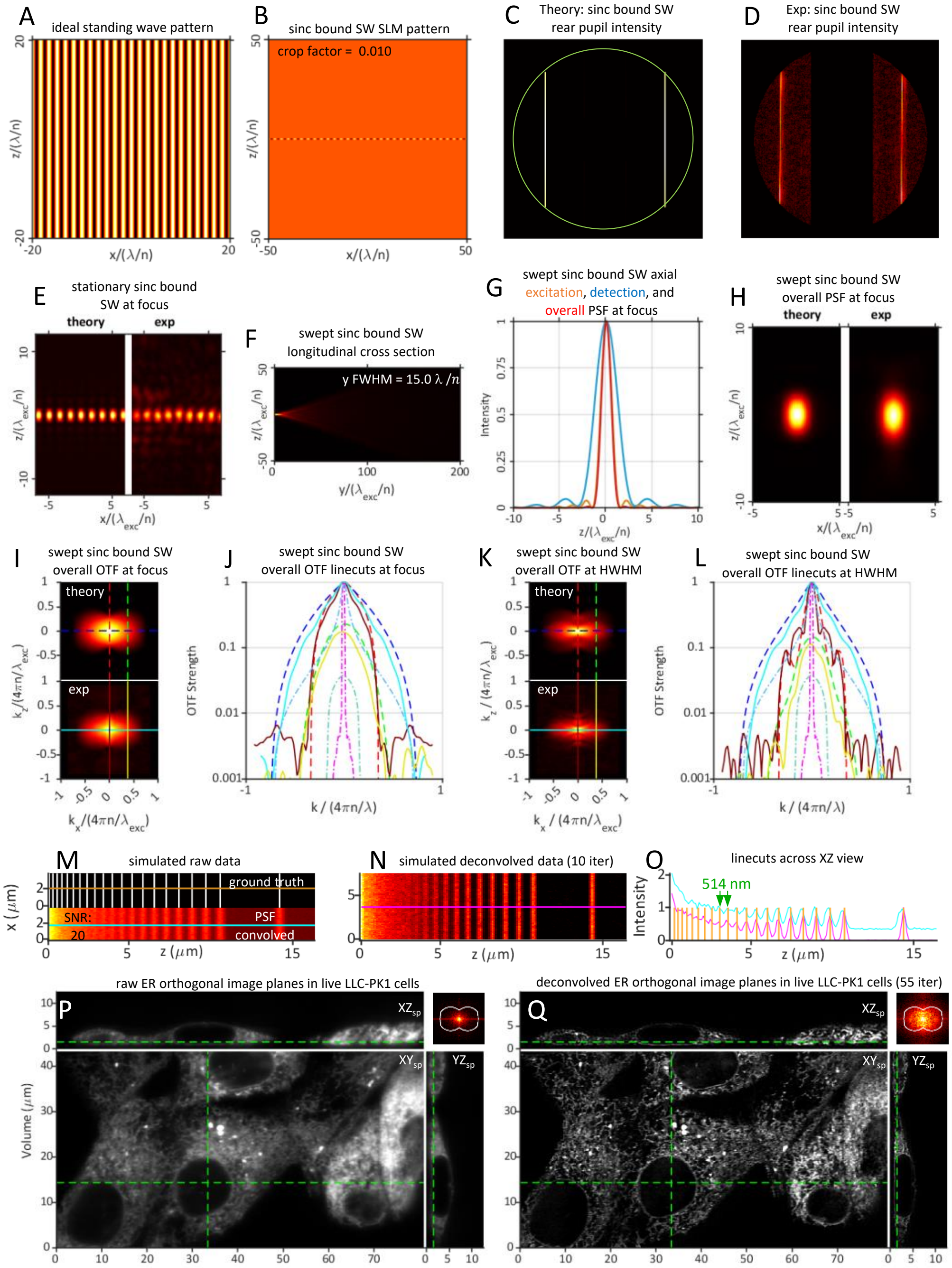

Fig. S23. **Theoretical and experimentally measured characteristics of a swept sinc light sheet of propagation length $\boldsymbol{y_{FWHM} = 15.0\ \lambda_{exc}/n}$.** Light sheet created by a pair of uniformly illuminated equatorial pupil bands of $NA_{exc} = 0.40$ filtered by an annulus of $NA_{annulus} = 0.60/0.20$ to limit the maximum NA in k_z to $NA_{sinc} = 0.45$ for the resulting swept lateral standing wave in the specimen. Given the short propagation range, the image volume in panels P and Q was constructed by stitching four tiled subregions in the $\hat{\boldsymbol{e}}_{\boldsymbol{z_{specimen}}}$ direction. Panel descriptions are the same as in Fig. S3.

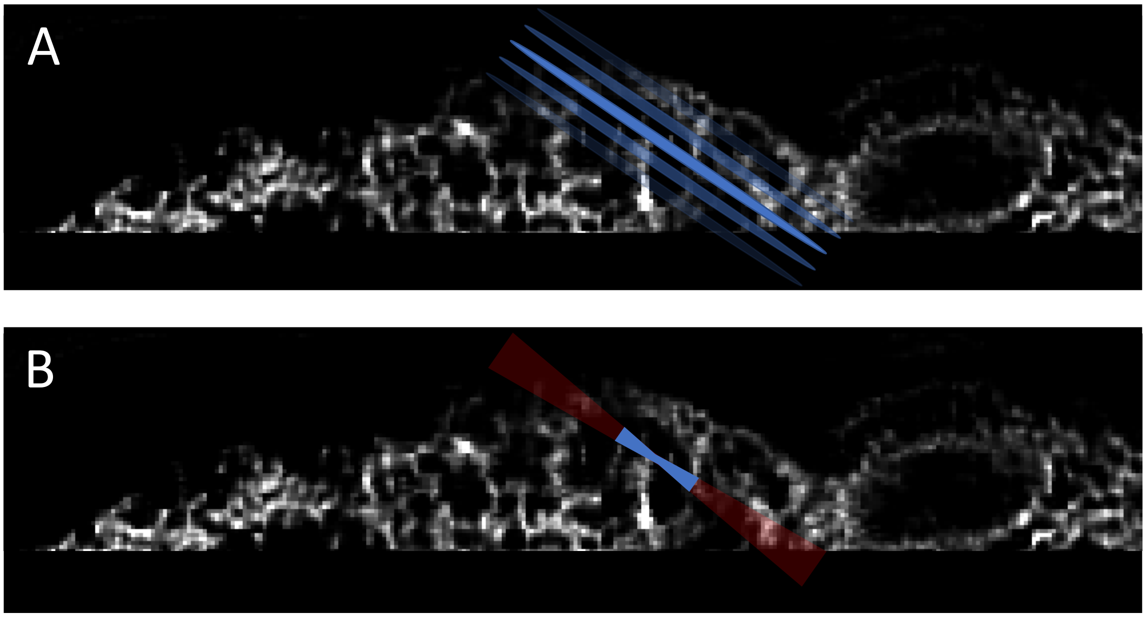

Fig. S24. **Illustration of regions of usable signal and unusable background in lattice and conventional light sheets. (A)** central peak (opaque blue) and sidelobe (translucent blue) fluorescence from a LLS that, within its propagation range, is efficiently collected and reassigned to its original source via deconvolution. **(B)** collimation region (opaque blue) and divergent region (translucent red) fluorescence from a Gaussian or sinc light sheet, the former of which produces useful signal, but the latter of which creates out-of-focus background that must be removed (e.g., by restricting the camera FOV). The longer the specimen along the propagation direction relative to the propagation length, the more fluorescence that must be removed, and the greater the rate of photobleaching.

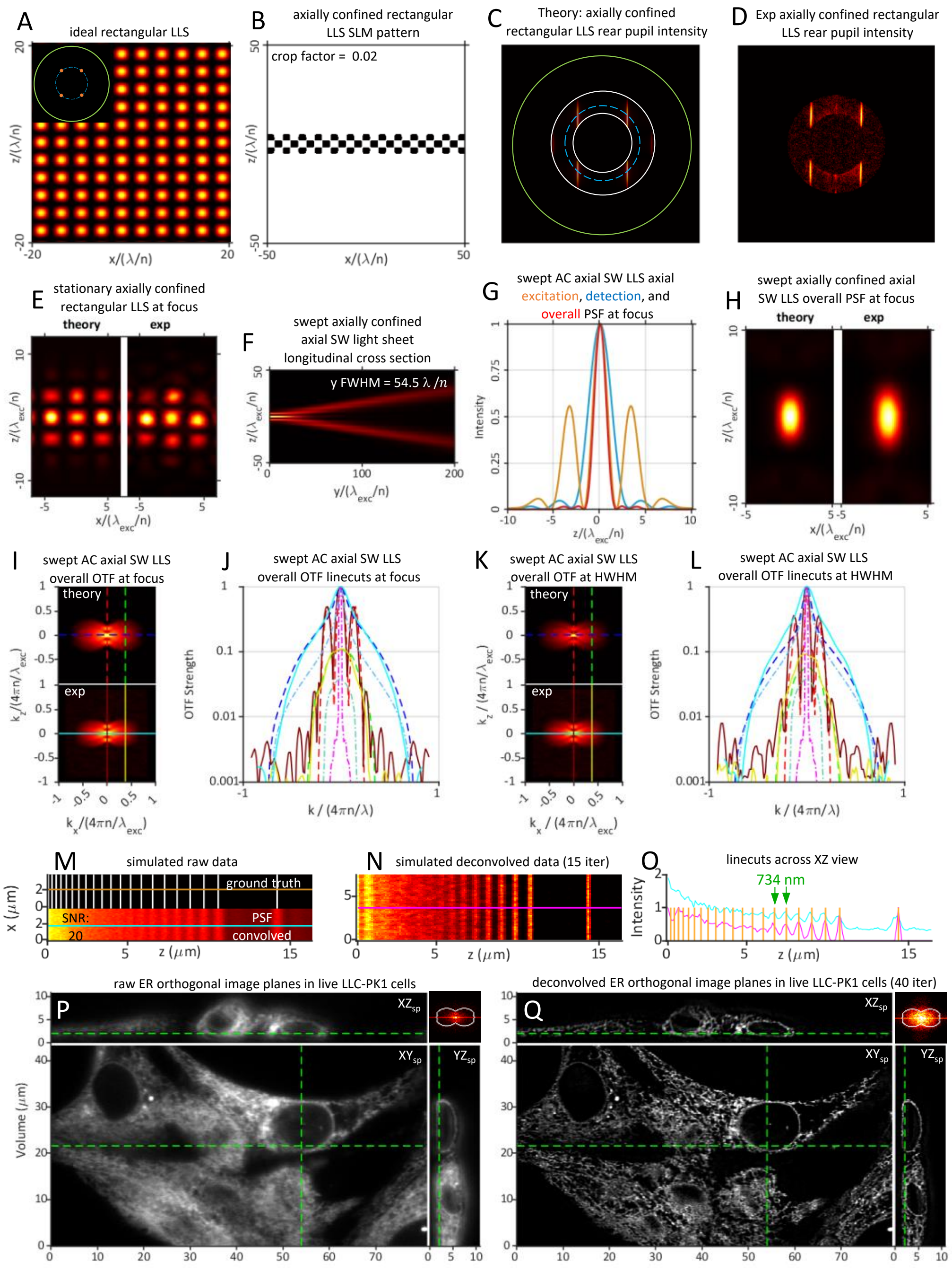

Fig. S25. **Theoretical and experimentally measured characteristics of a swept axially confined axial standing wave light sheet.** $NA_{exc} = 0.25$, $\sigma_{NA} = 0.13$, $\epsilon = 0.020$, $NA_{annulus} = 0.35/0.20$, and $y_{FWHM} = 54.5\ \lambda_{exc}/n$ created from an axially confined rectangular LLS (panel A). Panel descriptions are the same as in Fig. S3.

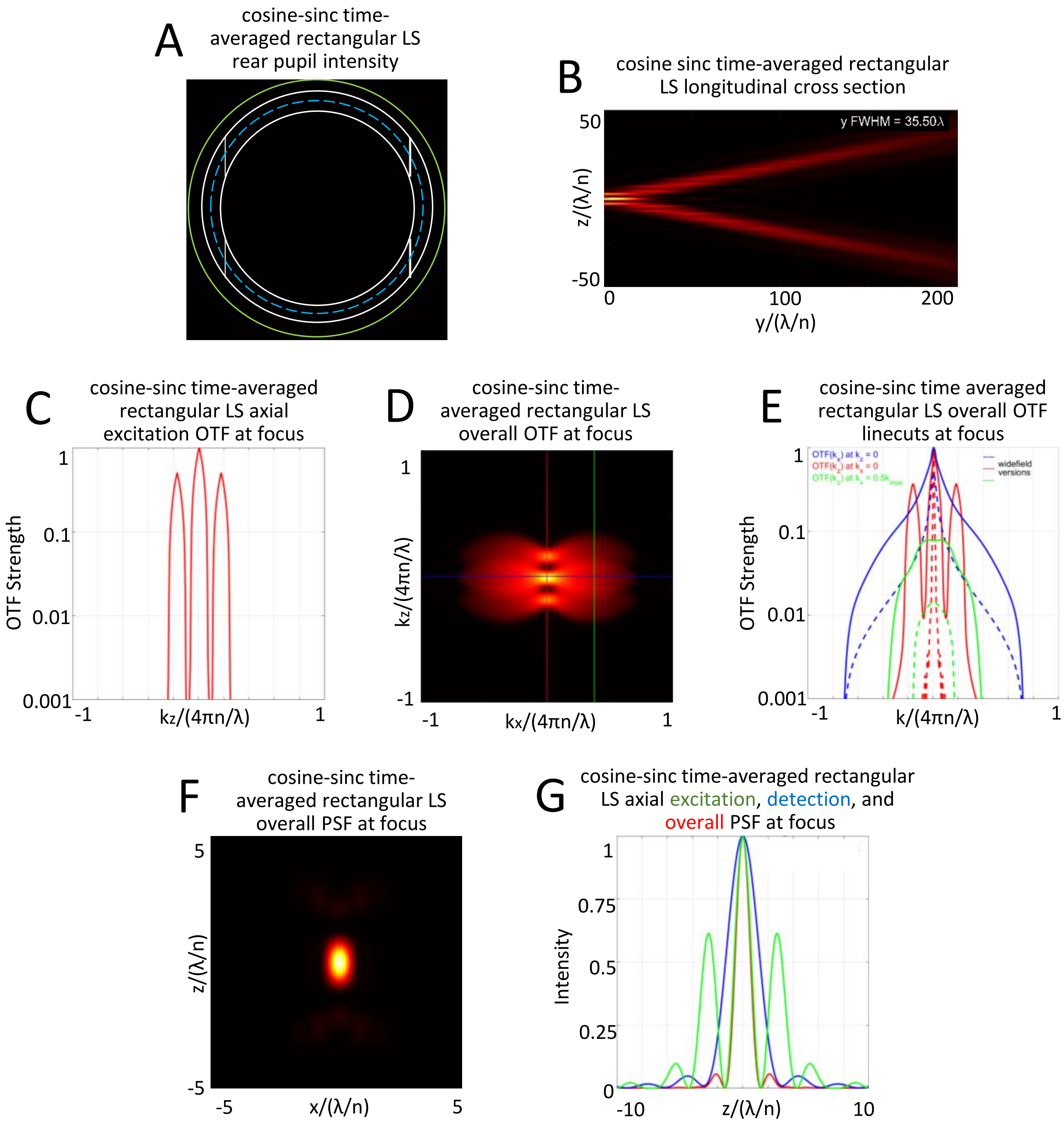

Fig. S26. **Theoretical characteristics of an ideal coherent multi-Bessel LLS used in (*11*).** The LLS is formed from a hexagonal LLS with its polar beamlets removed, resulting in a multi-Bessel rectangular LLS of $NA_{exc} = 0.49$, $NA_{annulus} = 0.536/0.45$, and $y_{FWHM} = 35.5\ \lambda_{exc}/n$.

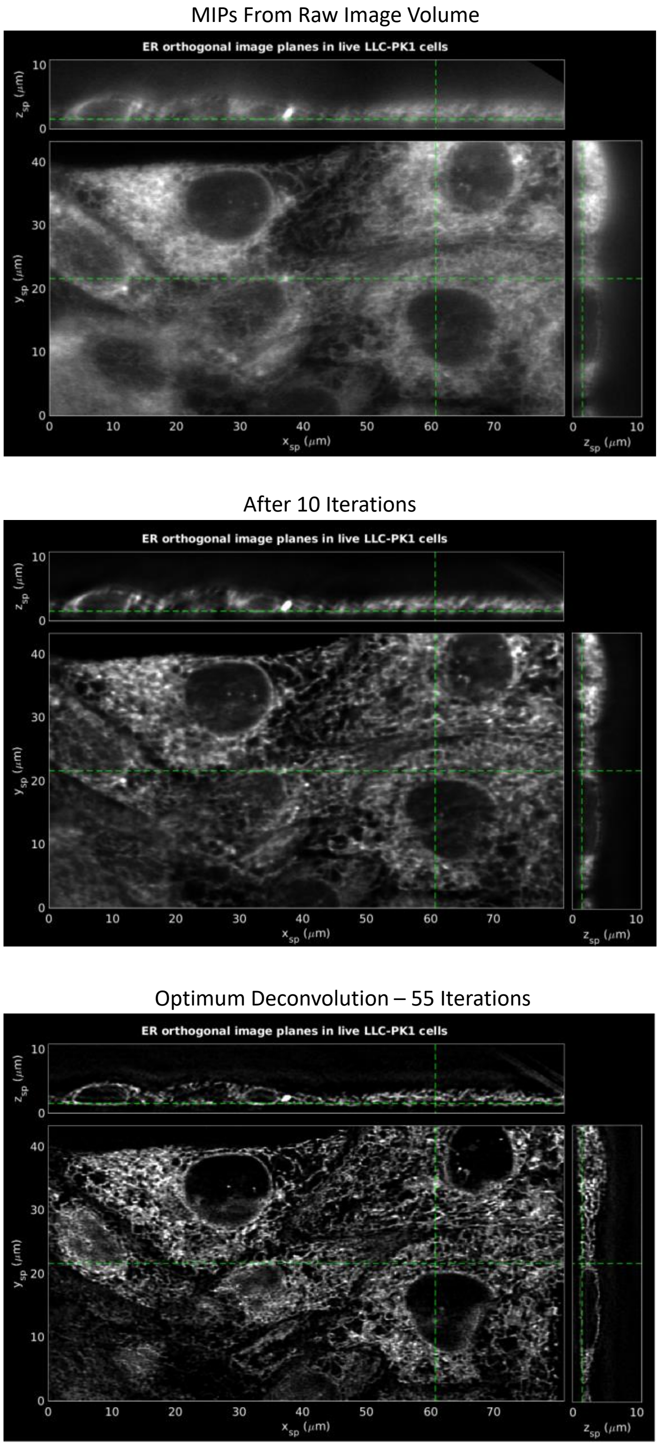

Fig. S27. **Orthoslices through the image volume from the MB hexagonal LLS data in Fig. 2B after 0, 10, and FSC-proscribed 55 iterations**. Out-of-focus background and ghost images collapse to an optimum representation of the sample structure. This process is shown in detail in part 2 of each Movie associated with each light sheet studied here.

Table S1. Theoretical resolution limits of various light sheets.

Light Sheet Description	Gaussian	sinc	multi-Bessel square	axially confined square	axially confined SW	axially confined hexagonal	multi-Bessel hexagonal	harmonic balanced hexagonal	harmonic balanced hexrect	maximum theoretical
$\boldsymbol{NA_{exc}}$	0.21	$NA_{sinc} =$ 0.24	0.30	0.30	0.30	0.40	0.43	0.50	0.50	0.60<br>0.70
$\boldsymbol{\sigma_{NA}}$	0.21	N.A.	N.A.	0.070	0.10	0.075	N.A.	0.075	0.15	N.A.
$\boldsymbol{NA_{annulus}}$	0.40/0.20	0.60/0.20	0.375/0.225	0.40/0.20	0.40/0.20	0.60/0.20	0.47.0.40	0.60/0.40	0.60/0.40	N.A.
	0.00	0.00	0.10	0.10	0.020	0.10	0.080	0.010	0.010	N.A.
$\boldsymbol{R\left(\hat{e}_{y_{optical}}\right) = R\left(\hat{e}^{swept}_{x_{optical}}\right) = (R_{y_{specimen}})_{max}}$	260<br>236	260<br>236	260<br>236	260<br>236	260<br>236	260<br>236	260<br>236	260<br>236	260<br>236	260<br>236
$\boldsymbol{NA^{max}_{exc}}$	0.21	0.24	0.375	0.37	0.40	0.475	0.47	0.538	0.538	0.60<br>0.70
$\boldsymbol{R\left(\hat{e}_{z_{optical}}\right)}$	1162	1017	651	659	610	514	519	454	454	407<br>349
$\boldsymbol{(R_{z_{optical}})_{max}}$	577<br>505	539<br>475	415<br>376	419<br>379	398<br>362	355<br>326	357<br>328	325<br>301	325<br>301	300<br>251
$\boldsymbol{R\left(\hat{e}_{yz_{diag}}\right)}$	254<br>232	252<br>230	241<br>222	242<br>223	239<br>220	232<br>215	232<br>215	226<br>210	226<br>210	219<br>196
$\boldsymbol{(NA^{max}_{exc})_{xeff}}$	N.A.	N.A.	0.375	0.40	N.A.	0.411	0.407	0.466	0.466	0.60<br>0.70
$\boldsymbol{R\left(\hat{e}^{SI}_{x_{optical}}\right)}$	N.A.	N.A.	186<br>173	182<br>170	N.A.	181<br>169	181<br>170	174<br>163	174<br>163	159<br>141
$\boldsymbol{(R_{x_{specimen}})_{max}}$	270<br>248	265<br>244	246<br>228	246<br>228	242<br>225	233<br>217	234<br>217	226<br>210	226<br>210	219<br>196
$\boldsymbol{(R_{z_{specimen}})_{max}}$	358<br>334	346<br>323	298<br>280	299<br>282	290<br>274	270<br>256	271<br>257	255<br>242	255<br>242	242<br>213

Table S2. Imaging conditions for the experimental light sheet characterization using living cells.

**Figure / Movie #**	**Light Sheet Description**	NA_{exc}	σ_{NA}	$NA_{annulus}$	y_{FWHM} (λ_{exc}/n)	Δx_{sp} **(nm) /** $z_{optical}$	**LLC-PK1 timeseries volume interval (sec)**	**Skewed imaged volume (**$y_{optical}$ **x** $x_{optical}$ **x** x_{sp} **µm^3)**	**mEGFP-tagged TUBA1B hiPSC photobleaching rate (**$\tau_{planes/\mu m}$**)**
Fig.S3/ Movie 1	Swept Gaussian	0.21	0.21	0.40/ 0.20	51	350 / 188	4.0	38 x 52 x 350	1229
Fig.S4/ Movie2	Swept sinc	0.32	N/A	0.40/ 0.20	51.5	270 / 145	3.5	38 x 52 x 270	1300
Fig.2B/ Movie 5	Hexagonal multi-Bessel lattice	0.43	N/A	0.47/ 0.40	48	230 / 123	3.3	38 x 52 x 230	1387
Fig.S9B/ Movie 3	Square multi-Bessel lattice	0.30	N/A	0.375/ 0.225	48.5	280 / 150	3.5	38 x 52 x 280	1068
Fig.S11/ Movie 15	Axial standing wave	0.30	0.10	0.40/ 0.20	54.5	280 / 150	3.5	38 x 52 x 280	982
Fig.S12/ Movie 16	Axially confined square lattice	0.30	0.09	0.40/ 0.20	54	280 / 150	3.3	38 x 52 x 280	857
Fig.S13/ Movie 17	Axially confined hexagonal lattice	0.40	0.075	0.60/ 0.20	52	240 / 129	3.1	38 x 52 x 240	917
Fig.3A/ Movie 6	Harmonic balanced hexagonal lattice	0.50	0.075	0.60/ 0.40	53	200 / 107	3.1	38 x 52 x 300	840
Fig.3B/ Movie 7	Harmonic balanced hexagonal-rectangular lattice	0.50	0.15	0.60/ 0.40	56	200 / 107	3.1	38 x 52 x 200	1230
Fig.S10/ Movie 4	Square multi-Bessel lattice	0.41	N/A	0.60/ 0.40	16	240 / 129	4.2	38 x 52 x 60	192
Fig.S25/ Movie 14	Axial standing wave	0.25	0.13	0.35/ 0.20	54.5	310 / 166	3.7	38 x 52 x 310	1213
Fig.S17/ Movie 18	Axial standing wave	0.45	0.13	0.60/ 0.20	52.5	220 / 118	3.5	38 x 52 x 220	N/A
Fig. S20/ Movie 10	Harmonic balanced hexagonal lattice-SIM	0.46	0.10	0.60/ 0.20	42.5	260 / 140	5.6	80 x 194 x 18	N/A
Fig.S22/ Movie 12	Swept Gaussian	0.21	0.42	0.60/ 0.20	14.5	350 / 188	4.4	38 x 52 x 88	285
Fig.S23/ Movie 13	Swept sinc	0.40	N/A	0.60/ 0.20	15	240 / 129	4.1	38 x 52 x 60	229

Supplemental Movie Captions

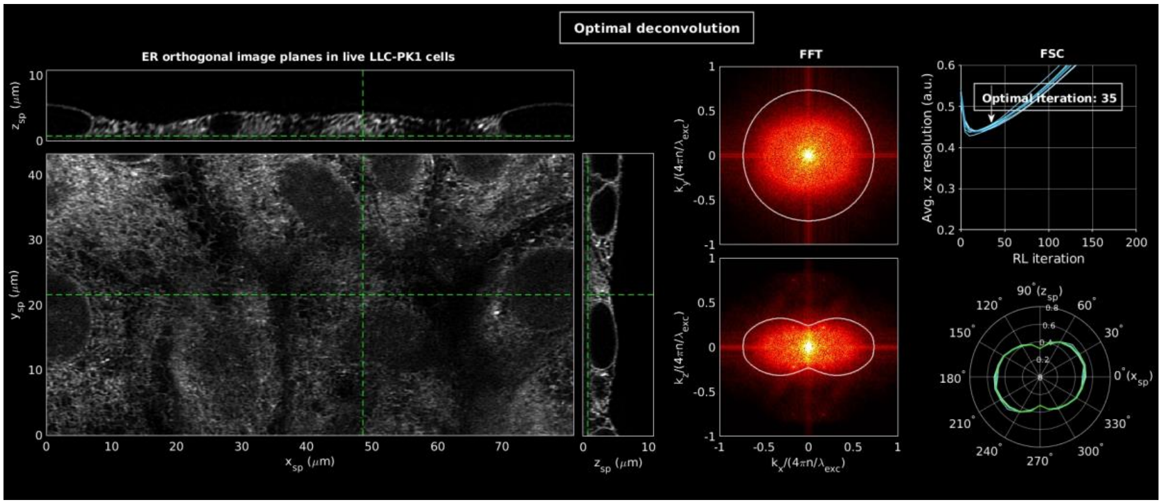

Movie 1. Imaging with the Gaussian light sheet of $\boldsymbol{\sigma_{NA} = 0.21}$, $\boldsymbol{y_{FWHM}} = 51.0\ \boldsymbol{\lambda_{exc}/n}$ in Fig. S3: Part 1, RL deconvolution of a simulated test pattern vs. iteration number; Part 2, RL deconvolution vs iteration number of mutually orthogonal orthoslices through an image volume of LLC-PK1 cells expressing a marker of the endoplasmic reticulum; Part 3, dynamics of the same cells for 100 time points at 4.0 sec intervals in a $\boldsymbol{xy_{sp}}$ maximum intensity projection and $\boldsymbol{xz_{sp}}$, $\boldsymbol{yz_{sp}}$ orthoslices; Part 4: 3D rendering of dynamics across the full field of cells for 100 time points at 3.0 sec intervals.

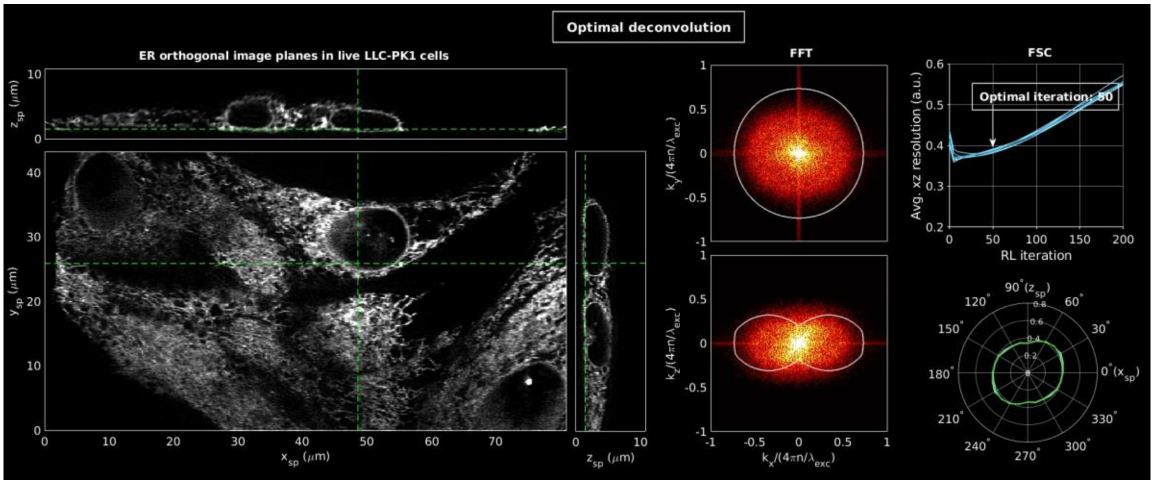

Movie 2. Imaging with the sinc light sheet of $\boldsymbol{NA_{sinc}} = 0.24$, $\boldsymbol{y_{FWHM}} = 53.5\ \boldsymbol{\lambda_{exc}/n}$ in Fig. S4. Movie description is otherwise identical to Movie 1.

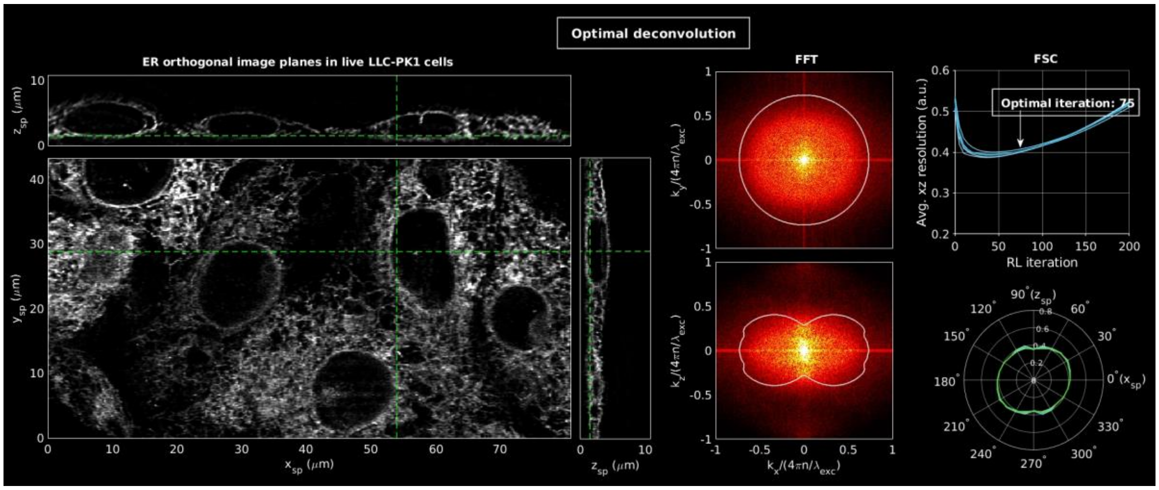

Movie 3. Imaging with the multi-Bessel square LLS of $\boldsymbol{NA_{exc}} = 0.30$, $\boldsymbol{NA_{annulus}} =$ 0.375/0.225, $\boldsymbol{y_{FWHM}} = 48.5\ \boldsymbol{\lambda_{exc}/n}$ of Fig. S9B. Movie description is otherwise identical to Movie 1.

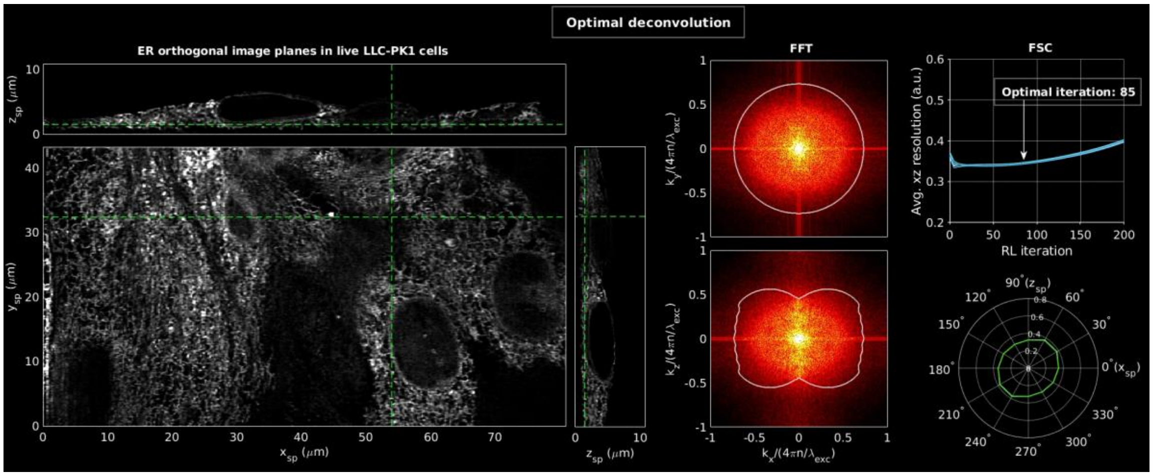

Movie 4. Imaging with the multi-Bessel square LLS of $\boldsymbol{NA_{exc}} = 0.41$, $\boldsymbol{NA_{annulus}} = 0.60/0.40$, $\boldsymbol{y_{FWHM}} = 16.0\ \boldsymbol{\lambda_{exc}/n}$ of Fig. S10. Movie description is otherwise identical to Movie 1.

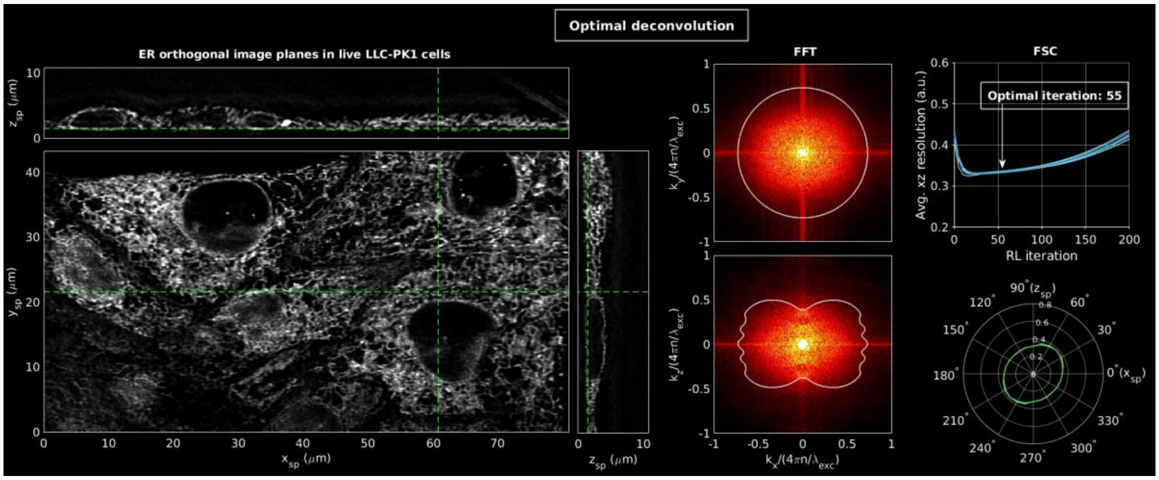

Movie 5. Imaging with the multi-Bessel hexagonal LLS of $\boldsymbol{NA_{exc}} = 0.43$, $\boldsymbol{NA_{annulus}} = 0.47/0.40$, $\boldsymbol{y_{FWHM}} = 48.0\ \boldsymbol{\lambda_{exc}/n}$ of Fig. 2B. Movie description is otherwise identical to Movie 1.

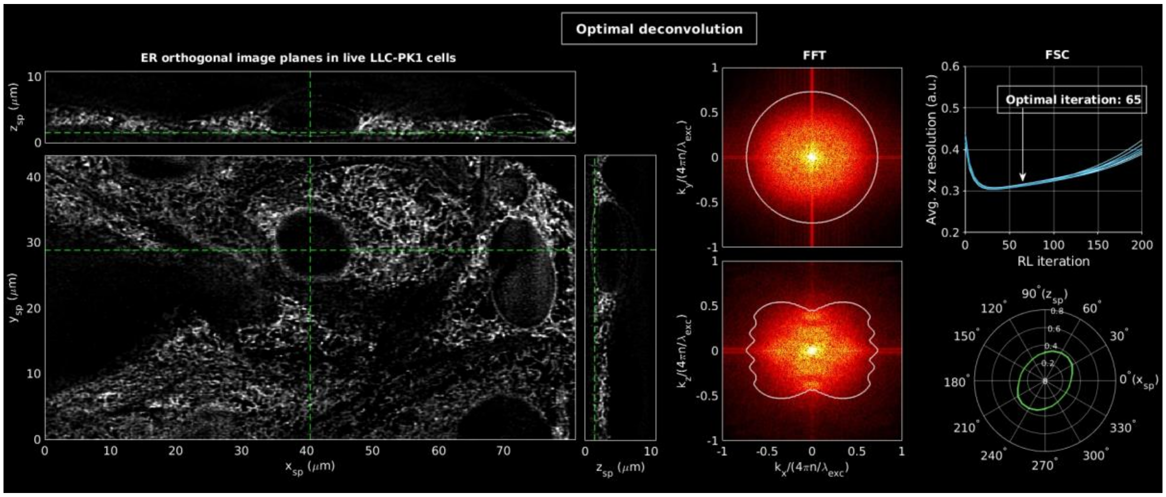

Movie 6. Imaging with the harmonic balanced hexagonal LLS of $\boldsymbol{NA_{exc}} = 0.50$, $\boldsymbol{\sigma_{NA}} = 0.075$, $\boldsymbol{y_{FWHM}} = 53.0\ \boldsymbol{\lambda_{exc}/n}$ of Fig. 3A. Movie description is otherwise identical to Movie 1.

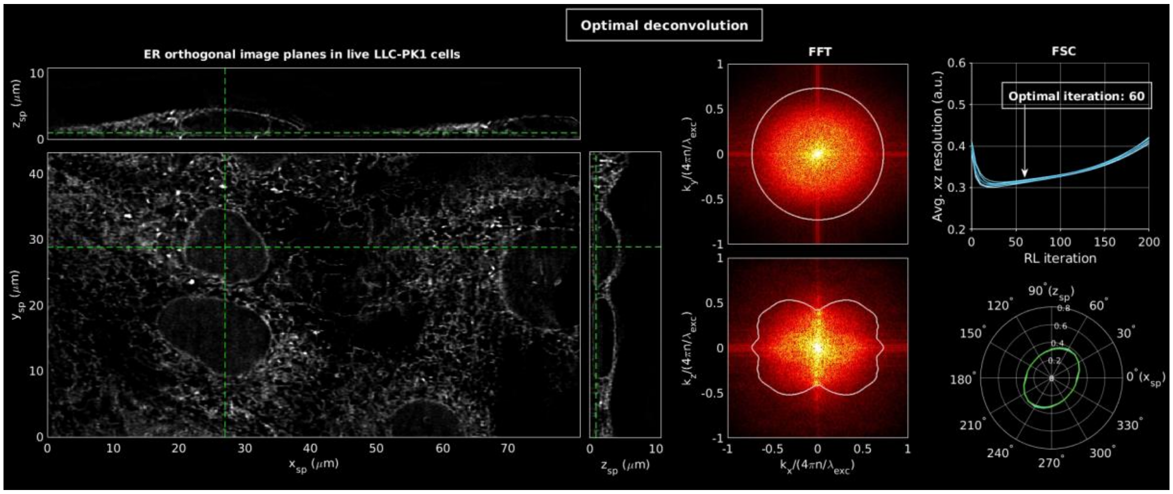

Movie 7. Imaging with the harmonic balanced hexrect pattern of $\boldsymbol{NA_{exc}} = 0.50$, $\boldsymbol{\sigma_{NA}} = 0.15$, $\boldsymbol{y_{FWHM}} = 56.0\ \boldsymbol{\lambda_{exc}/n}$ of Fig. 3B. Movie description is otherwise identical to Movie 1.

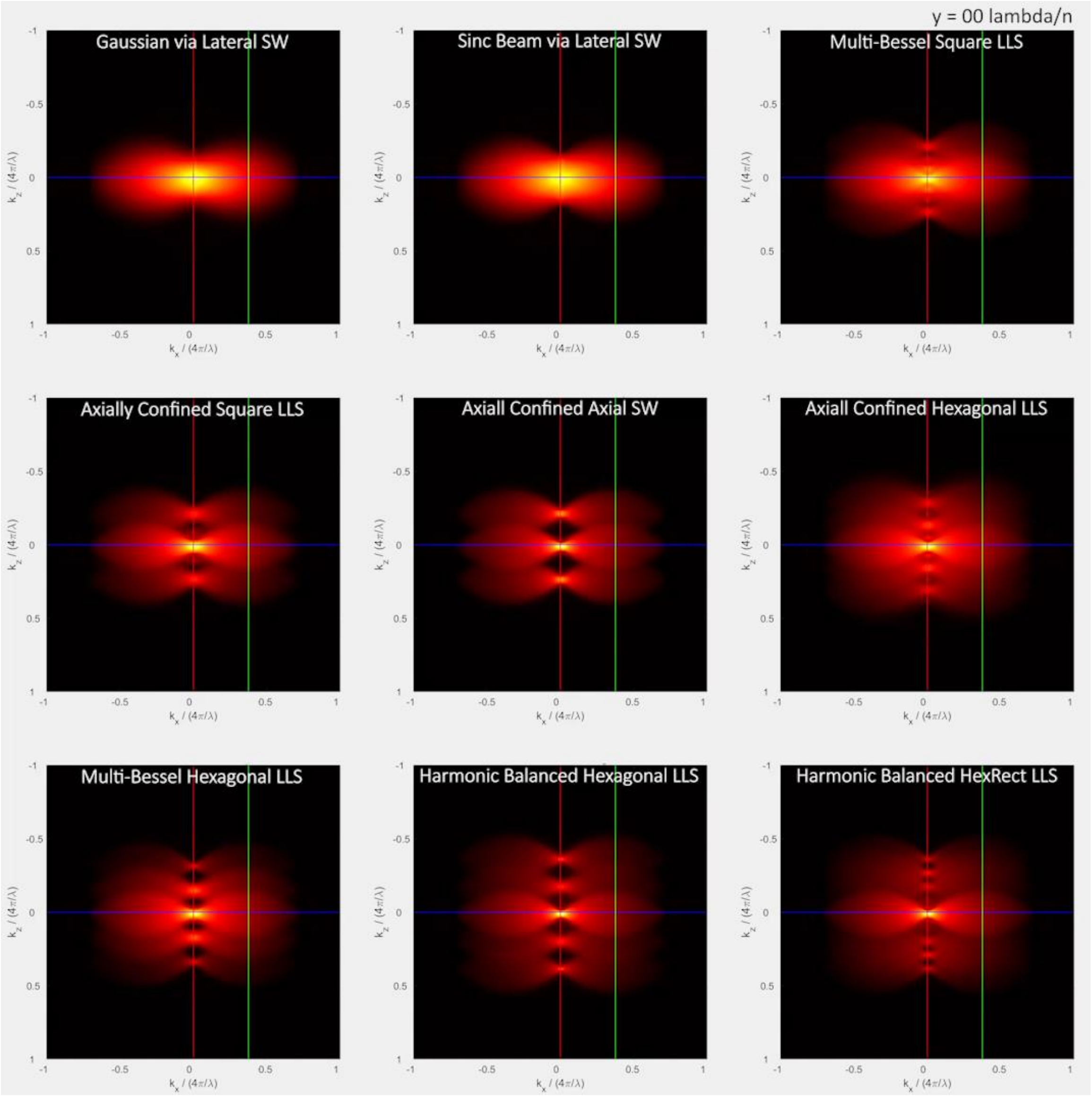

Movie 8. Comparison of the evolution of the overall OTF with increasing distance $\boldsymbol{y}$ along the propagation direction for Gaussian, sinc, and seven lattice light sheets, all of length $\boldsymbol{y_{FWHM}}$~50 $\boldsymbol{\lambda_{exc}/n}$.

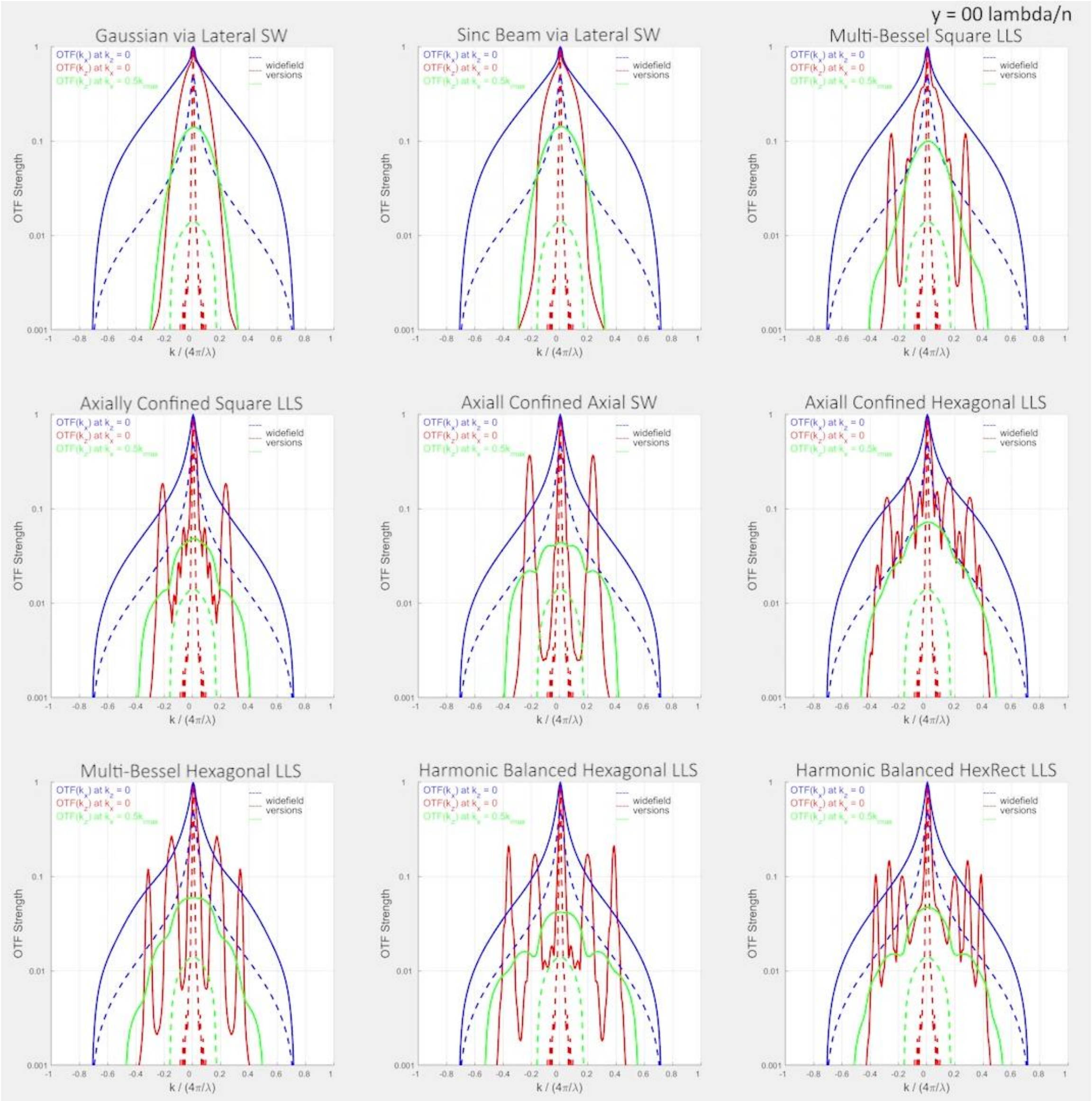

Movie 9. Comparison of the evolution of different linecuts through the overall OTF with increasing distance $\boldsymbol{y}$ along the propagation direction for Gaussian, sinc, and seven lattice light sheets, all of length $\boldsymbol{y_{FWHM}}$~50 $\boldsymbol{\lambda_{exc}/n}$.

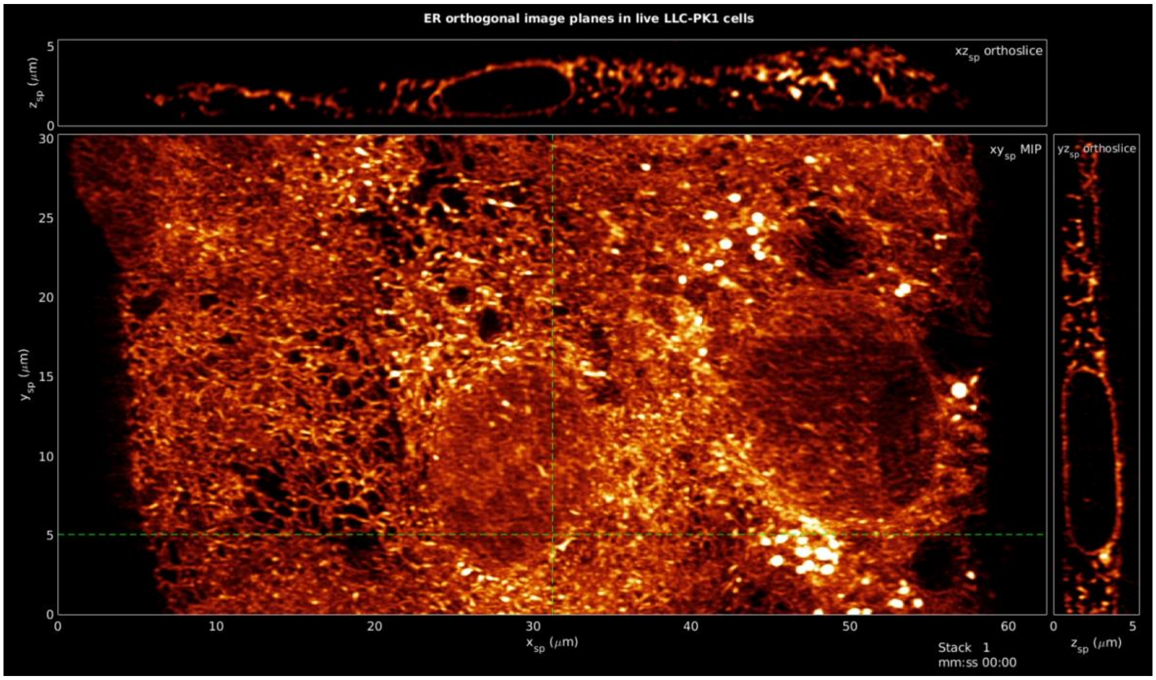

Movie 10. 3D dynamics of the ER in living LLC-PK1 cells over a volume of 80 x 194 x 18 um for 100 volumes at 5.6 sec intervals as imaged by LLS structured illumination with a harmonic balanced hexagonal LLS of $\boldsymbol{NA_{exc}} =$ 0.46 at a xyz resolution (Eqs. (S10h-k)) in specimen coordinates (Figs. S2B,C) of 235 x 183 x 273 nm.

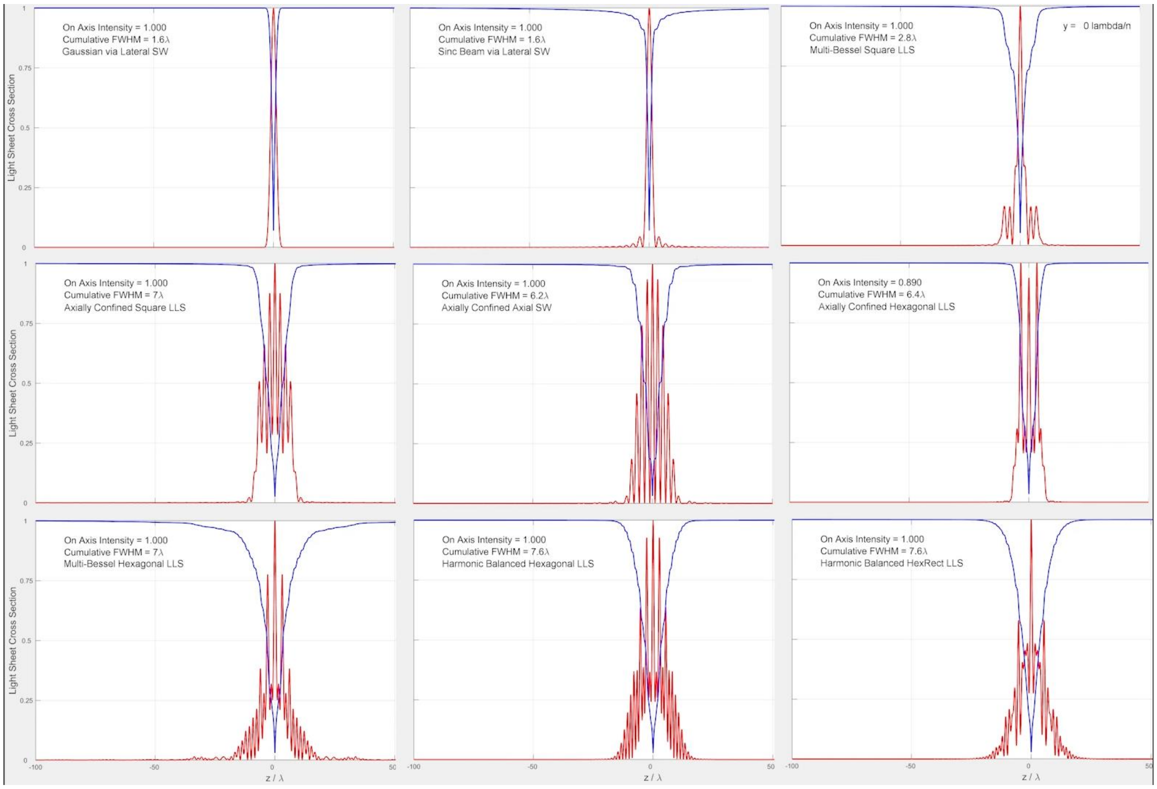

Movie 11. Comparison of the evolution of the cross-sectional profile and integrated intensity profile with increasing distance $\boldsymbol{y}$ along the propagation direction for Gaussian, sinc, and seven lattice light sheets, all of length $\boldsymbol{y_{FWHM}}$~50 $\boldsymbol{\lambda_{exc}/n}$ ..

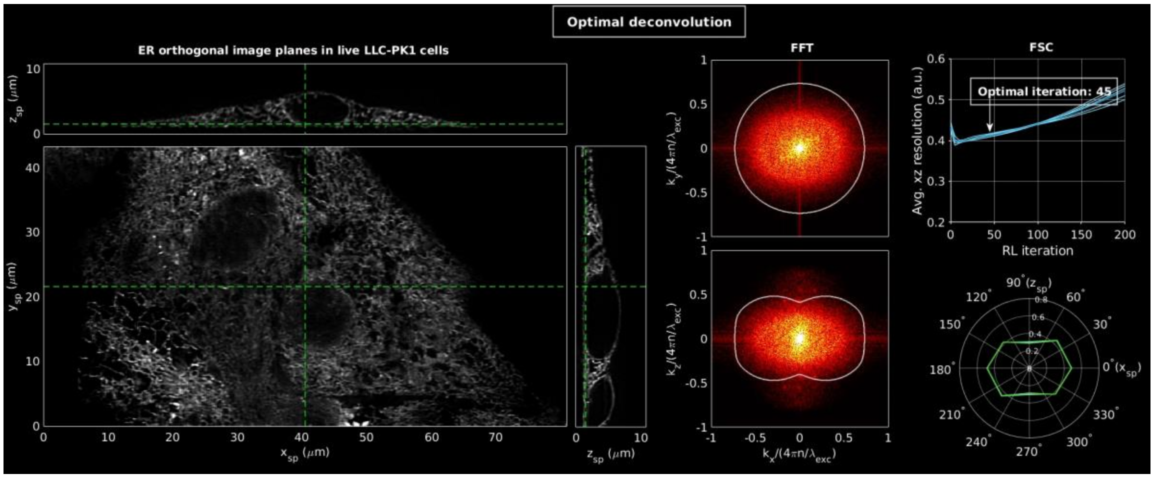

Movie 12. Imaging with the Gaussian light sheet of $\boldsymbol{\sigma_{NA} = 0.42}, \boldsymbol{y_{FWHM}} = 14.5\ \boldsymbol{\lambda_{exc}/n}$ in Fig. S22. Movie description is otherwise identical to Movie 1.

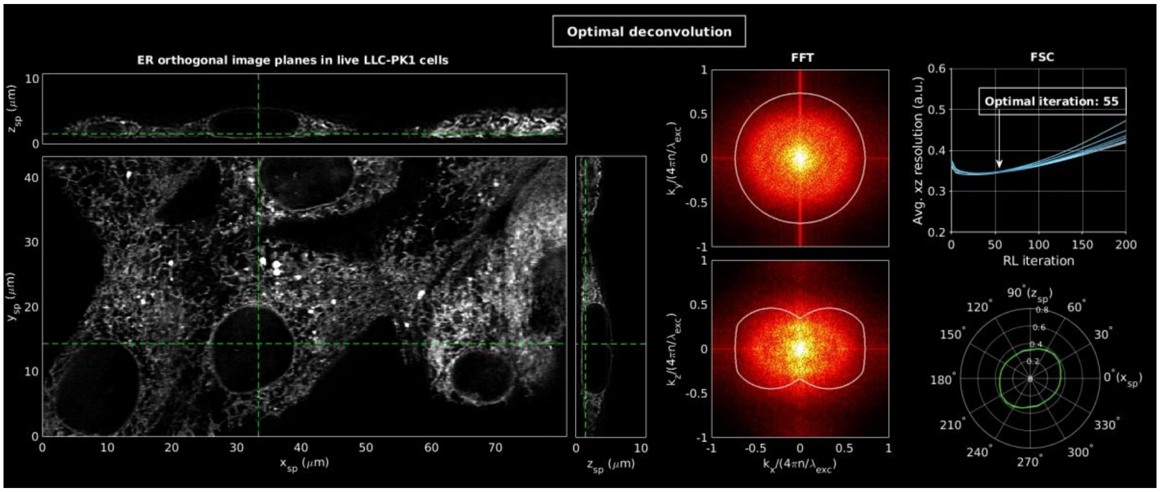

Movie 13. Imaging with the sinc light sheet of $\boldsymbol{NA_{sinc}} = 0.45$, $\boldsymbol{y_{FWHM}} = 15.0\ \boldsymbol{\lambda_{exc}/n}$ in Fig. S23. Movie description is otherwise identical to Movie 1.

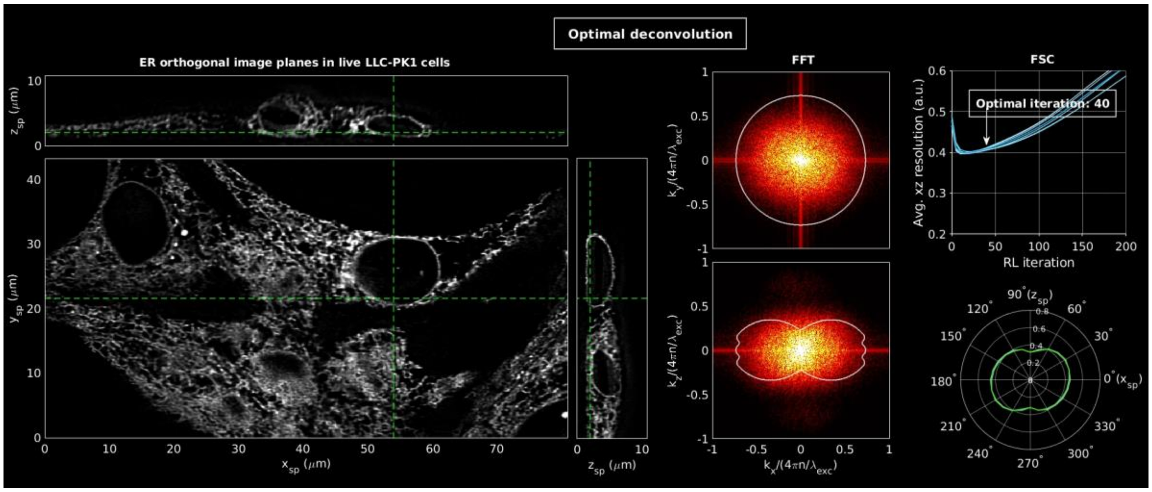

Movie 14. Imaging with the axially confined axial standing wave light sheet, $\boldsymbol{NA_{exc}} = 0.25$, $\boldsymbol{\sigma_{NA}} = 0.13$, $\boldsymbol{y_{FWHM}} = 54.5\ \boldsymbol{\lambda_{exc}/n}$ of Fig. S25. Movie description is otherwise identical to Movie 1.

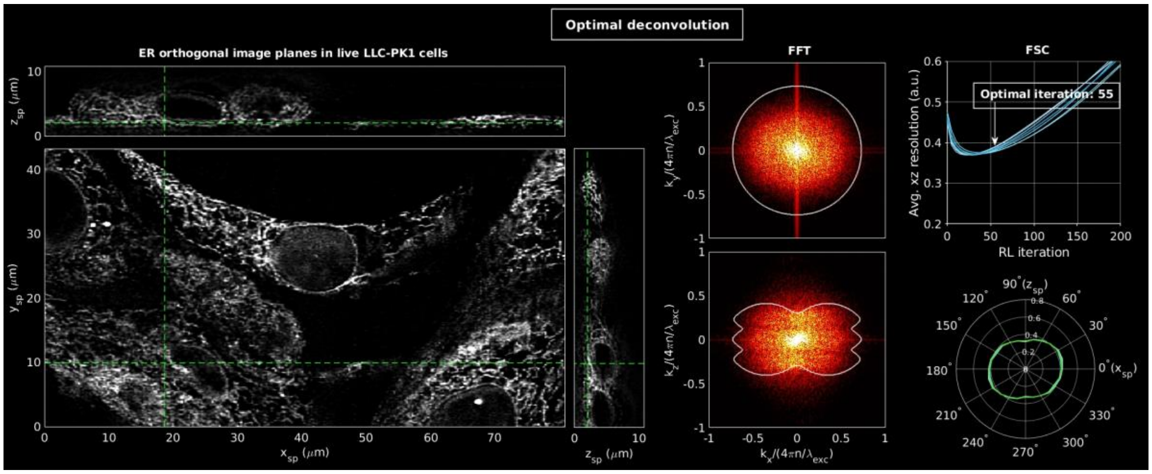

Movie 15. Imaging with the axially confined axial standing wave light sheet, $\boldsymbol{NA_{exc}} = 0.30$, $\boldsymbol{\sigma_{NA}} = 0.10$, $\boldsymbol{y_{FWHM}} = 54.5\ \boldsymbol{\lambda_{exc}/n}$ of Fig. S11. Movie description is otherwise identical to Movie 1.

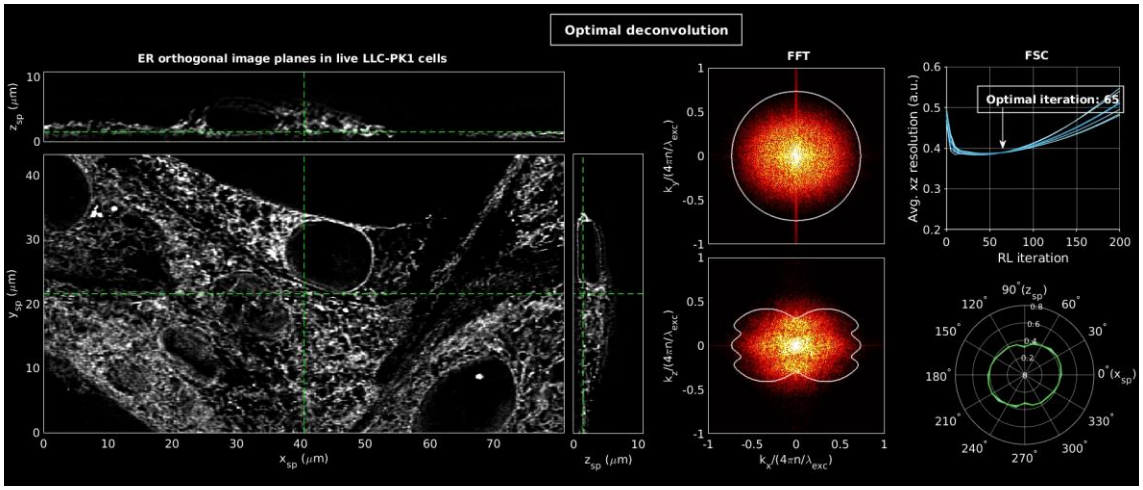

Movie 16. Imaging with the axially confined square LLS, $\boldsymbol{NA_{exc}} = 0.30$, $\boldsymbol{\sigma_{NA}} = 0.090$, $\boldsymbol{y_{FWHM}} = 54.0\ \boldsymbol{\lambda_{exc}/n}$ of Fig. S12. Movie description is otherwise identical to Movie 1.

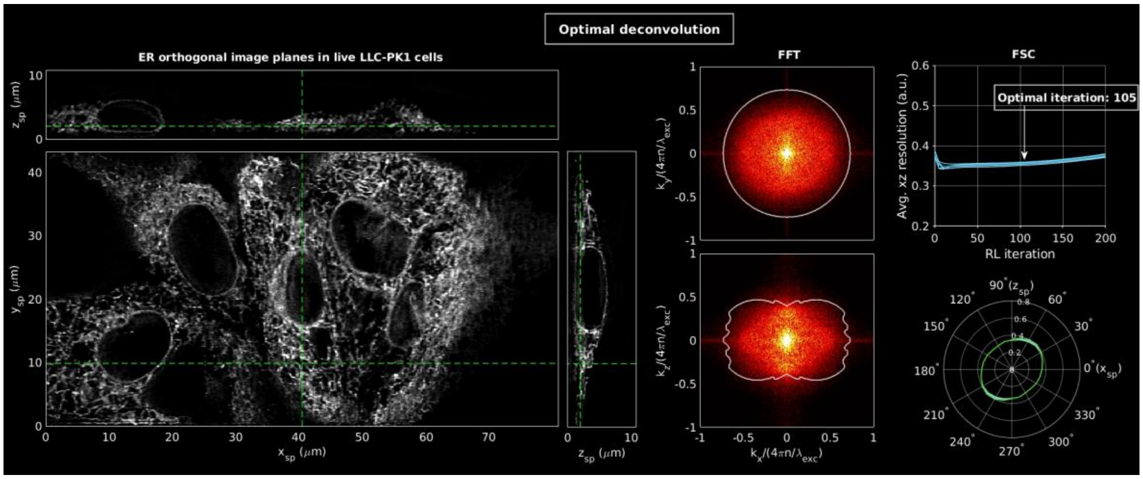

Movie 17. Imaging with the axially confined hexagonal LLS, $\boldsymbol{NA_{exc}} = 0.40$, $\boldsymbol{\sigma_{NA}} = 0.075$, $\boldsymbol{y_{FWHM}} = 52.0\ \boldsymbol{\lambda_{exc}/n}$ of Fig. S13. Movie description is otherwise identical to Movie 1.

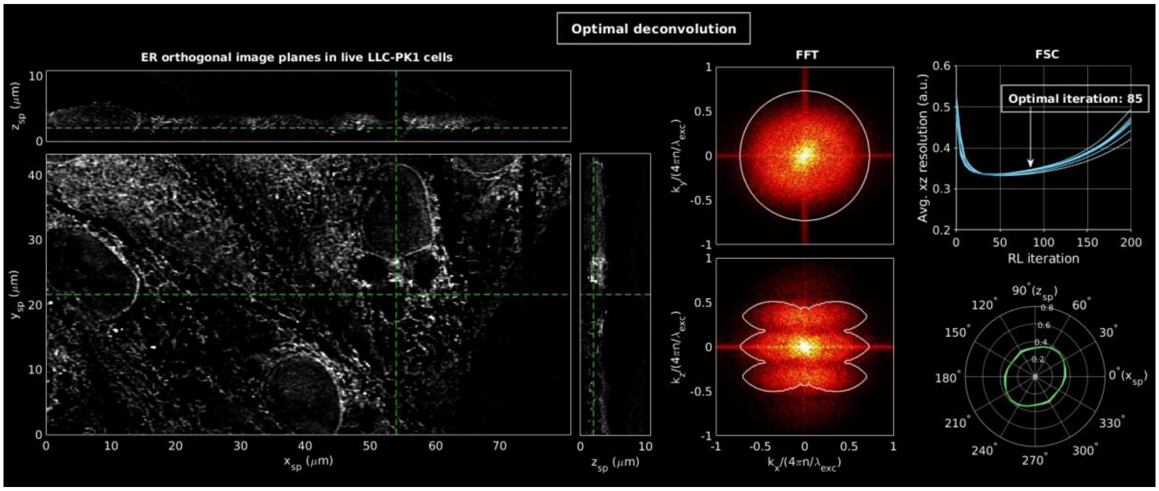

Movie 18. Imaging with the axially confined axial standing wave light sheet, $\boldsymbol{NA_{exc}} = 0.45$, $\boldsymbol{\sigma_{NA}} = 0.065$, $\boldsymbol{y_{FWHM}} = 52.5\ \boldsymbol{\lambda_{exc}/n}$ of Fig. S17. Movie description is otherwise identical to Movie 1.

REFERENCES AND NOTES

1. E. H. K. Stelzer, F. Strobl, B.-J. Chang, F. Preusser, S. Preibisch, K. McDole, R. Fiolka, Light sheet fluorescence microscopy. *Nat. Rev. Method Prime* **1**, 73 (2021).

2. B. C. Chen, W. R. Legant, K. Wang, L. Shao, D. E. Milkie, M. W. Davidson, C. Janetopoulos, X. S. Wu, John A Hammer III, Z. Liu, B. P. English, Y. Mimori-Kiyosue, D. P. Romero, A. T. Ritter, J. Lippincott-Schwartz, L. Fritz-Laylin, R Dyche Mullins, D. M. Mitchell, J. N. Bembenek, A.-C. Reymann, R. Böhme, S. W. Grill, J. T. Wang, G. Seydoux, U Serdar Tulu, D. P. Kiehart, E. Betzig, Lattice light-sheet microscopy: Imaging molecules to embryos at high spatiotemporal resolution. *Science* **346**, 1257998 (2014).

3. A. T. Ritter, Y. Asano, J. C. Stinchcombe, N. M. G. Dieckmann, B. C. Chen, C. Gawden-Bone, S. van Engelenburg, W. Legant, L. Gao, M. W. Davidson, E. Betzig, J. Lippincott-Schwartz, G. M. Griffiths, Actin depletion initiates events leading to granule secretion at the immunological synapse. *Immunity* **42**, 864–876 (2015).

4. N. Yamashita, M. Morita, W. R. Legant, B.-C. Chen, E. Betzig, H. Yokota, Y. Mimori-Kiyosue, Three-dimensional tracking of plus-tips by lattice light-sheet microscopy permits the quantification of microtubule growth trajectories within the mitotic apparatus. *J. Biomed. Opt.* **20**, 101206 (2015).

5. A. M. Valm, S. Cohen, W. R. Legant, J. Melunis, U. Hershberg, E. Wait, A. R. Cohen, M. W. Davidson, E. Betzig, J. Lippincott-Schwartz, Applying systems-level spectral imaging and analysis to reveal the organelle interactome. *Nature* **546**, 162−167 (2017).

6. D. M. Veltman, T. D. Williams, G. Bloomfield, B. C. Chen, E. Betzig, R. H. Insall, R. R. Kay, A plasma membrane template for macropinocytic cups. *eLife* **5**, e20085 (2016).

7. T. L. Liu, S. Upadhyayula, D. E. Milkie, V. Singh, K. Wang, I. A. Swinburne, K. R. Mosaliganti, Z. M. Collins, T. W. Hiscock, J. Shea, A. Q. Kohrman, T. N. Medwig, D. Dambournet, R. Forster, B. Cunniff, Y. Ruan, H. Yashiro, S. Scholpp, E. M. Meyerowitz, D. Hockemeyer, D. G. Drubin, B. L. Martin, D. Q. Matus, M. Koyama, S. G. Megason, T. Kirchhausen, E. Betzig, Observing the cell in its native state: Imaging subcellular dynamics in multicellular organisms. *Science* **360**, eaaq1392 (2018).

8. J. J. Chen, Z. Zhang, L. Li, B.-C. Chen, A. Revyakin, B. Hajj, W. Legant, M. Dahan, T. Lionnet, E. Betzig, R. Tjian, Z. Liu, Single-molecule dynamics of enhanceosome assembly in embryonic stem cells. *Cell* **156**, 1274–1285 (2014).

9. J. T. Wang, J. Smith, B. C. Chen, H. Schmidt, D. Rasoloson, A. Paix, B. G. Lambrus, D. Calidas, E. Betzig, G. Seydoux, Regulation of RNA granule dynamics by phosphorylation of serine-rich, intrinsically-disordered proteins in C-elegans. *eLife* **3**, e04591 (2014).

10. B. J. Chang, K. M. Dean, R. Fiolka, Systematic and quantitative comparison of lattice and Gaussian light-sheets. *Opt. Express* **28**, 27052–27077 (2020).

11. B.-J. Chang, R. Fiolka, Light-sheet engineering using the Field Synthesis theorem. *J. Phys. Photon.* **2**, 014001 (2020).

12. E. Remacha, L. Friedrich, J. Vermot, F. O. Fahrbach, How to define and optimize axial resolution in light-sheet microscopy: A simulation-based approach. *Biomed. Opt. Express* **11**, 8–26 (2020).

13. M. G. L. Gustafsson, L. Shao, P. M. Carlton, C J Rachel Wang, I. N. Golubovskaya, W Zacheus Cande, D. A. Agard, J. W. Sedat, Three-dimensional resolution doubling in wide-field fluorescence microscopy by structured illumination. *Biophys. J.* **94**, 4957–4970 (2008).

14. D. Li, L. Shao, B. C. Chen, X. Zhang, M. Zhang, B. Moses, D. E. Milkie, J. R. Beach, J. A. Hammer III, M. Pasham, T. Kirchhausen, M. A. Baird, M. W. Davidson, P. Xu, E. Betzig, Extended-resolution structured illumination imaging of endocytic and cytoskeletal dynamics. *Science* **349**, aab3500 (2015).

15. B.-J. Chang, M. Kittisopikul, K. M. Dean, P. Roudot, E. S. Welf, R. Fiolka, Universal light-sheet generation with field synthesis. *Nat. Methods* **16**, 235−238 (2019).

16. R. E. Betzig (U.S. patent no. US 9,891,421, 2018), vol. US 9,891,421.

17. R. E. Betzig (U.S. patent no. US 10,509,217, 2019), vol. US 10,509,217.

18. F. O. Fahrbach, A. Rohrbach, A line scanned light-sheet microscope with phase shaped self-reconstructing beams. *Opt. Express* **18**, 24229–24244 (2010).

19. F. O. Fahrbach, P. Simon, A. Rohrbach, Microscopy with self-reconstructing beams. *Nat. Photonics* **4**, 780–785 (2010).

20. T. A. Planchon, L. Gao, D. E. Milkie, M. W. Davidson, J. A. Galbraith, C. G. Galbraith, E. Betzig, Rapid three-dimensional isotropic imaging of living cells using Bessel beam plane illumination. *Nat. Methods* **8**, 417-423 (2011).

21. R. E. Betzig (U.S. patent no. US 9,448,395, 2016), vol. US 9,448,395.

22. R. E. Betzig (U.S. patent no. US 8,711,211, 2014), vol. US 8,711,211.

23. L. Gao, L. Shao, C. D. Higgins, J. S. Poulton, M. Peifer, M. W. Davidson, X. Wu, B. Goldstein, E. Betzig, Noninvasive imaging beyond the diffraction limit of 3D dynamics in thickly fluorescent specimens. *Cell* **151**, 1370–1385 (2012).

24. R. E. Betzig (U.S. patent no. US 9,223,125, 2015), vol. US 9,223,125.

25. E. Betzig, Sparse and composite coherent lattices. *Phys. Rev. A* **71**, (2005).

26. R. E. Betzig (U.S. patent no. US 7,894,136, 2011), vol. US 7,894,136.

27. W. R. Legant, L. Shao, J. B. Grimm, T. A. Brown, D. E. Milkie, B. B. Avants, L. D. Lavis, E. Betzig, High-density three-dimensional localization microscopy across large volumes. *Nat. Methods* **13**, 359–365 (2016).

28. J. Bewersdorf, R. Schmidt, S. W. Hell, Comparison of I^5M and 4Pi-microscopy. *J. Microsc.* **222**, 105–117 (2006).

29. M. Nagorni, S. W. Hell, Coherent use of opposing lenses for axial resolution increase in fluorescence microscopy I. Comparative study of concepts. *J. Opt. Soc. Am. A.* **18**, 36–48 (2001).

30. M. Nagorni, S. W. Hell, Coherent use of opposing lenses for axial resolution increase II. Power and limitation of nonlinear image restoration. *J. Opt. Soc. Am. A.* **18**, 49–54 (2001).

31. S. Hell, E. H. K. Stelzer, Properties of a 4Pi confocal fluorescence microscope. *J. Opt. Soc. Am. A* **9**, 2159–2166 (1992).

32. B. Bailey, D. L. Farkas, D. L. Taylor, F. Lanni, Enhancement of axial resolution in fluorescence microscopy by standing-wave excitation. *Nature* **366**, 44–48 (1993).

33. M. G. L. Gustafsson, D. A. Agard, J. W. Sedat, (IM)-M-5: 3D widefield light microscopy with better than 100 nm axial resolution. *J. Microsc.* **195**, 10–16 (1999).

34. K. M. Dean, P. Roudot, E. S. Welf, G. Danuser, R. Fiolka, Deconvolution-free subcellular imaging with axially swept light sheet microscopy. *Biophys. J.* **108**, 2807–2815 (2015).

35. F. Aguet, Super-resolution fluorescence microscopy based on physical models. (2009).

36. B. Richards, E. Wolf, Electromagnetic diffraction in optical systems, II. Structure of the image field in an aplanatic system. *Proc. R. Soc. London Ser. A. Math. Phys. Sci.* **253**, 358–379 (1959).

37. D. S. Biggs, M. Andrews, Acceleration of iterative image restoration algorithms. *Appl. Optics* **36**, 1766–1775 (1997).

38. L. B. Lucy, An iterative technique for the rectification of observed distributions. *Astron. J* **79**, 745 (1974).

39. W. H. Richardson, Bayesian-based iterative method of image restoration. *J. Opt. Soc. Am.* **62**, 55−59 (1972).

40. S. Koho, G. Tortarolo, M. Castello, T. Deguchi, A. Diaspro, G. Vicidomini, Fourier ring correlation simplifies image restoration in fluorescence microscopy. *Nat. Commun.* **10**, 3103 (2019).

41. N. Ji, D. E. Milkie, E. Betzig, Adaptive optics via pupil segmentation for high-resolution imaging in biological tissues. *Nat. Methods* **7**, 141–147 (2010).

42. H. I. Campbell, S. Zhang, A. H. Greenaway, S. Restaino, Generalized phase diversity for wave-front sensing. *Opt. Lett.* **29**, 2707–2709 (2004).

43. R. W.Gerchberg, A practical algorithm for the determination of the phase from image and diffraction plane pictures. *Optik* **35**, 237–246 (1972).

44. M. Van Heel, M. Schatz, Fourier shell correlation threshold criteria. *J. Struct. Biol.* **151**, 250–262 (2005).

45. D. P. Hoffman, E. Betzig, Tiled reconstruction improves structured illumination microscopy. *bioRxiv*, 2020.01.06.895318 (2020).

46. R. Szeliski, Image alignment and stitching: A tutorial. *Found Trends Comput.* **2**, 1–109 (2006).
